# Morphology remodelling and membrane channel formation in synthetic cells via reconfigurable DNA nanorafts

**DOI:** 10.1038/s41563-024-02075-9

**Published:** 2025-01-13

**Authors:** Sisi Fan, Shuo Wang, Longjiang Ding, Thomas Speck, Hao Yan, Stephan Nussberger, Na Liu

**Affiliations:** 1 2nd Physics Institute, University of Stuttgart, Stuttgart, Germany; 2 Max Planck Institute for Solid State Research, Stuttgart, Germany; 3 Department of Biophysics, Institute of Biomaterials and Biomolecular Systems, University of Stuttgart, Stuttgart, Germany; 4 Institute for Theoretical Physics IV, University of Stuttgart, Stuttgart, Germany; 5 Biodesign Center for Molecular Design and Biomimetics, Arizona State University, Tempe, AZ USA

**Keywords:** Self-assembly, DNA nanostructures

## Abstract

The shape of biological matter is central to cell function at different length scales and determines how cellular components recognize, interact and respond to one another. However, their shapes are often transient and hard to reprogramme. Here we construct a synthetic cell model composed of signal-responsive DNA nanorafts, biogenic pores and giant unilamellar vesicles (GUVs). We demonstrate that reshaping of DNA rafts at the nanoscale can be coupled to reshaping of GUVs at the microscale. The nanorafts collectively undergo reversible transitions between isotropic and short-range local order on the lipid membrane, programmably remodelling the GUV shape. Assisted by the biogenic pores, during GUV shape recovery the locally ordered DNA rafts perforate the membrane, forming sealable synthetic channels for large cargo transport. Our work outlines a versatile platform for interfacing reconfigurable DNA nanostructures with synthetic cells, expanding the potential of DNA nanotechnology in synthetic biology.

## Abstract (web summary)

The shape of biological matter is central to their function and interaction with other cellular components. A combination of DNA origami nanorafts with biogenic pores reversibly controls the shape and permeability of lipid vesicles at the microscale.

## Main

The plasma membrane defines the physical boundary of a cell, separating the cytoplasmic components of the cell from the external environment. It comprises primarily lipids and proteins. While the physical nature of lipids forms the basis of membrane flexibility, membrane proteins sustain the cell’s chemical climate by assisting the transfer of molecules across the membrane^1^. In addition, both lipids and proteins are responsible for the cell’s morphology and distinct changes in membrane shape^2–5^. The exquisitely coordinated interplay between membrane shape and the function of membrane proteins has been of great scientific and technological interest^6–8^. To understand cells, the bottom-up approach in synthetic biology follows the ‘understanding by building’ route to construct artificial mimics de novo^9–13^, which capture the essence of their biological counterparts. DNA has proven to be an ideal construction material in this field^14^ due to its sequence-specific and predictable interactions following Watson–Crick base pairing^15,16^. Crescent-shaped DNA origami structures^17^ have been created to mimic BAR domain proteins^18^ to remodel the cell surface landscape by imprinting their curved shapes onto membranes. Tension-loaded DNA clamps, inspired by proteins such as ESCRT-III and dynamin, have been developed to drive membrane tubulation^19^. Besides membrane sculpting^20–25^, great efforts have also been devoted to construct channel protein mimics made of DNA that possess a large pore size or a nanomechanical lid for controlled molecular transport across lipid membranes^26–33^.

These works have underpinned DNA nanotechnology as a key player in building biomimetic modules for synthetic cells. However, it remains a grand scientific and intellectual challenge to completely replicate the structure and function of cells^34^. In addition, our current understanding of many cellular processes is still inadequate. For instance, in 2023 Degen et al.^35^ discovered that plasma membrane rupture in different forms of cell death does not occur passively, as previously thought. Instead, it is an active and programmed process mediated by the plasma-membrane protein ninjurin-1, which aggregates to form a tightly packed, fence-like pore, leading to membrane rupture and eventually cell death. Such groundbreaking works are not only a spectacular twist to our long-believed biological phenomenon^36,37^, but also thought-provoking for synthetic biology. DNA nanotechnologists may ask whether rather than a complete replication of cells, one can instead design synthetic DNA-based platforms with greater freedom but reduced complexity^38–41^. This will empower a new way of thinking to create fully artificial DNA structures with engineered features, which do not necessarily have biological equivalents but can function in biological environments.

**Fig. 1:**
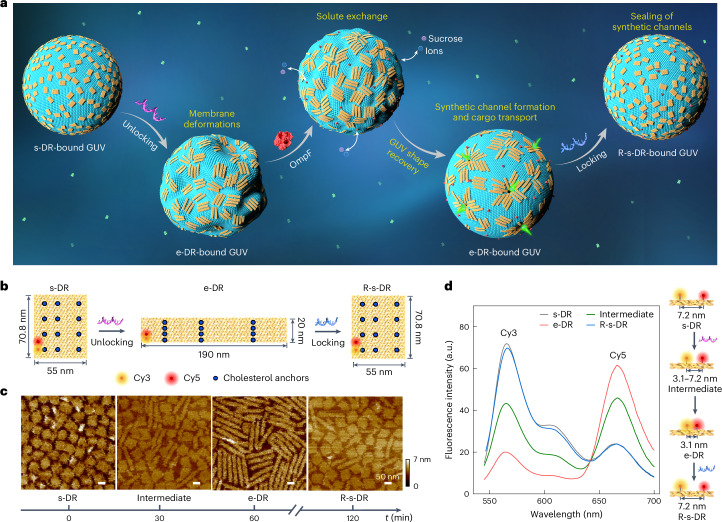
Reconfigurable DNA rafts working on lipid membranes. **a**, Schematic of the reconfigurable DNA nanorafts interfacing with a synthetic cell, assisted by biogenic pores, OmpF. **b**, Schematic of the reversible conformation changes of the DNA origami-based nanoraft from the s-DR to e-DR state by adding unlocking strands, and to the R-s-DR state by adding locking strands. Twelve cholesterol anchors (blue dots) and one FRET pair (Cy3 and Cy5) are positioned on the origami surface. **c**, AFM images of the DNA rafts on an SLB at different states. **d**, Fluorescence spectra (excitation wavelength, 532 nm) of the DNA raft-bound GUVs in an ensemble at different states. Source data

In this article, we demonstrate signal-responsive DNA nanorafts that can programmably interact with synthetic cells to remodel their membrane shapes, perforate the lipid membranes and regulate cargo flux (Fig. 1a). Our rationally designed cell model consists of hybrid modules, including membrane-bound reconfigurable DNA nanorafts, bacterial outer membrane proteins (OmpF) and a giant unilamellar vesicle (GUV). Their mutual interactions enable cellular behaviour and function without biological equivalents. We showcase that reshapings of DNA rafts and GUVs have a mutual impact on one another. Specifically, the DNA rafts can reconfigure with large aspect ratio alterations of between 1.3 and 9.5 on the GUV membrane driven by toehold-mediated strand-displacement reactions. Along with their conformation changes, the DNA rafts interact among each other through excluded volume and self-arrange in domains, undergoing a transition from disorder (isotropic) to short-range tetratic order and vice versa. Through such transitions, the DNA rafts impose reversible deformation and recovery of the GUV morphology, translating conformational changes of the DNA rafts at the nanoscale into engineered behaviour of the GUVs at the microscale. In addition, we systematically unravel the impact of DNA raft density, cholesterol-anchor number and pattern on the DNA raft, as well as the effect of osmotic pressure difference on the degree of GUV reshaping.

Most strikingly, the self-arranged DNA rafts that form locally ordered architectures can perforate the membrane during the GUV shape recovery in cooperation with biogenic pores, such as OmpF, creating synthetic channels from originally non-spanning DNA rafts. These synthetic channels enable cellular transport of large cargo (up to ~70 kDa) across the membrane. Crucially, these synthetic channels can be sealed on demand by reconfiguring the DNA rafts back to their initial conformation, thereby achieving engineerable control over membrane permeability.

## Reconfigurable DNA rafts working on lipid membranes

We use a DNA origami-based nanoraft design that can undergo reversible conformation changes with large aspect ratio alteration between square and rectangle shapes driven by toehold-mediated strand-displacement reactions (Fig. 1b). More specifically, the nearly square DNA rafts (s-DRs, 70.8 nm × 55 nm; aspect ratio, ~1.3) can be reconfigured to elongated rectangular DNA rafts (e-DRs, 190 nm × 20 nm; aspect ratio, ~9.5) upon the addition of unlocking DNA strands, whereas the reversible process is driven by locking DNA strands (Fig. 1b and Supplementary Figs. 1–3). To make a clear distinction, the square DNA rafts transformed back from the e-DRs are named R-s-DRs. Elongated rectangular DNA rafts that are assembled directly rather than via reconfiguration from the s-DRs are named post-e-DRs. For membrane binding, 12 cholesterol-tagged DNA anchors are extended from the bottom surface of the origami (Supplementary Figs. 4–6). Crucially, the cholesterol pattern formed on the origami surface also reversibly adapts to the conformation changes of the DNA raft (Fig. 1b).

The reconfiguration of DNA rafts at different states is first characterized on supported lipid bilayers (SLBs) by atomic force microscopy (AFM) under a relatively low Mg^2+^ concentration (5 mM) (Supplementary Figs. 7 and 8) to reduce non-specific electrostatic adsorption of the DNA rafts on the membrane. As shown in Fig. 1c and Supplementary Fig. 9, the s-DRs of low aspect ratio (1.3) display a disordered arrangement on the SLB. After the addition of unlocking strands, the s-DRs are almost all transformed to e-DRs. The e-DRs of high aspect ratio (9.5) are prone to align side by side through rearrangements on the membrane, creating local order. Such short-range ordering can be theoretically^42^ explained using a two-dimensional system of Brownian hard needle-like objects^43^. The formation of the locally ordered state with the coalignment of the e-DRs correlates with the inhibition of translational and rotational diffusion of the e-DRs in the direction normal to their long axes. The subsequent addition of locking strands triggers the transformation from the e-DRs to R-s-DRs, returning to disorder. Very crucially, in the case of the post-e-DRs, no prominent local order is created despite them having the same high aspect ratio as the e-DRs (Supplementary Fig. 10). This indicates that the dynamic reconfiguration from the s-DRs to e-DRs on the membrane, which drives the rearrangement of the DNA rafts, is a key step to induce local order in our system.

Subsequently, the s-DRs are incubated with GUVs (1,2-dioleoyl-*sn*-glycero-3-phosphocholine (DOPC), 0.05 mol% Atto655-DOPE, Supplementary Figs. 11–13). After reaching equilibrium, the transformations of the DNA rafts on GUVs are characterized by fluorescence resonance energy transfer (FRET). Two dyes (Cy3 and Cy5) are incorporated on the DNA origami (Fig. 1b and Supplementary Fig. 4). When reconfiguring from the s-DR (grey) to e-DR state (red), the relative distance of the two dyes varies from ~7.2 nm to ~3.1 nm, causing an decrease in Cy3 intensity and an increase in Cy5 intensity (Fig. 1d). The trend is then reversed when transforming from the e-DR (red) back to the R-s-DR (blue) state. The slight discrepancy between the grey and blue curves is probably due to photobleaching of the dyes and imperfections in the DNA structures.

## Correlation between reshapings of the DNA rafts and GUVs

**Fig. 2:**
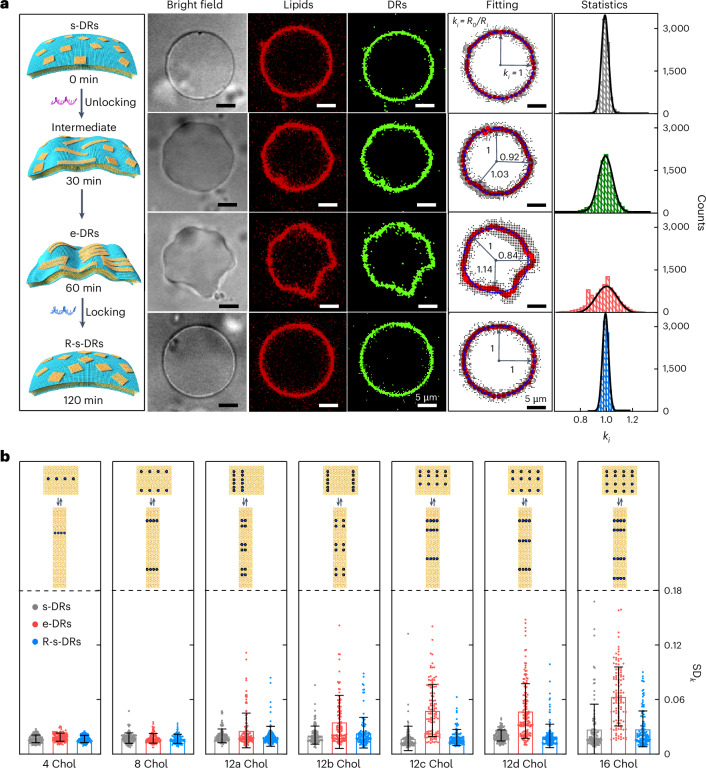
Correlation between reshaping of the DNA rafts and reshaping of GUVs. **a**, Left: schematic of deformation and recovery of the GUV morphology imposed by the DNA rafts, transforming among different states. Centre: bright-field and confocal fluorescence microscopy images of the GUVs with membrane-bound DNA rafts shown in different columns: lipid channel (Atto655, red); DNA raft channel (Atto488, green); shape tracing of the DNA raft-bound GUVs. Right: statistics of *k*_*i*_ at different states of the DNA rafts (data obtained from 152 GUVs per state). **b**, Influence of the cholesterol number and pattern on the degree of GUV remodelling. Data represent mean ± s.d. from three independent experiments. The GUV numbers (*n*) for s-DRs, e-DRs and R-s-DRs are as follows: 105, 105 and 105 in 4 Chol; 107, 107 and 107 in 8 Chol; 106, 115 and 117 in 12a Chol; 115, 118 and 107 in 12b Chol; 111, 110 and 109 in 12c Chol; 152, 152 and 152 in 12d Chol; and 109, 116 and 126 in 16 Chol, respectively. Source data

Next, we examine how the operation of the DNA rafts at the nanoscale is coupled to the behaviour of GUVs at the microscale. After incubation of the s-DRs (∼1.1 nM) with GUVs and reaching equilibrium, the surface density (*σ*) of s-DRs is ~60 µm^−2^ (Supplementary Fig. 14). Initially, the GUVs exhibit a spherical shape (Fig. 2a). The addition of unlocking strands that trigger the reconfiguration from the s-DRs to e-DRs gives rise to notable membrane deformations as early as 30 min, which become more prominent over time (Supplementary Videos 1 and 2). In a control experiment, in which DNA strands of non-specific sequences are added, no evident deformations of the s-DR-bound GUVs are observed (Supplementary Fig. 15 and Supplementary Video 1). In another control experiment, the post-e-DRs are directly bound to GUVs. The GUVs also remain spherical (Supplementary Fig. 16). These experimental results indicate that the reshaping of the GUV morphology is correlated with the reshaping process of the DNA rafts on the membrane. During the reconfiguration, the interactions among the e-DRs impart dynamic rearrangements of the e-DRs on the membrane to create local order. This generates steric pressure that can bend the membrane surfaces, reminiscent of protein crowding on membranes^44^ and submembrane protein scaffolds^2,7^. The local membrane deformation is then collectively converted into stress, inducing large-scale membrane remodelling, which is qualitatively supported by our theoretical model (Supplementary Fig. 17). Subsequently, by adding locking strands, disorder is created again through the transformation from the e-DRs to R-s-DRs. The deformed GUV is recovered to a spherical shape (Fig. 2a). By adding DNA strands of non-specific sequences, the GUV remains deformed without spherical shape recovery (Supplementary Fig. 18 and Supplementary Video 3). To provide insights, fluorescence recovery after photobleaching (FRAP) measurements are carried out on single GUVs bound with DNA rafts of different states. As shown in Supplementary Fig. 19, the e-DRs reveal very low mobility, whereas the s-DRs, R-s-DRs and post-e-DRs exhibit high mobility. In all the cases, the lipids remain diffusive. These results demonstrate that the s-DRs, R-s-DRs and post-e-DRs are highly mobile on the membrane. On the contrary, the interacting e-DRs self-arrange in local order display low mobility and can effectively sculpt the GUV morphology.

To quantitatively evaluate the degree of GUV reshaping at different states of the DNA rafts, normalized local curvatures ${k}_{i}=\frac{{R}_{0}}{{R}_{i}}$ are retrieved by shape tracing of the GUVs and fitting their contours (Supplementary Fig. 20 and Supplementary Video 4). *R*_0_ is the radius fitted to all spatial positions ${x}_{i}^{{\prime} }$ along a GUV trace and *R*_*i*_ is the distance between the centre of the fitted circle and ${x}_{i}^{{\prime} }$. The GUV shape is quantified by the standard deviation of all normalized local curvatures *k*_*i*_ (SD_*k*_). The mean value of SD_*k*_ of bare GUVs, 〈SD_*k*_〉_bare_ = 0.026 is used as the reference to calculate the membrane deformation efficiency. A GUV is considered deformed if its SD_*k*_ is higher than 〈SD_*k*_〉_bare_ (Supplementary Fig. 21). The histograms of ${k}_{i}$ are presented in Fig. 2a. At the s-DR state (Supplementary Fig. 22), *k*_*i*_ manifests a narrow distribution centred at 1.0. When transiting to the intermediate state (Supplementary Fig. 23) and then to the e-DR state (Supplementary Fig. 24), it becomes more and more broadened. After transforming back to the R-s-DR state (Supplementary Fig. 25), the distribution of *k*_*i*_ returns to a narrow distribution centred around 1.0. These statistical results further corroborate the intimate relation between reshaping of the membrane-bound DNA nanorafts and reshaping of GUVs. In particular, it showcases that it is possible to couple the programmable and reversible operation of the DNA nanorafts to GUVs, equipping microscopic materials with nanotechnology-enabled functionality.

Taking a step further, we set out to investigate the key parameters that can markedly influence the interaction between the DNA rafts and GUVs, including the surface density *σ* of the DNA rafts, osmotic pressure, and the cholesterol-anchor pattern and number on the DNA origami. At a low *σ* ≈ 36 µm^−2^ (Supplementary Fig. 26(i)), neither the conformation state of the DNA rafts nor osmolarity notably affects the GUV morphology (Supplementary Table 1). By increasing *σ* to ~60 µm^−2^ (Supplementary Fig. 26(ii)), substantial differences are resolved among the three states (Supplementary Table 2). With further increases to ~83 µm^−2^ and ~100 µm^−2^ (Supplementary Fig. 26(iii),(iv)), *σ* starts to play a dominating role (Supplementary Tables 3 and 4). The decreased diffusion coefficients at high *σ* also supported these results (Supplementary Fig. 27). In the following, unless specifically mentioned, *σ* ≈ 60 µm^−2^ and an iso-osmotic condition are applied because these give rise to the most distinct behaviour among the different states.

As cholesterol anchors link DNA rafts to the GUV membrane, their number and pattern significantly influence their interaction with GUVs. Free, non-patterned cholesterol-DNA strands fail to induce membrane deformations (Supplementary Figs. 28 and 29). Leveraging the programmability of DNA origami, we design seven s-DR variants with 4 (4 Chol), 8 (8 Chol), 12 (12a–12d Chol) and 16 cholesterol (16 Chol) sites (Fig. 2b and Supplementary Fig. 30). The cholesterol pattern transformations along with the reconfiguration of the DNA rafts are illustrated in Fig. 2b. With 4 (one line) or 8 (two lines) cholesterol sites, no substantial differences among the three states are visible. Fewer cholesterol anchors correspond to weaker membrane affinity of the DNA rafts and thus it is more difficult to offset the energy expense for membrane remodelling. With 12 sites (three lines, that is, 12d in Fig. 2a), membrane affinity increases, enhancing leaflet asymmetry and promoting GUV deformations. With 16 sites (four lines), GUV deformations are observed even at the s-DR state due to strong interactions (Supplementary Fig. 31a). Among the 12 Chol variants, 12d has the narrowest SD_*k*_ distribution at the s-DR state and generates the highest GUV deformations after reconfiguration to e-DRs (Fig. 2b and Supplementary Fig. 31). This can be attributed to the fact that the cholesterol sites are tightly spaced and arranged in parallel lines, introducing strong anchoring of the DNA rafts to the membrane. Meanwhile, these lines are widely expanded from the middle to the two ends, which help to establish the elongated shape of the DNA raft. As a result, 12d, which is associated with the most distinct cholesterol pattern alteration from the s-DR to e-DR state, gives rise to the highest degree of GUV remodelling.

## Formation of synthetic channels by self-arranged DNA rafts

**Fig. 3:**
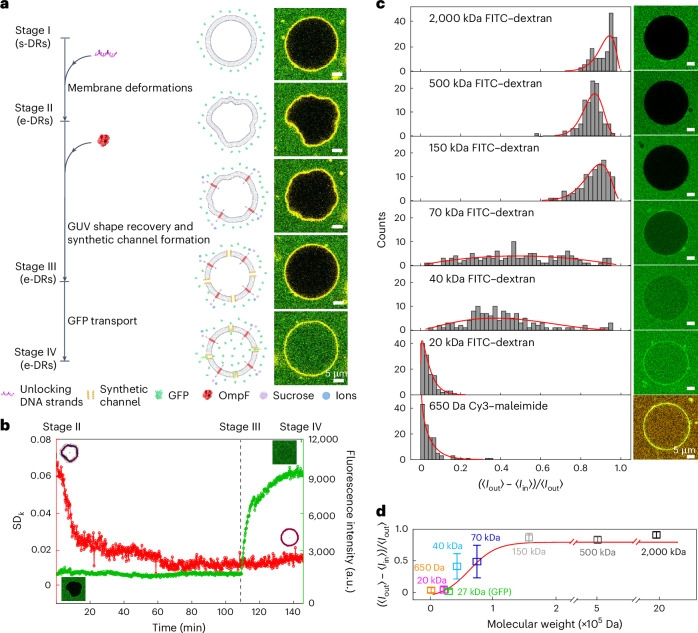
Formation of synthetic channels by self-arranged DNA raft architectures. **a**, Time course of the GFP influx in the hybrid DNA raft–OmpF–GUV system. **b**, SD_*k*_ of the GUV trace (red curve) and fluorescence intensity within the GUV (green curve) recorded over time. **c**, Statistics of the normalized fluorescence intensity difference (〈*I*_out_〉 – 〈*I*_in_〉)/〈*I*_out_〉 of the GUVs at equilibrium using Cy3 and FITC–dextran of different molecular weights as optical probes. **d**, Normalized fluorescence difference as a function of the cargo molecular weight. Data represent mean ± s.e.m. from three independent experiments. *n* for Cy3, 20 kDa dextran, GFP, 40 kDa dextran, 70 kDa dextran, 150 kDa dextran, 500 kDa dextran and 2,000 kDa dextran are 115, 120, 103, 126, 127, 109, 109 and 120, respectively. Source data

To build a cell model comprising hybrid modules, biorelevant elements are subsequently integrated in our DNA raft–GUV system. OmpF^45^, a bacterial outer membrane protein, is chosen because of its stability and facile reconstitution onto lipid membranes. It has a pore size of ∼1.1 nm, allowing the passage of molecules <600 Da. In addition, green fluorescent protein (GFP, ∼27 kDa) is utilized as optical probe for the detection of influx and efflux across the membrane by confocal fluorescence microscopy. At stage I (Fig. 3a), s-DR-bound GUVs are introduced in a GFP (∼500 nM) containing solution. At equilibrium, the GUV is spherically shaped and no GFP transport across the membrane is visible (Fig. 3a). Upon the addition of unlocking strands, the expected GUV reshaping emerges, while the interior of the GUV remains dark (stage II). Subsequently, OmpF (∼300 nM) is reconstituted onto the membrane (Supplementary Fig. 32). Gradual recovery of the GUV from a deformed to a spherical shape is clearly observable, while no GFP influx is detectable (stage III). Here, the molecular cut-off of OmpF has a crucial impact. On the one hand, OmpF does not allow the influx transport of GFP due to size exclusion (Supplementary Fig. 33). On the other hand, OmpF can mediate the exchange of small solutes (sucrose, Na^+^, Mg^2+^, Cl^−^, etc.) to restore osmotic balance across the membrane. The solute exchange and the corresponding matching of their concentrations increase the critical threshold (Supplementary Fig. 17), and the spherical shape can become stable even in the presence of the e-DRs. The free energy required to restore the spherical shape thus is supplied by the entropy that is gained by the molecular solutes. During this small solute-exchange process towards equilibrium, the GUV gradually recovers to a spherical shape. Of note, the detergent of octyl-POE in OmpF has no effect on the membrane recovery process (Supplementary Fig. 34). Interestingly, after a full shape recovery, the transport of GFP into the GUV starts. After ∼30 min, a homogeneous distribution of GFP within the GUV lumen is reached (stage IV). Statistics of the normalized local curvature *k*_*i*_ and the normalized fluorescence intensity difference (〈*I*_out_〉 – 〈*I*_in_〉)/〈*I*_out_〉 of the GUVs at the characteristic stages I–IV are presented in Supplementary Fig. 35. The observed phenomena, namely, membrane deformations, GUV shape recovery, synthetic channel formation and cargo transport across the membranes, are not greatly influenced by the GUV size (Supplementary Fig. 36).

In view of the observed peculiar phenomenon, several control experiments are carried out. First, the post-e-DRs are directly bound to the GUV membrane together with OmpF. No GFP influx is perceived (Supplementary Fig. 37). Second, the s-DRs with a high *σ* ≈ 100 µm^−2^ that can directly induce GUV deformations together with OmpF are bound to the GUV membrane. Nevertheless, after the shape recovery of the GUV, there is still no GFP influx visible (Supplementary Fig. 38). Third, in the combined system of dynamically transformed e-DRs and OmpF bound to SLBs, no synthetic channels are visible by AFM (Supplementary Fig. 39) or detectable by single-channel current recordings (Supplementary Fig. 40). These control experiments elucidate that both the reshaping of the DNA nanorafts, that is, dynamic reconfiguration from the s-DR to the e-DR state, and the reshaping of the GUV, that is, dynamic spherical recovery, which constitutes a genuine 3D membrane effect, are crucial for the formation of the synthetic channels (Supplementary Table 5).

To decipher the interplay between GUV remodelling and the onset of GFP influx, SD_*k*_ (red curve) and the fluorescence intensity within the GUV (green curve) are recorded over time. As shown in Fig. 3b and Supplementary Video 5, the GFP influx occurs only after a full spherical shape recovery of the GUV, which takes around 100 min. Because OmpF does not allow GFP influx due to size exclusion, other membrane channels with dimensions much larger than the OmpF pore must have been formed during the dynamic reconfiguration from the s-DRs to e-DRs, followed by the GUV recovery. As the influx process persists over 40 min, the formed membrane channels cannot be transient in nature. These channels likely come into existence due to the rearrangement of the e-DRs, which constitute locally ordered architectures in domains to induce GUV deformations. Subsequently, during the shape recovery of the GUV resulting from small solute exchange through OmpF, strong fluctuations of the membrane curvatures mediate the membrane perforation by the e-DR architectures, probably from the e-DR ends given the structure’s high aspect ratio. It is likely that the e-DRs are prone to align their hydrophilic surfaces side by side on the membrane in local order, while their partially inserted hydrophobic surfaces shield most of the lipid tails from the hydration of the channel. This eventually leads to the formation of synthetic transmembrane channels by the self-arranged DNA rafts (Supplementary Fig. 41).

To examine the dimension of the synthetic channels, fluorescein isothiocyanate (FITC)–dextran molecules of various molecular weights are used as influx probes^30^. As shown in Fig. 3c, the population peaks for Cy3-maleimide (~650 Da) and 20 kDa FITC–dextran are sharply located around 0, indicating nearly 100% transport. Larger molecules (40 and 70 kDa) show broader distributions. For 70 kDa dextran, the centre of the population distribution shifts to 50%, indicative of partial permeability. For 150 kDa and 500 kDa FITC–dextran, the population distributions become narrow again and the population peaks are positioned above 85%. For 2,000 kDa FITC–dextran, the membrane is almost non-permeable. The normalized fluorescence difference as a function of the cargo molecular weight is presented in Fig. 3d. The estimated dimension of the synthetic channels is in the range of 15 nm, judging from the hydrodynamic diameter of 70 kDa FITC–dextran^46^. In addition, the synthetic channels can release molecular cargo across the membrane as well (Supplementary Fig. 42).

## Reversible sealing of the synthetic channels and gated cargo transport

**Fig. 4:**
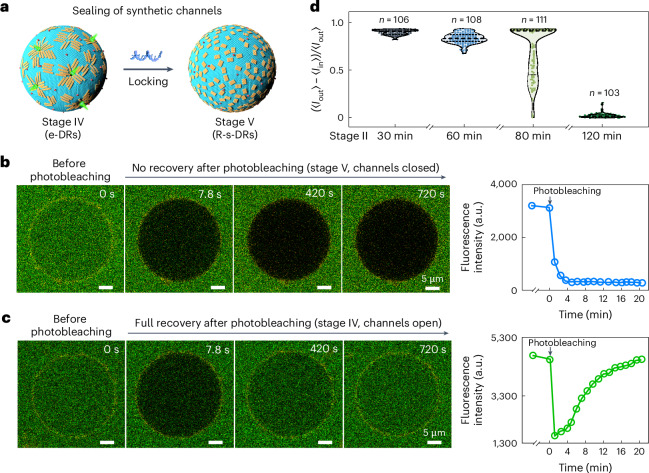
Reversible sealing of the synthetic channels and gated cargo transport. **a**, Schematic of sealing of the synthetic channels through the transformation of e-DRs (stage IV) to R-s-DRs (stage V) by adding locking strands. **b**, Closure of the synthetic channels by adding locking strands at stage V. Photobleaching of GFP within the GUV using FRAP shows no fluorescence signal recovery. **c**, In the absence of locking strands, photobleaching of GFP within the GUV using FRAP shows a rapid signal recovery. **d**, Violin plots of the GFP influx resulting from intervention of the synthetic channel formation by adding locking strands at different time intervals from stage II. Data obtained from three independent experiments (*n* = 102, 108, 111 and 103 for 30 min, 60 min, 80 min and 120 min time points, respectively). Source data

Subsequently, we investigate whether the synthetic channels can be reversibly sealed and gated on demand. Locking strands are added at stage IV to trigger the transformation from e-DRs to R-s-DRs on the GUV membrane (Fig. 4a). At equilibrium (stage V), photobleaching of GFP within the GUV using FRAP is carried out. An immediate fluorescence intensity drop occurs in the GUV lumen and no signal recovery is observed over time (Fig. 4b and Supplementary Video 6). This demonstrates the successful closure of the synthetic channels. Upon the addition of locking strands, the locally ordered e-DRs are transformed to disordered R-s-DRs. This lifts the membrane perforation imposed by the e-DRs, so that no GFP influx across the membrane is permissible. Conversely, in the absence of locking strands, the fluorescence intensity in the GUV lumen can rapidly recover after FRAP due to the subsequent GFP influx through the synthetic channels (Fig. 4c). In a control experiment, in which GFP is only added after stage V, due to the closure of the synthetic channels, no prominent GFP influx is observed (Supplementary Fig. 43).

As stages II, III and IV span a time period of over 2 h (Fig. 3b), locking strands are added at selected time intervals after the completion of stage II to intervene in the perforation process (Fig. 4d and Supplementary Fig. 44). The addition of locking strands after 30 min from stage II results in a very low GFP percentage influx. The e-DRs transform to R-s-DRs, so that the locally ordered state is disturbed and GUV perforation is inhibited. As a result, due to the lack of synthetic channels, the GFP influx is not effectively supported. By adding locking strands at 60 min or 80 min, the GFP transport across the membrane becomes more noticeable. In particular, for the addition at 80 min, the broad spread of the percentage influx distribution reveals that the addition of locking strands even at such a late stage still has a substantial impact on the formation of the synthetic channels. By adding locking strands at 120 min, when the GFP transport has nearly reached equilibrium, the closure of the synthetic channels has minimal influence and thus (〈*I*_out_〉 – 〈*I*_in_〉)/〈*I*_out_〉 is approximately 0. This set of experiments confirms that the synthetic channels can be engineered to open and close on demand.

## Enzyme cascade reactions within synthetic cells

**Fig. 5:**
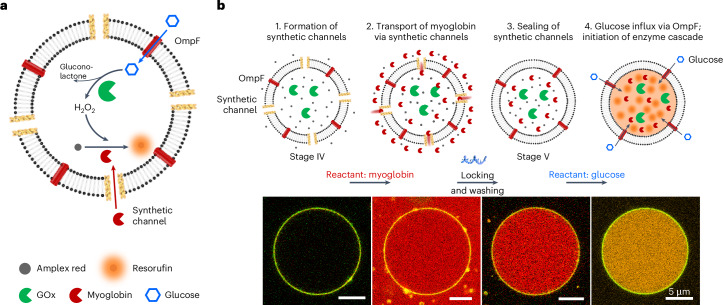
Enzyme cascade reactions within synthetic cells. **a**, Pathway of the GOx–myoglobin enzyme cascade. The GOx-catalysed reaction of glucose and oxygen into gluconolactone and H_2_O_2_ is followed by a peroxidase reaction catalysed by myoglobin (red). This enzyme cascade produces resorufin (orange) within the GUV. **b**, Intermediate schematics and experimental data of the enzyme cascade: e-DRs (Atto488, green), myoglobin (Cy5, red), OmpF (Cy5, red) and resorufin (orange).

In confined enzyme cascade reaction experiments, reactants of high molecular weights are often either directly pre-encapsulated during the production of GUVs or injected into vesicles^47–49^. Here, taking advantage of the distinct sizes and functions of our synthetic channels and OmpF, different reactants are transported into GUVs step by step, enabling high spatiotemporal control of the enzyme cascade reactions within GUVs. As shown in Fig. 5b(1), at stage IV, synthetic channels are formed in the membrane of the GUV encapsulated with glucose oxidase (GOx) (160 kDa). The substrate Amplex red is then added, which diffuses passively across the GUV membrane. Cy5-labelled myoglobin (Supplementary Fig. 45) is then introduced to the exterior space as reactant. The opening of the synthetic channels allows for the transport of myoglobin (∼17 kDa) into the GUV, which can be visualized by the homogeneous distribution of myoglobin in the GUV lumen (Fig. 5b(2)). Locking DNA strands trigger the transition from e-DRs to R-s-DRs on the GUV (stage V), sealing synthetic channels to encapsulate myoglobin (Fig. 5b(3) and Supplementary Fig. 46a) and preventing its diffusion during the washing step, which aims to remove residual myoglobin from the exterior space. Subsequently, the next reactant, glucose (180 Da), diffuses into the GUV via OmpF. The glucose influx immediately triggers the GOx–myoglobin enzyme cascade, as evidenced by the production of fluorescent resorufin (appearing orange) within the GUV (Fig. 5b(4) and Supplementary Fig. 46b,c).

It is worth mentioning that our finding is a generic effect that does not hinge on the specific OmpF. We performed an experiment equivalent to that in Fig. 3a by replacing OmpF with the protein translocase of the outer membrane (TOM; pore size, ∼2.5 nm; ∼6 kDa cut-off) and replacing GFP with mitochondrial preproteins (∼40 kDa) (Supplementary Fig. 47). Translocases are of great importance in cell biology. Most of the proteins needed for mitochondrial metabolism, growth, division and partitioning to daughter cells are encoded by the nucleus of the cell. To sustain these essential functions, TOM works in conjunction with various translocases located in the inner mitochondrial membrane to transport proteins into the mitochondrion^50,51^. Supplementary Fig. 47b reveals that the DNA raft–TOM–GUV system also exhibits the same series of phenomena, namely, membrane deformations, GUV shape recovery, synthetic channel formation and cargo transport across the membranes. Crucially, the translocation of the preproteins is not effective in the case of GUVs reconstituted with TOM alone (Supplementary Fig. 47c). In contrast, the mitochondrial preproteins can be directly transported into DNA raft–TOM–GUVs by creating synthetic channels, without the need to be threaded through the nanoscale pores of the TOM complex or undergo substrate specific protein translocases.

## Discussion

In living cells, the shapes of biological matter are central to the cell function at different length scales, from the molecular to the microscopic level^52,53^. Nevertheless, the shapes of cellular components are often transient and hard to reprogramme. DNA nanotechnology, which offers unrivalled spatiotemporal control over nanostructures and biochemical functions, could provide a framework to couple programmability and addressability to cells at the microscale. In this work, we have exemplified a relationship between reshaping of signal-responsive DNA nanorafts and reshaping of GUVs. The deformations of GUVs are a consequence of the work imposed by the DNA rafts. In turn, the shape recovery of GUVs mediated by OmpF assists the DNA rafts to form locally ordered architectures for membrane perforation. The resulting membrane-spanning channels enable efficient cargo influx and efflux. Importantly, these channels can also be programmably sealed and gated on demand.

The high sequence specificity of DNA has resulted in origami reconfiguration, membrane anchor placements and self-assembling patterns being engineered to explore the intriguing interplay between the reshaping of DNA rafts and GUVs. Investigating such key parameters is instructive to gain insights into how membrane proteins and cells interact and how they have evolved to their present biological functions. This concept will expand the realm of synthetic biology to establish a suite of new design principles and models that can be efficiently re-engineered according to necessity.

Furthermore, the synthetic channels formed by the DNA rafts with controlled permeability may be interesting for both fundamental research and biomedical applications. In living cells, the entry and exit of large molecules across membranes is mostly regulated by protein pores, which are often fragile to engineer and only possess a narrow size range up to 5 nm. Going beyond this size limit and overcoming some restrictions of protein pores are of profound biological and technological significance. To place our finding in a more general context, it may become possible to directly shuttle or sense large protein complexes and enzymes across membranes without the need to go through protein pores or undergo intricate and cooperative biological processes via substrate-specific protein translocases^54^. Instead, the formed synthetic channels could facilitate the direct transport of large bioactive substances. Also, given the modularity of DNA origami, the DNA rafts could be functionalized with diverse moieties to accurately recognize and programmably puncture diseased cells to activate apoptosis or control the release of synthetic cell-enclosed therapeutics or cytotoxic drugs. We envisage that the future work along these directions could stimulate new solutions to unmet bottlenecks in cellular delivery and release of large biological entities, such as protein complexes, small synthetic vesicles, therapeutics and personalized medicine, or cytotoxic drugs.

## Methods

### Assembly and purification of the s-DRs

The s-DR was designed using Cadnano. The detailed sequences are presented in Supplementary Table 6. The s-DRs were assembled as follows. A long scaffold strand (∼7560 nt circular single-stranded M13mp18 DNA, 20 nM) was folded into a square shape via interactions with 154 staple strands (100 nM of each strand; Supplementary Table 6) in a 1 × TE buffer, supplemented with 1 mM EDTA and 12 mM MgCl_2_. The samples were then annealed for 10 h following a thermal annealing protocol: 95 °C for 5 min, then from 85 to 24 °C at a rate of 10 min °C^−1^. The s-DRs were purified by polyethylene glycol (PEG) precipitation for all microscopy experiments. The folded s-DR sample was mixed at a 1:1 ratio with the PEG buffer (15% PEG 8000 (w/v), 5 mM Tris, 1 mM EDTA and 505 mM NaCl) and incubated at 4 °C for 30 min. The solution was centrifuged at 17,000*g* for 30 min at room temperature. The supernatant was removed using a pipette. The remaining pellet was then dissolved in the imaging buffer (92.5 mM NaCl, 5 mM MgCl_2_, 20 mM HEPES, pH 7.2, ∼220 mOsm kg^−1^).

### Reversible conformation changes of the DNA rafts in solution

The transformation mechanisms among s-DRs, e-DRs and R-s-DRs by adding DNA strands are shown in Supplementary Fig. 1a. The corresponding AFM images are presented in Supplementary Fig. 1b–d. The s-DRs were incubated with 22 unlocking strands (Supplementary Fig. 1 and Supplementary Table 7) at 40 °C for 1 h to yield the e-DRs. Incubating the e-DRs with 22 locking DNA strands (Supplementary Fig. 1 and Supplementary Table 8) at 40°C for 1 h transformed them to R-s-DRs.

### Reversible conformation changes of the DNA rafts on SLBs

SLBs were formed at 60 °C via fusion of small unilamellar vesicles (SUVs) deposited in the imaging buffer (92.5 mM NaCl, 5 mM MgCl_2_ and 20 mM HEPES, pH 7.2) on top of freshly cleaved mica. The preparation of SUVs was as follows: DOPC (Avanti Polar Lipids) was dissolved at a concentration of 25 mg ml^−1^ in chloroform for SLB formation. Then, 40 µl DOPC stock solution was transferred to a clean glass vial and dried under a stream of nitrogen for 10 min and then overnight under vacuum to remove the residual organic solvent. The dried lipids were then suspended in 1 ml of double-deionized water and sonicated for 6 min using a Branson Ultrasonics Sonifier (60% duty cycle) to obtain SUVs with a lipid concentration of 1 mg ml^−1^. After a 30 min waiting period, the mica was gently washed with 400 µl of the imaging buffer to remove the excess lipids and the SLB was slowly cooled down to room temperature to reduce the occurrence of membrane defects. Then, s-DRs with specific cholesterol anchors (Supplementary Table 9) were prepared and purified by PEG precipitation. Subsequently, the purified s-DRs were incubated with the prepared SLB for 40 min. The insertion of the cholesterol anchors into the leaflet of the bilayer ensured the successful attachment of the s-DRs onto the membrane (Fig. 1c and Supplementary Fig. 9a). The unbound s-DRs were removed by gently washing the sample with 400 µl of the imaging buffer twice. The conformation change of s-DRs to e-DRs was triggered by adding unlocking strands at 40 °C after incubation for 1 h (the temperature was controlled by using a heating plate). As a result, the s-DRs were dynamically transformed to the e-DRs on the membrane (Fig. 1c and Supplementary Fig. 9b). Addition of locking strands to the sample at 40 °C and incubation for 1 h reversibly reconfigured the e-DRs to R-s-DRs (Fig. 1c and Supplementary Fig. 9c).

### AFM imaging

For AFM imaging, the samples were prepared by deposition of the DNA nanostructures (2 µl) onto freshly cleaved mica. Then, a 100 µl 1 × TE buffer with 1 mM EDTA and 5 mM MgCl_2_ was added onto the mica and the samples were imaged. AFM was performed using a Multimode VIII with a Nanoscope V controller and Icon (Bruker). The AFM tip was a microcantilever SNL-10 (Bruker). For AFM imaging on the SLB interface, an imaging buffer (92.5 mM NaCl, 5 mM MgCl_2_ and 20 mM HEPES, pH 7.2) was used.

### Preparation of GUVs

GUVs were prepared by electroformation in Teflon chambers with platinum electrodes. A lipid mixture (4 µl, 2 mg ml^−1^ in hexane) was spread onto two platinum wires and dried under vacuum for 2 h. An a.c. electric field of 2.2 V (peak-to-peak voltage, sine wave shape) was applied at a frequency of 10 Hz for 4 h, followed by 1 Hz for 0.5 h to detach GUVs from the platinum wires into the solution. Unless otherwise stated, vesicles composed of DOPC, containing an additional 0.05 mol% (for confocal imaging) Atto655-DOPE or 0.005 mol% (for fluorescence correlation spectroscopy (FCS)), were electroformed in an aqueous solution of sucrose iso-osmolar with respect to the imaging buffer (∼220 mOsm kg^−1^). The obtained GUVs had a diameter range ∼10–40 µm (Supplementary Fig. 11).

### Reversible conformation changes of the DNA rafts on GUVs

FRET was used to characterize the reversible conformation changes of the DNA rafts on GUVs. Cy3 and Cy5 were positioned on the DNA origami (Supplementary Table 10). In the s-DR state, the distance between Cy3 and Cy5 was ∼7.2 nm, whereas in the e-DR state this distance was shortened to ∼3.1 nm. After 1 h incubation of the 1.1 nM s-DRs (surface density *σ* ≈ 60 µm^−2^) with the preadsorbed GUVs on a glass slide in the imaging buffer (∼220 mOsm kg^−1^), the sample was illuminated by the 561 nm line of an argon ion laser. Then, incubating unlocking strands with the sample at 40 °C to trigger the conformation change from s-DRs to e-DRs, the sample was excited by the laser again. Finally, locking strands were added to trigger the reversible conformation change from e-DRs to R-s-DRs at 40 °C and the FRET measurements were carried out.

### Laser-scanning confocal fluorescence microscopy

Confocal imaging was performed using LSM 710 and LSM 980 (Zeiss) laser scanning microscopes with an oil immersion objective (C-Apochromat, 60×/1.2 W ultraviolet–visible–infrared, Zeiss). Unless otherwise stated, the samples were illuminated by the 488 nm line of the argon ion laser (for Atto488 excitation) or with the 633 nm line of a helium neon laser (for Atto655 excitation) by using the LSM 710. Further image analysis was performed by a MATLAB program.

### Reshaping of GUVs by reshaping of the DNA rafts

The DNA rafts were modified with eight Atto488 dyes (Supplementary Table 10). The s-DRs (1.1 nM) were mixed with GUVs at an s-DR/lipid molar ratio of 1:1,000 (*σ* ≈ 60 µm^−2^). The mixture was incubated for 1 h to allow for membrane binding. Subsequently, unlocking strands were added to trigger the conformation change from s-DRs to e-DRs with an incubation time of 1 h. The GUVs were deformed. Then, locking strands were added to reconfigure e-DRs to R-s-DRs for the shape recovery of the GUVs. The entire process was carried out at 40 °C, while keeping the osmolarity nearly constant.

### Fluorescence correlation spectroscopy

FCS measurements were carried out according to ref. ^17^ using an LSM 980 confocal microscope (Zeiss). In brief, a 488 nm laser for Atto488 excitation was used at a low laser power (<1.2 μW) to avoid photobleaching and fluorescence saturation. The radius of the waist of the FCS detection volume, *r*_0_ (207 ± 7 nm), was calibrated using a fluorescent dye (Atto488) with a known diffusion coefficient in water (*D*(Atto488) = 414 μm^2^ s^−1^ at 25.0 ± 0.5 °C) and corrected for the working temperature at the objective (27.5 ± 1.0 °C). FCS on membranes was performed at the upper pole of a GUV with a diameter of at least 20 μm. The particle number *N* and consequently the surface density *σ* of the s-DRs on the GUV were obtained from analysis of the autocorrelation functions. A one-component 2D diffusion model was used (equation (1)) to analyse the obtained correlation curves for calculations.

1 $$G\left(\tau \right)=\frac{1}{N}\frac{1}{1+\frac{\tau }{{\tau }_{\mathrm{D}}}}$$

Here, *N* is the number of particles in the 2D detection volume and *τ*_D_ is the FCS diffusion time, which is determined by the translational diffusion coefficient *D* and the size of the 2D Gaussian detection volume as follows: ${\tau }_{\mathrm{D}}={r}_{0}^{2}/\left(4D\right)$. Knowing the length (*L* = 70 nm) and width (*W* = 55 nm) of the s-DR, the surface density *σ* ($\sigma =N/(\uppi {r}_{0}^{2})$ expressed in particles per µm^2^) can be converted to the surface coverage *φ* = *σLW*. The parameters for the determination of surface density and surface coverage of s-DRs on GUVs can be found in Supplementary Tables 11–14.

### Isolation of OmpF

Native OmpF protein was purified from *Escherichia coli* strain BE BL21(DE3) omp6 lacking both LamB and OmpC. Approximately 5 g of cells from 1 litre of culture were suspended in 20 ml of lysis buffer containing 2 mM MgCl_2_ and ∼750 units of DNase and 50 mM Tris–HCl, pH 7.5. Cell membranes were then broken by passing the cells three times through a precooled (4 °C) French press at 1,000 psig. Unbroken cells were removed by centrifugation at 4,000*g* for 15 min at 4 °C. Then the supernatant was centrifuged at 100,000*g* for 1 h at 4 °C to collect the membranes. The membrane pellet was resuspended in 10 ml of 50 mM Tris–HCl, pH 7.5 using a ball-bearing glass homogenizer and then mixed with an equal volume of 4% (w/v) sodium dodecyl sulfate (SDS), 2 mM β-mercaptoethanol and 50 mM Tris–HCl, pH 7.5. After incubation for 30 min at 50 °C, the solution was centrifuged at 100,000*g* for 1 h at 20 °C. The pellet was resuspended in 2% (w/v) SDS, 0.5 M NaCl and 50 mM Tris–HCl, pH 7.5 using a ball-bearing glass homogenizer. It was then incubated at 37 °C for 30 min and centrifuged again at 100,000*g* for 30 min at 20 °C. OmpF was recovered from the supernatant. Finally, the supernatant containing OmpF was mixed with 0.5% (w/v) *n*-octyl polyoxyethylene (octyl-POE, Bachem) and dialysed twice against dialysis buffer (20 mM Tris, pH 8, 1 mM EDTA and 1% (w/v) octyl-POE) overnight at 4 °C to remove SDS using dialysis tubing with a cut-off of 20 kDa. The purity of the isolated proteins (∼1.0 mg ml^−1^) was determined by SDS–PAGE. The protein samples were frozen in liquid nitrogen and stored at −20 °C until further use.

### Isolation of TOM

TOM core complex containing a 6 × His tag at subunit Tom22 was isolated as follows. Briefly, 2 g of *Neurospora crassa* mitochondria was solubilized in 200 ml solubilization buffer (1% *n*-dodecyl-β-d-maltoside (DDM), 20% glycerol, 300 mM NaCl, 20 mM imidazole–HCl, 20 mM Tris–HCl, pH 8.5, 1 mM phenylmethylsulfonyl fluoride (PMSF)) at a protein concentration of 10 mg ml^−1^. Non-solubilized membranes were separated from the solubilized membrane proteins by ultracentrifugation and filtration through standard-grade filter paper. A prepacked 5 ml Ni-NTA column (Cytiva) was equilibrated with equilibration buffer (0.1% DDM, 10 % glycerol, 300 mM NaCl, 20 mM Tris–HCl, pH 8.5, 1 mM PMSF) by using an automated protein purification system (Äkta, Cytiva). The solubilized protein sample was loaded onto the Ni-NTA column. The column was rinsed with equilibration buffer to remove unbound proteins. The TOM core complex was eluted with 30% TOM elution buffer (0.1% DDM, 10% glycerol, 20 mM Tris–HCl, pH 8.5, 1 mM PMSF, 300 mM imidazole). For further purification, a prepacked Resource Q anion-exchange column (1 ml, Cytiva) was equilibrated with ResQ buffer 1 (20 mM HEPES, pH 7.2, 2% dimethylsulfoxide (DMSO), 0.1% DDM). The Ni-NTA column peak fraction containing TOM was loaded onto the anion-exchange column. Unbound proteins were removed by rinsing the column with ResQ buffer 1 and a linear salt gradient of 0–22% ResQ buffer 2 (20 mM HEPES, pH 7.2, 2% DMSO, 0.1% DDM, 1 M KCl). Eventually, TOM was eluted with a linear salt gradient of 22–40% ResQ buffer2. The purity of TOM samples (0.4–1.2 mg ml^−1^) was assessed by SDS–PAGE followed by staining with Coomassie Brilliant Blue. Protein samples were flash frozen in liquid nitrogen and stored at -20 °C until further use.

### Fluorescent labelling of the OmpF, TOM, Su9-MBP and myoglobin proteins

OmpF (1 ml, 1 mg ml^−1^) was covalently labelled with Atto647N NHS ester (AAT Bioquest) at a 1:5 molar ratio of protein to dye in 20 mM Tris, pH 8, 1 mM EDTA and 1% (w/v) octyl-POE for 2 h at room temperature in the dark in a nitrogen gas atmosphere. Unconjugated dyes were removed by loading the sample onto a 1 ml anion-exchange column (HiTrap Q FF, Cytiva) equilibrated with 0.5% (w/v) octyl-POE and 50 mM Tris–HCl, pH 7.5. After washing the column with ten column volumes of equilibration buffer, fluorescently labelled OmpF was eluted with 300 mM KCl, 0.5% (w/v) octyl-POE and 50 mM Tris–HCl, pH 7.5.

Fluorescent labelling of TOM with fluorescent dye Cy3-maleimide was performed as follows. First, 500 μl Ni-NTA resin (Thermo Fisher) was washed with double-deionized water in a gravity flow column (Bio-Rad) and then equilibrated with ∼5 column volumes FL buffer (20 mM HEPES, 2% DMSO, 350 mM KCl, 0.1% DDM). Approximately 1 mg of purified TOM (1 mg ml^−1^) was loaded onto the equilibrated 500 μl Ni-NTA resin and washed with 8 column volumes of FL buffer at 4 °C. FL buffer containing 0.1 mM tris(2-carboxyethyl)phosphine was added to the column, incubated for 10 min at room temperature and washed with 20 column volumes of FL buffer at 4 °C. Next, the mixture of TOM and Ni-NTA resin was removed from the column and transferred to an Eppendorf tube. Cy3-maleimide in FL buffer was added to the TOM bound to the Ni-NTA resin at a 1:5 molar ratio of complex to dye. To avoid oxidation, the top of the Ni-NTA resin was covered with nitrogen gas. After 2 h of reaction labelling at room temperature in the dark, Cy3-labelled TOM molecules were separated from unconjugated Cy3-maleimide dye by washing the Ni-NTA resin five times with 4 column volumes of FL buffer. Finally, the Cy3-labelled TOM core complex molecules were eluted with 1 column volume of FL buffer containing 300 mM imidazole.

Mitochondrial presequence protein (Su9-MBP, courtesy of P. Ornelas, MPI Biophysics) was covalently labelled with the fluorescent dye Cy5-maleimide as follows. The mitochondrial presequence protein (1 mg ml^−1^) was treated with 0.1 mM tris(2-carboxyethyl)phosphine for 10 min at room temperature. Then, mitochondrial presequence protein was reacted with Cy5-maleimide in degassed 20 mM HEPES, pH 7.2, and 50 mM KCl at a 1:5 molar ratio of complex to dye for 2 h in the dark. To avoid oxidation, the reaction was carried out under a nitrogen atmosphere. Unconjugated dyes were removed from the Cy5-labelled protein using a PD-10 desalting column (GE Healthcare).

The purities of fluorescently labelled proteins were assessed by SDS–PAGE, first visualized by 555 nm and 638 nm light and then by Coomassie Brilliant Blue staining. Fluorescently labelled protein samples were flash frozen in liquid nitrogen and stored at −20 °C until further use.

Myoglobin (1 ml, 100 µM) was labelled at the N-terminus with Cy5 NHS ester at a 1:5 molar ratio of protein to dye in HEPES (20 mM, pH 8.3) for 2 h at room temperature in the dark. Unbound dyes were removed by using a PD-10 desalting column (GE Healthcare). The purified Cy5-labelled myoglobin was assessed by SDS–PAGE and ultraviolet–visible absorbance spectra.

### GOx–myoglobin enzyme cascade in the DNA raft–OmpF–GUV system

First, 0.05 mg ml^−1^ GOx (160 kDa) was encapsulated in GUVs by electroformation. GOx-containing GUVs were washed three times with isotonic sucrose to remove external GOx. At stage IV, synthetic channels were formed in the membrane of the GUV encapsulated with GOx. Substrate Amplex Red (5 μM) was added, followed by its passive diffusion across the GUV membrane. Cy5 fluorescently labelled myoglobin (2 μM) was then introduced and transported into the GUVs as reactant. After equilibrium, locking DNA strands were added to the system to trigger the transition from e-DRs to s-DRs on GUVs to sealing the synthetic channels at 37 °C for 2 h, thereby driving the system to stage V. Then the samples were washed gently to remove myoglobin residuals in the exterior space by using imaging buffer. Subsequently, another reactant, glucose (1 mM), was added and transported into the GUV via OmpF to initiate the cascade reaction. Production of resorufin in the GUVs was visualized by confocal fluorescence microscopy.

### Data analysis

#### Shape tracing of the equatorial confocal GUV slices

For the analysis of remodelling of GUVs, equatorial confocal GUV membrane slices (Supplementary Video 4, green dots) were traced using a customized, fully automated analysis routine implemented in MATLAB (Mathworks) from raw GUV images (512 × 512 pixels). For this purpose, the centre of mass of a GUV membrane slice **x**_0_ = (*x*_0_,*y*_0_) and an initial pixel *x*_*i*_ on the membrane were first selected manually. **x**_0_ was defined as the origin. Then, a defined region of interest (ROI, typically 10–30 pixels) was centred on **x**_*i*_ and a second-order polynomial function

2 $$y\left(x\right)=a+{bx}+c{x}^{2}$$

was fitted through the pixel coordinates {*x*_ROI_} with intensities higher than the average intensity of a rectangular region centred on **x**_0_ with a width of *d* = |**x**_*i*_| + 20 pixels, depending on the GUV size (Supplementary Video 4). Since **x**_*i*_ did not necessarily correspond to the coordinates of a pixel of the ROI, the pixel ${{\mathbf{x}}}_{{{i}}}^{\,{{{\prime} }}}$ of {*x*_ROI_} closest to the vertex of the polynomial function (2) was used for further membrane tracing (Supplementary Video 4, red dots). The new initial coordinate **x**_*i*+1_ was then determined by

3 $${{\mathbf{x}}}_{{{i}}{\boldsymbol{+}}{{1}}}=M\left(\theta \right)\times\mathbf{x}_{i}$$

where *M*(*θ*) is the rotation matrix. By iterative rotations from 0° to 360° in steps of 3°, the shape of the entire membrane representing a GUV slice was traced.

Subsequently, the local curvature *k*_*i*_ of the vesicle trace was determined according to

4 $${k}_{i}=\frac{{R}_{0}}{{R}_{i}}$$

where *R*_0_ is the radius fitted to all ${{\mathbf{x}}}_{{{i}}}^{\,{{{\prime} }}}$ positions along a GUV trace and *R*_i_ is the distance between the centre of this fitted circle and ${{\mathbf{x}}}_{{\boldsymbol{i}}}^{{{\,{\prime} }}}$ (Supplementary Video 4, blue circle). The degree of membrane deformation of a GUV caused by the DNA rafts was defined by the standard deviation SD_*k*_ of all *k*_*i*_. A GUV was considered deformed if the corresponding SD_*k*_ value was greater than the mean SD_*k*_ of *n* = 150 bare GUVs in the absence of DNA rafts, which was 〈SD_*k*_〉_bare_ = 0.026 ± 0.005 (Supplementary Fig. 20). Deformation efficiency was calculated by the ratio between the number of deformed GUVs and the total number of analysed GUVs.

#### Quantification of the DNA rafts binding to GUV membranes

The binding kinetics of the fluorescently labelled DNA rafts to the membrane of a GUV was analysed using a time series of images with MATLAB. The centre of mass of a GUV membrane slice **x**_0_ = (*x*_0_,*y*_0_) and a rectangular ROI centred at **x**_0_ with a width depending on the GUV size was manually determined. The intensities of all pixels higher than the average intensity of the ROI were integrated and plotted as a function of time. The binding of the DNA rafts followed the classical target-binding kinetics

5 $$I={I}_{\max }\left(1-{e}^{-{at}}\right)$$

where *I* is the intensity of origami bound to the membrane, *t* is the time, *I*_max_ is the intensity at the plateau and *a* is the rate constant of binding.

#### Analysis of GFP and FITC–dextran flux into GUVs

To investigate the kinetics of GFP and FITC–dextran flux into GUVs, the central position of a GUV was manually selected and used to define an ROI within the vesicle (∼30 × 30 pixels). The mean fluorescence intensity 〈*I*_in_〉 within the GUV was determined at different time points and compared with the background intensity 〈*I*_out_〉 from two ROIs of ∼50 × 50 pixels each. Relative import levels were calculated by (〈*I*_out_〉 − 〈*I*_in_〉)/〈*I*_out_〉 using MATLAB.

### Reporting summary

Further information on research design is available in the Nature Portfolio Reporting Summary linked to this article.

## Online content

Any methods, additional references, Nature Portfolio reporting summaries, source data, extended data, supplementary information, acknowledgements, peer review information; details of author contributions and competing interests; and statements of data and code availability are available at 10.1038/s41563-024-02075-9.

## Supplementary information

Supplementary Information Supplementary Figs. 1–47, Tables 1–14 and references.

Reporting Summary

Supplementary Video 1 a, GUV deformations induced by the conformation change from the s-DRs to e-DRs after adding unlocking DNA strands. b, Control experiment by adding randomly-sequenced DNA strands.

Supplementary Video 2 a, 3D reconstruction of the s-DR-bound GUV based on the z-stack slices. b, 3D reconstruction of the e-DR-bound GUV based on the z-stack slices.

Supplementary Video 3 a, GUV shape recovery induced by the conformation change from the e-DRs to R-s-DRs after adding locking DNA strands. b, Control experiment by adding randomly-sequenced DNA strands.

Supplementary Video 4 Shape tracing of the GUV slice at the equatorial plane.

Supplementary Video 5 GFP influx in the hybrid DNA raft–OmpF–GUV system.

Supplementary Video 6 a, Sealing of the synthetic channels induced by the conformation change from the e-DRs to R-s-DRs after adding locking strands. b, In the absence of locking strands.

Supplementary Data 1 Supplementary Data Fig. 1–51. Confocal images and the corresponding ki of individual GUVs.

## Source data

Source Data Fig. 1 Statistical source data.

Source Data Fig. 2 Statistical source data.

Source Data Fig. 3 Statistical source data.

Source Data Fig. 4 Statistical source data.

## Data Availability

The data reported in this paper are available in the main text and the Supplementary Information. Any additional data are available from the corresponding authors upon request. Source data are provided with this paper.
