# Supplementary material for: Morphology remodelling and membrane channel formation in synthetic cells via reconfigurable DNA nanorafts
Source: Nat Mater. 2025 Jan 13;24(2):278–86. doi: 10.1038/s41563-024-02075-9 (PMC11790494; doi:10.1038/s41563-024-02075-9)
Supplement: Supplementary file 1 — Supplementary Figs. 1–47, Tables 1–14 and references. [file 41563_2024_2075_MOESM1_ESM.pdf]

nature materials

Supplementary information

https://doi.org/10.1038/s41563-024-02075-9

Morphology remodelling and membrane channel formation in synthetic cells via reconfigurable DNA nanorafts

In the format provided by the authors and unedited

Contents

The PDF file includes:

Materials
Fig. S1 to S47
Tables S1 to S14
References

Other Supplementary Materials for this manuscript include the following:

Supplementary Video 1 to Video 6
Supplementary Data S1 to S51

Materials

All purchased materials were used without further purifications. Single-stranded M13 bacteriophage scaffold strands (p7560) were purchased from Tilibit nanosystem. Chemically synthesized DNA strands (staple strands, sequences shown in Supplementary Tables S6-S10) were purchased from Sigma-Aldrich Inc. in a 1× TE buffer (5mM, pH 8.0) at 100 µM each. Agarose for electrophoresis and SYBR Gold nucleic acid stain were purchased from Life Technologies. Lipids 1,2-dioleoyl-sn-glycero-3-phosphocholine (DOPC, # 850375C) were purchased from Avanti Polar Lipids. Lipids 1,2-Dioleoyl-sn-glycero-3-phosphoethanolamine (DOPE-ATTO655) were purchased from ATTO-TEC GmbH. Glucose oxidase (GOx) was purchased from Sigma-Aldrich. Myoglobin from horse skeletal muscle was purchased from Sigma-Aldrich and was used without further purifications. Cy5-NHS ester (AAT Bioquest) was purchased from Biomol. All other chemicals (*e.g.*, sucrose, HEPES, FITC-dextran, Amplex Red, glucose) were purchased from Sigma-Aldrich Inc.

Results

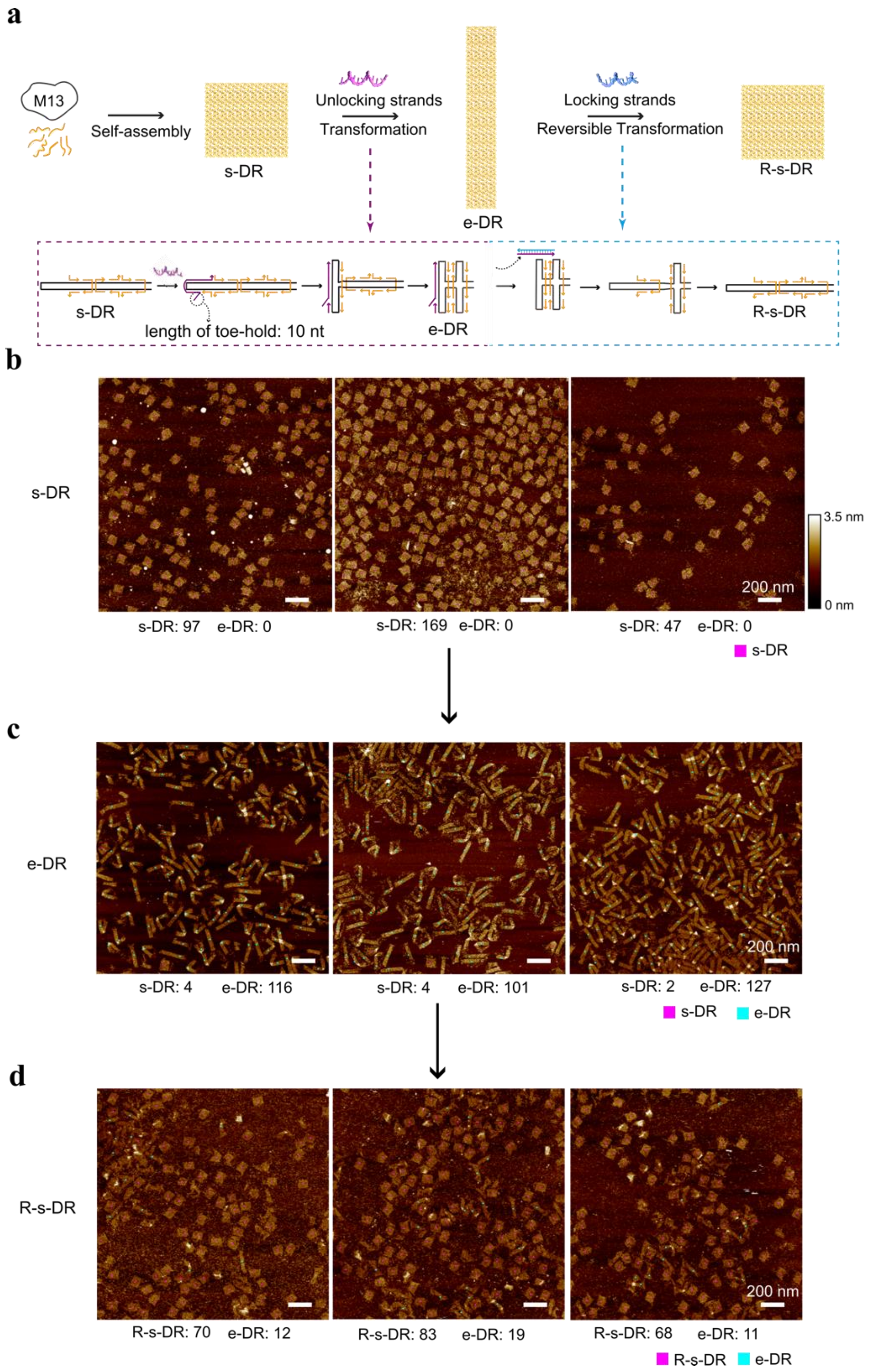

Fig. S1. Conformation changes of the DNA rafts. (**a**) Schematic of the three states of the DNA rafts. The s-DR (70.8 nm × 55 nm, aspect ratio ~1.3) is reconfigured to the e-DR (190 nm × 20 nm, aspect ratio ~9.5) upon the addition of unlocking strands, whereas the reversible process is driven by adding locking strands to R-s-DRs [1]. (**b**) AFM images at the s-DR state. The analyzed s-DRs are indicated using magenta squares. (**c**) AFM images at the e-DR state. The analyzed s-DRs and e-DRs are indicated using magenta and cyan squares, respectively. The transformation efficiency from the s-DRs (2 nM) to e-DRs by the addition of unlocking DNA strands (8 nM) is about 97.1% (N_{total} = 354). (**d**) AFM images at the R-s-DR state. The transformation efficiency from the e-DRs to R-s-DRs by the addition of locking DNA strands (15 nM) is about 84.3% (N_{total} = 263). The analyzed R-s-DRs and e-DRs are indicated using magenta and cyan squares, respectively. Scale bars: 200 nm.

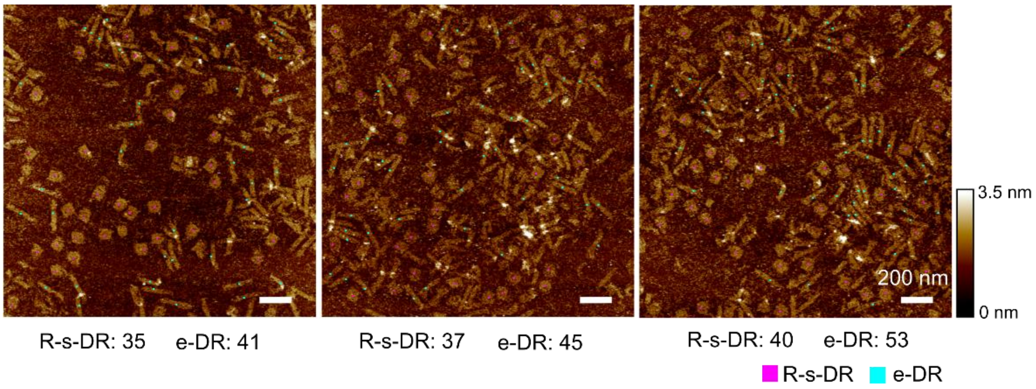

Fig. S2. AFM images of the R-s-DRs transformed from the e-DRs by the addition of locking strands (6-nt toehold extension). The transformation efficiency is about 46.3%. The analyzed R-s-DRs and e-DRs are indicated using magenta and cyan squares, respectively. Scale bars: 200 nm.

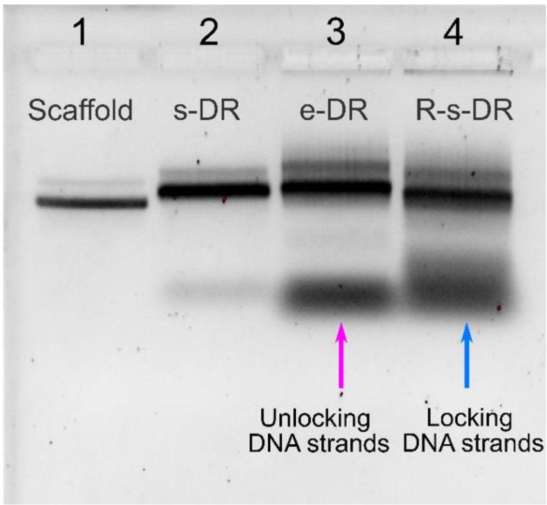

Fig. S3. Agarose gel (1%) analysis of different DNA structures. Lane 1: scaffold P7560. Lane 2: s-DRs. Lane 3: e-DRs transformed from the s-DRs by adding unlocking strands. Lane 4: R-s-DRs transformed from the e-DRs by adding locking strands.

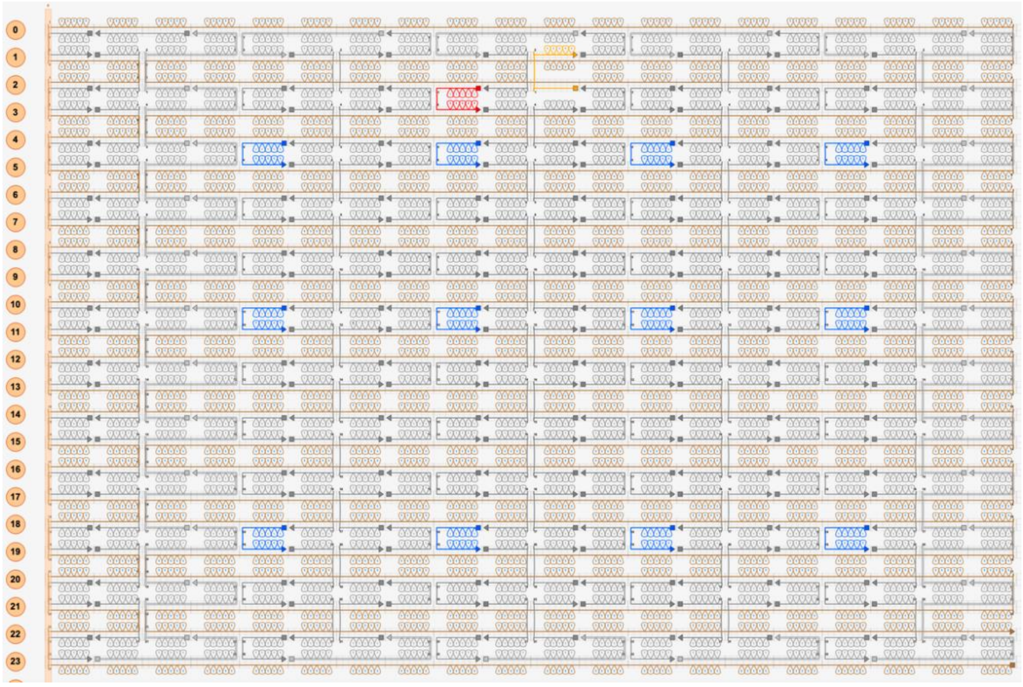

Fig. S4. caDNAno routing diagram of the s-DR, showing the positions of Cy3 (orange), Cy5 (red), and cholesterol modifications. The 12 dark blue staples are used for 12 cholesterol anchors.

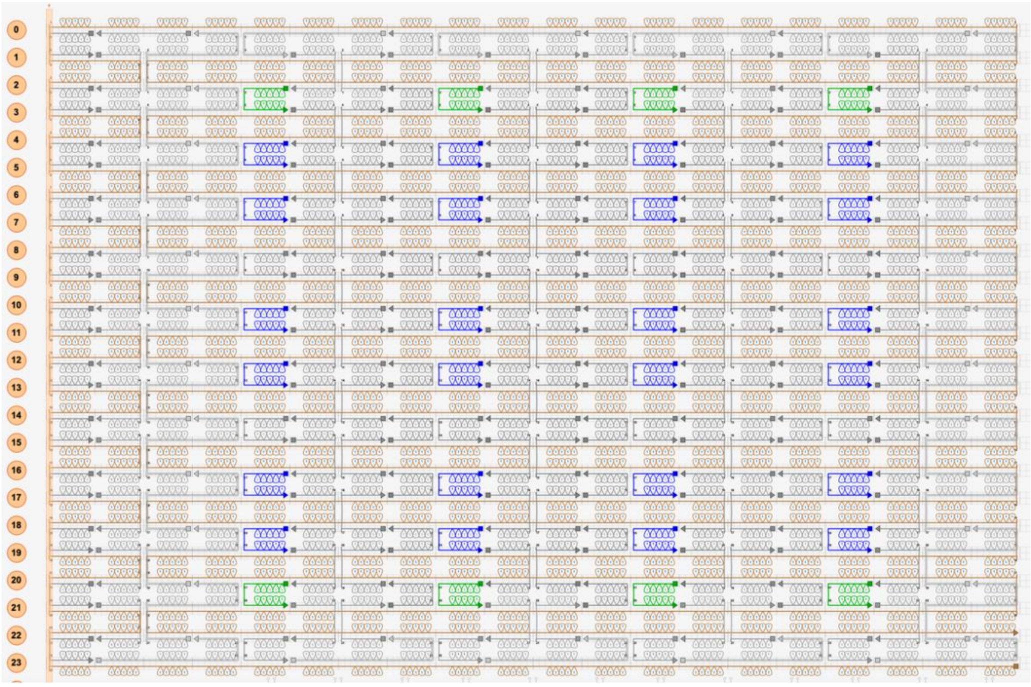

Fig. S5. caDNAno routing diagram of the s-DR, showing the positions of Atto488 and 24 cholesterol modifications. The 8 green-colored staple strands indicate the Atto488 modification positions. The 24 dark blue-colored staple strands indicate all the cholesterol anchor positions.

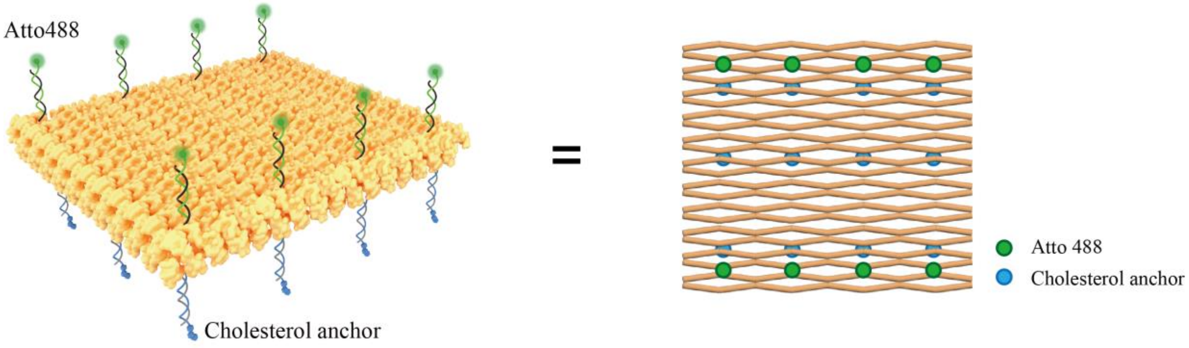

Fig. S6. Modification of the s-DR. The s-DR is modified with 12 cholesterol anchors (blue circles) for membrane binding and 8 Atto488 dyes (green circles) for confocal microscopy.

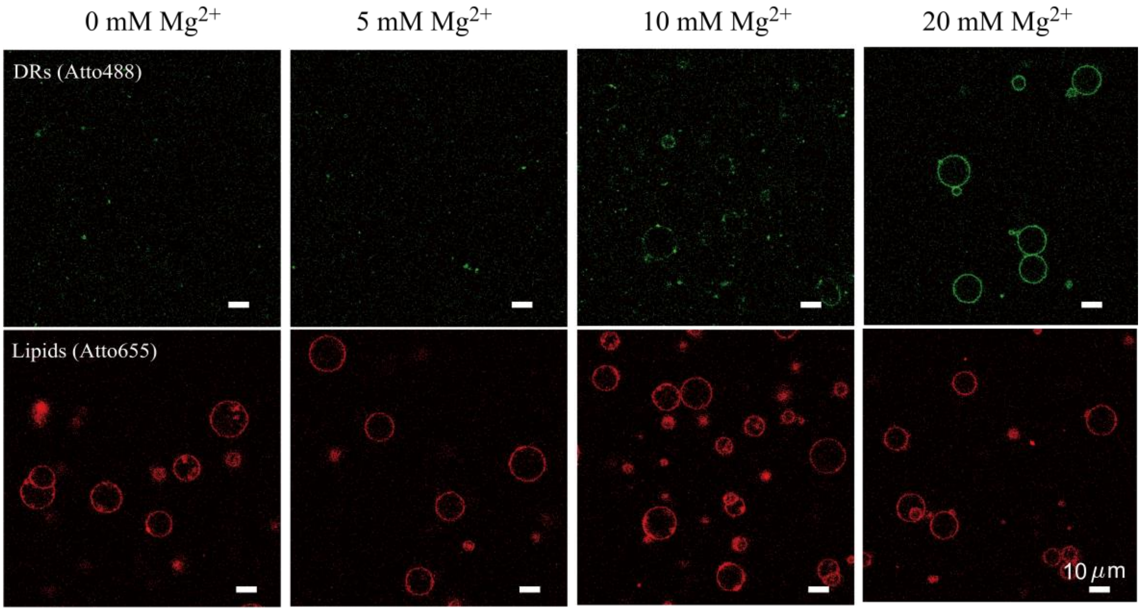

Fig. S7. Membrane binding of the s-DRs without cholesterol anchors under different Mg^{2+} concentrations. The results show that a high concentration of Mg^{2+} (20 mM) can enable the membrane binding of the s-DRs due to the non-specific electrostatic adsorption. At 5 mM, the non-specific electrostatic adsorption is largely inhibited. Therefore, the confocal imaging and AFM imaging in the subsequent experiments are performed at 5 mM Mg^{2+} to reduce nonspecific electrostatic adsorption of the DNA rafts on the membrane. Scale bars: 10 μm.

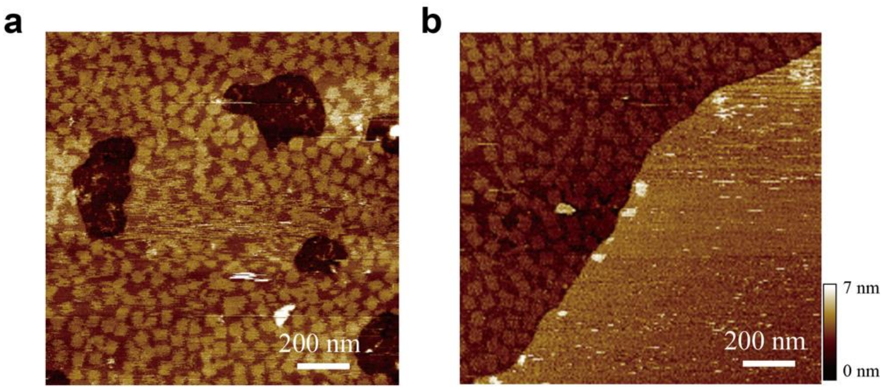

Fig. S8. (**a**) AFM image of the s-DRs (12-cholesterol anchors) on the SLB. The s-DRs are uniformly distributed on the SLB. (**b**) AFM image of the s-DRs (without cholesterol anchors). The s-DRs prefer to lie on mica.

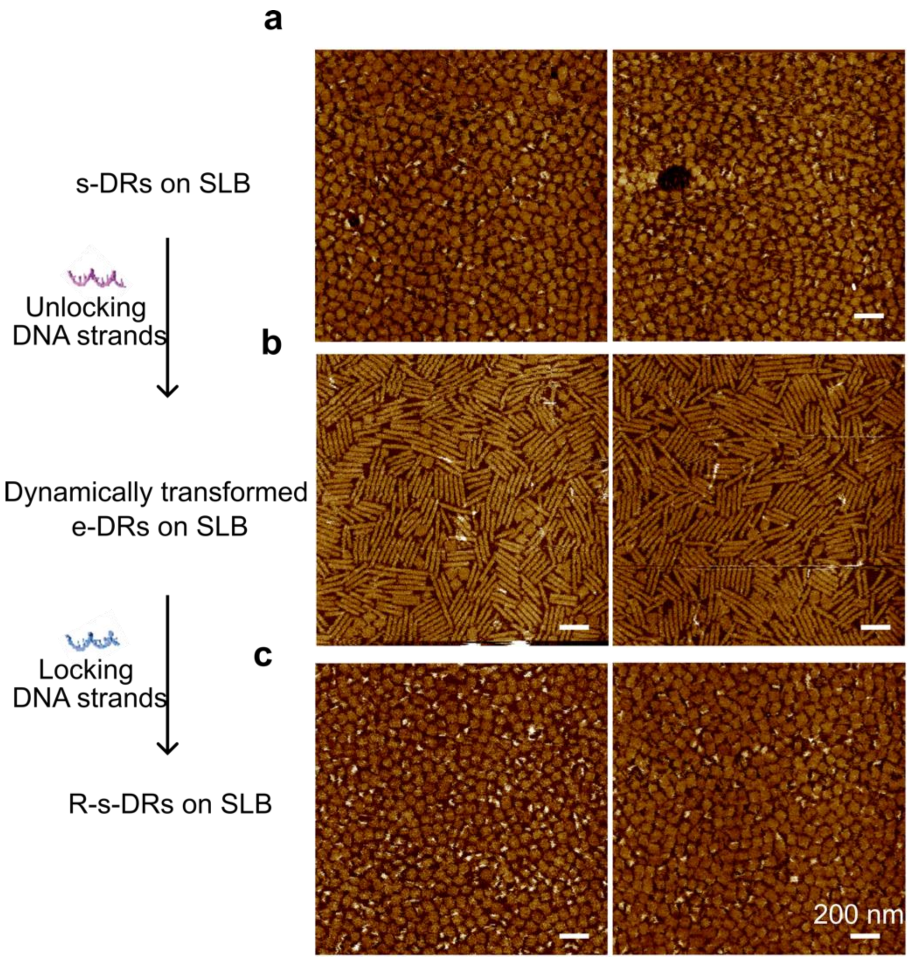

Fig. S9. Reversible conformation changes of the DRs on SLBs. (**a**) s-DRs on SLB. (**b**) Dynamically transformed e-DRs on SLB, showing local order [2,3]. (**c**) Dynamically transformed R-s-DRs on SLB, back to disorder. Scale bars: 200 nm.

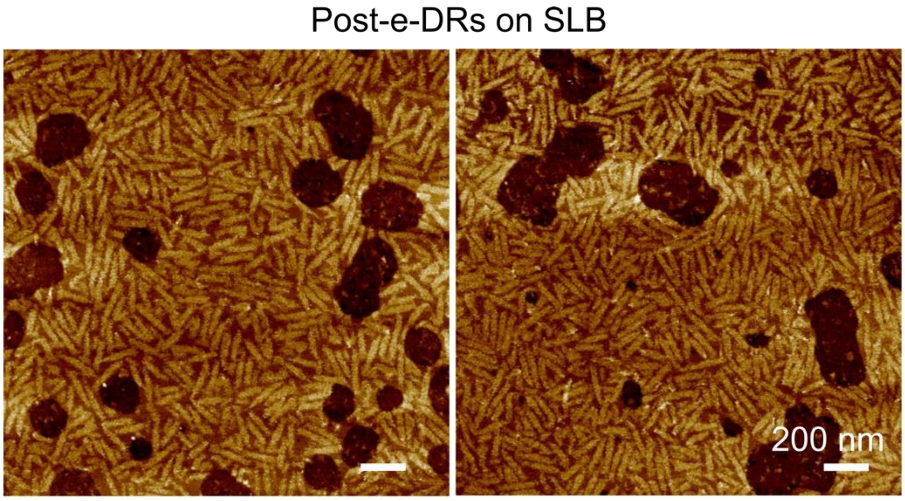

Fig. S10. Post-e-DRs on SLB. The post-e-DRs do not show prominent local order. Scale bars: 200 nm.

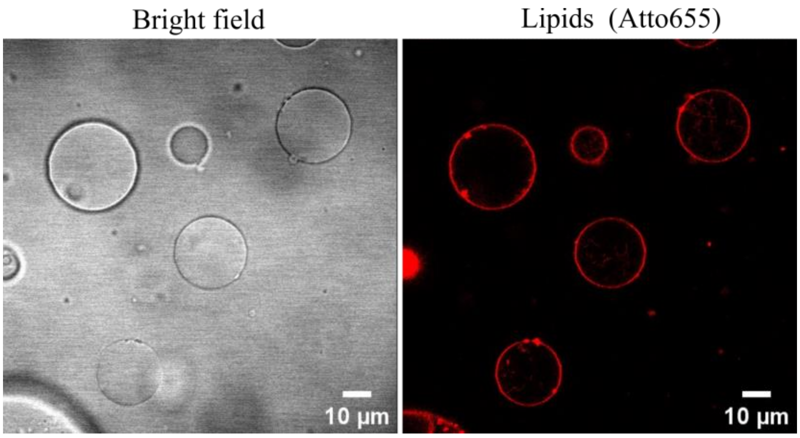

Fig. S11. Confocal fluorescence images of GUVs. Typically, the images are recorded at the equatorial planes of the GUVs.

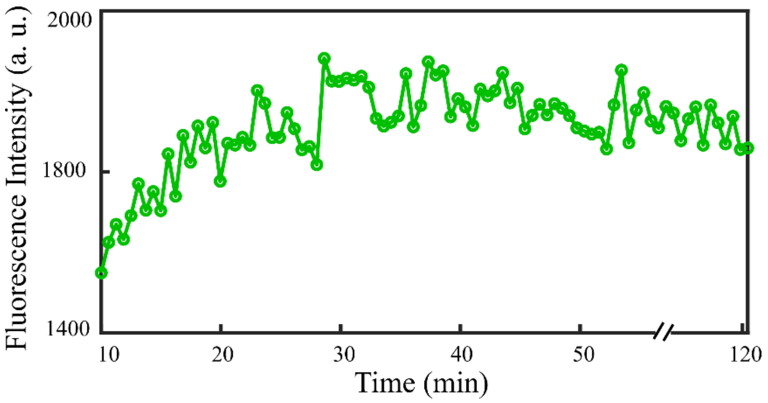

Fig. S12. Membrane binding of the s-DRs is quantitatively investigated by extracting the fluorescence intensities. The binding reaches an equilibrium after ~ 30 minutes of incubation.

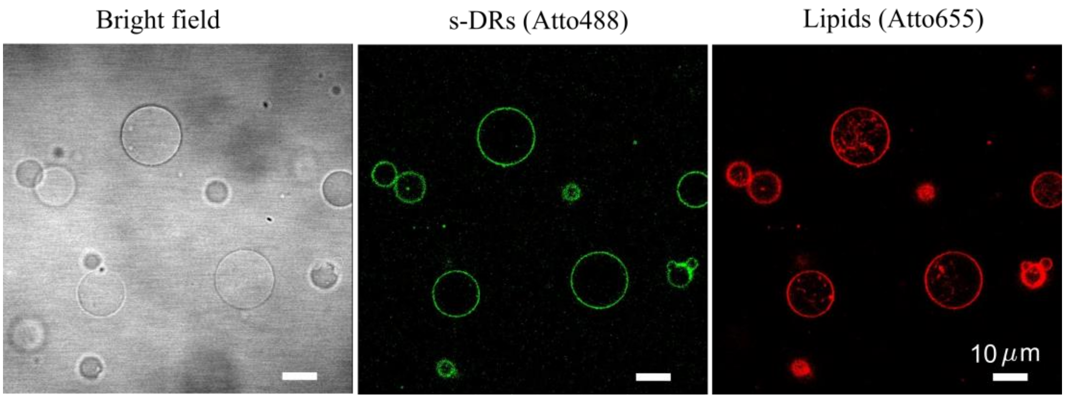

Fig. S13. Confocal images of GUVs with Atto488 labeled s-DRs (green) and Atto655 labeled lipids (red). The images are recorded at the equatorial planes of the GUVs. After reaching an equilibrium, the fluorescence signals of the s-DRs and the lipids colocalize, confirming the successful binding of the DNA rafts on the GUV membrane. Scale bars: 10 μm.

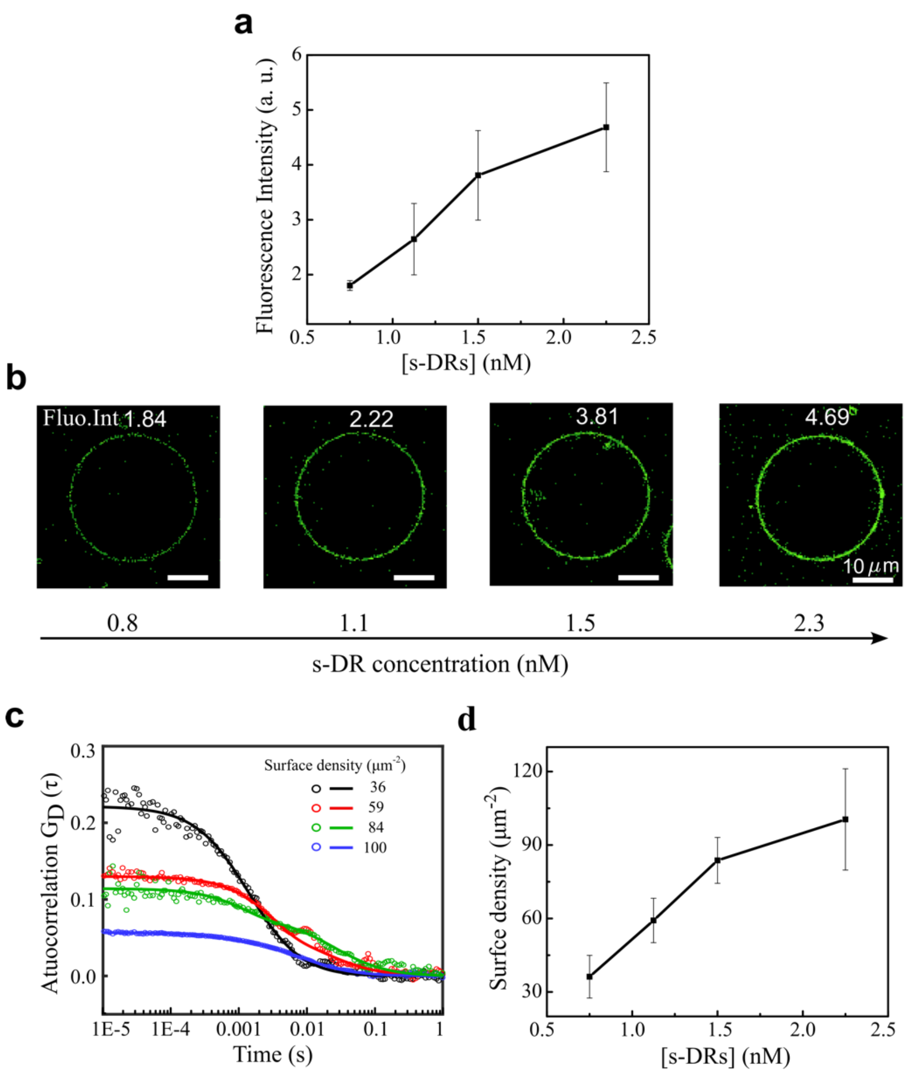

Fig. S14. Calculation of the surface density by the DNA rafts on GUVs. (a) Fluorescence intensities of the membrane-bound s-DRs (labeled with 8 Atto488 dyes) at equilibrium (incubated for 1h) are extracted using image analysis by MATLAB (see methods) and represented as a function of total bulk. Data represent mean ± SEM from three independent experiments. n for 0.75 nM, 1.125 nM, 1.5 nM and 2.25 nM are 82, 98, 100 and 59, respectively. (**b**) Representative confocal images at the equatorial plane for membrane-bound s-DRs. (**c**) Representative FCS autocorrelation curves for membrane-bound s-DRs on the upper pole of a GUV at four different surface densities. Circle symbols represent the raw data and the solid curves represent their fits using the data analysis software PyCorrFit [4] version 0.8.2. (**d**) s-DR surface density on the GUV determined by FCS as a function of the s-DR concentration. The 488 nm line of the argon ion laser is used for the Atto488 excitation with LSM 980. Data represent mean ± SEM from three independent experiments (n = 6 for each concentration). Detailed parameters for the determination of surface density and surface coverage of the s-DRs on GUVs can be found in Table S11-S14. Scale bars: 10 μm.

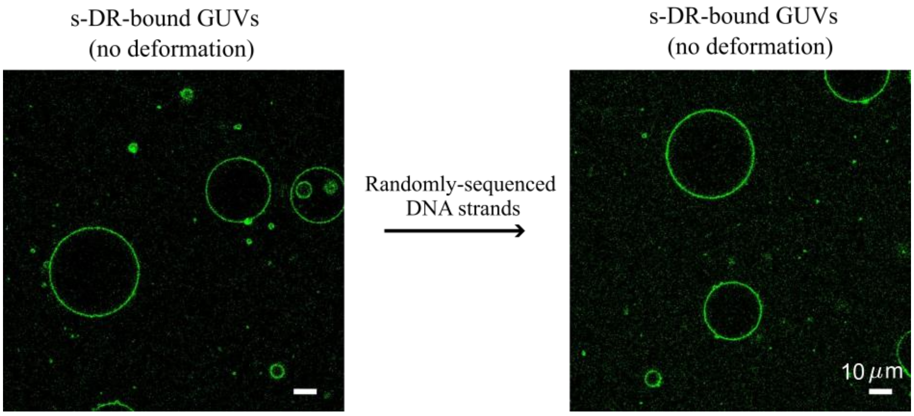

Fig. S15. Addition of randomly-sequenced DNA strands does not trigger the conformation change from the s-DRs to e-DRs and no membrane remodeling is observed. Scale bars: 10 μm.

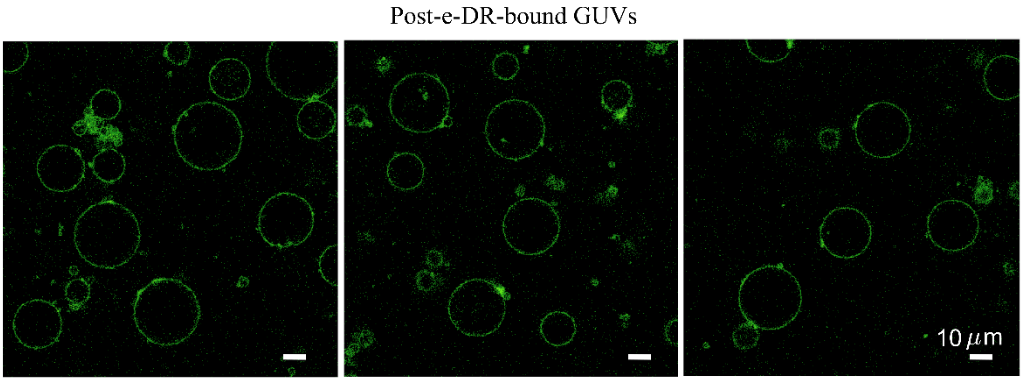

Fig. S16. GUVs remain spherical after incubation with the post-e-DRs. Scale bars: 10 μm.

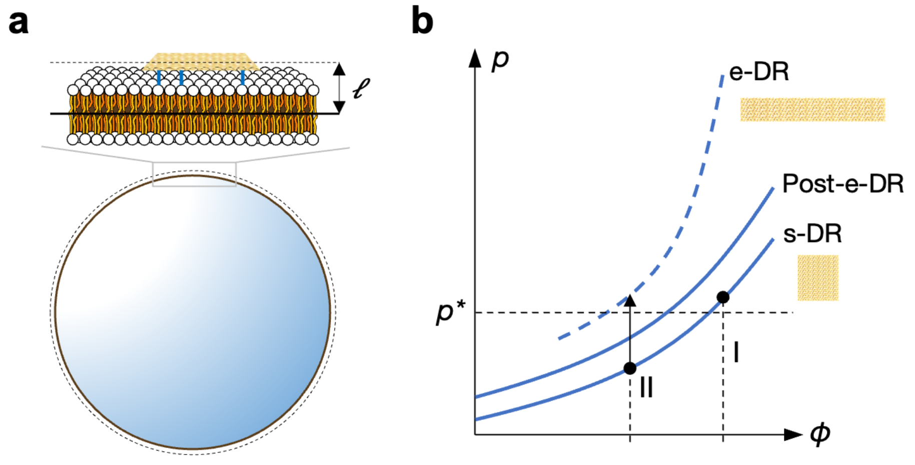

Fig. S17. Qualitative picture how crowding with DNA rafts can drive membrane deformations. (**a**) Sketch of the GUV. The DNA rafts move on the curved surface (dashed line) displaced from the center line (thick solid line) of the bilayer membrane by a length ℓ due to the cholesterol anchors (blue). (**b**) Interactions among rafts (mainly due to their excluded volume) induces a pressure p_α (solid lines) that is increased with coverage φ (fraction of area occupied by the rafts). Beyond a threshold p^* the system can reduce the total free energy by deforming the GUV (which increases the bending energy but also increases the area available to the rafts). For large coverage (I) the s-DRs cross the threshold and induce deformation (Table S3 and S4). For small coverage (II), the spherical shape is stable but triggering the conformational change to e-DRs, which induces strong local order with an increased pressure (dashed line) that crosses the threshold at lower coverages. The post-e-DRs lack the local order and remain isotropic, implying a pressure that increases more gently. The basic mechanism for the destabilization of the spherical GUV shape by the conformation change of the DNA rafts from the s-DRs to e-DRs can be corroborated through a theoretical analysis based on the coupled free energy of the vesicle and rafts. Our analysis follows Stachowiak *et al.*, who suggested that protein crowding on a membrane was able to drive membrane deformations [5]. The rafts move in a two-dimensional manifold that is slightly displaced by a length ℓ from the vesicle due to the cholesterols. The rafts thus access an area $\tilde{A} > A$ that is larger than the membrane area A. The excluded volume of the rafts generates a pressure $p_\alpha(\varphi)$ that depends on conformation $\alpha = s, e$ (square or elongated) and coverage φ. For large pressures, the membrane can make more area available to the rafts, and thus lower the total free energy $F = F_0 - p(\tilde{A} - A)$, through transitioning to a non-spherical shape with F_0 the free energy cost to deform the membrane [6]. The detailed analysis yields a critical pressure p^* beyond which the spherical shape loses its stability. The critical pressure is either crossed for squares at large coverage (Table S3 and S4) or through changing the conformation, which induces local order (Fig. S9c) and effectively “jams” the nanorafts as evident from the vanishing diffusion coefficient (Fig. S27). This implies a large pressure. The post-e-DR assemble without the strong short-range order (Fig. S10) and remain mobile, which implies a smaller pressure below the critical pressure p^*. The perforation of the membrane and the formation of synthetic channels influence the osmotic pressure difference. The GUV adapts volume and area in the presence of the e-DRs, which we posit increases the critical pressure, thus restoring the spherical shape.

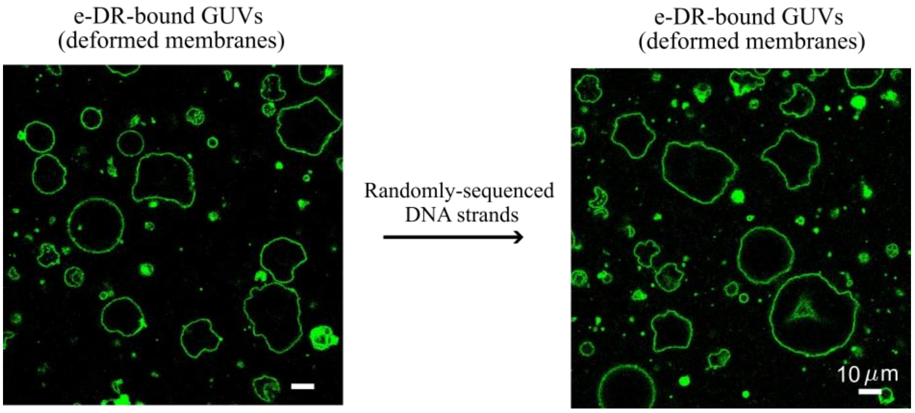

Fig. S18. Addition of randomly-sequenced DNA strands does not trigger the conformation change from the e-DRs to R-s-DRs and the GUVs remain deformed. Scale bars: 10 μm.

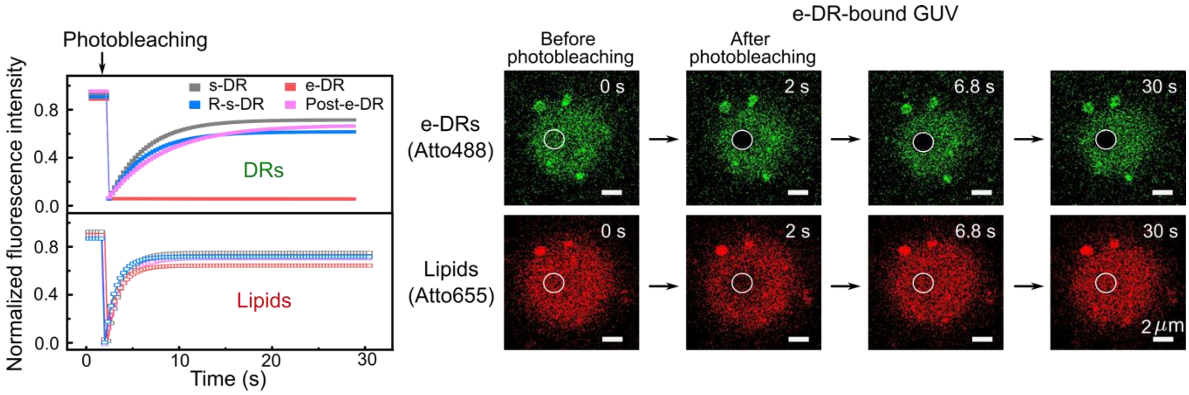

Fig. S19. Diffusion properties of the s-DRs (grey), e-DRs (red), R-s-DRs (blue) and post-e-DRs (pink) on the upper pole of GUVs as well as the corresponding GUV lipids characterized by fluorescence recovery after photobleaching (FRAP). After FRAP, the fluorescence signal of the Atto488-labeled e-DRs is not recovered (top right), whereas that of the Atto655-labeled lipids is recovered (bottom right). These results demonstrate that the s-DRs, R-s-DRs, and post-e-DRs are highly mobile on the membrane. On the contrary, the interacting e-DRs self-arrange in local order display low mobility and can effectively sculpt the GUV morphology. Scale bars: 2 μm.

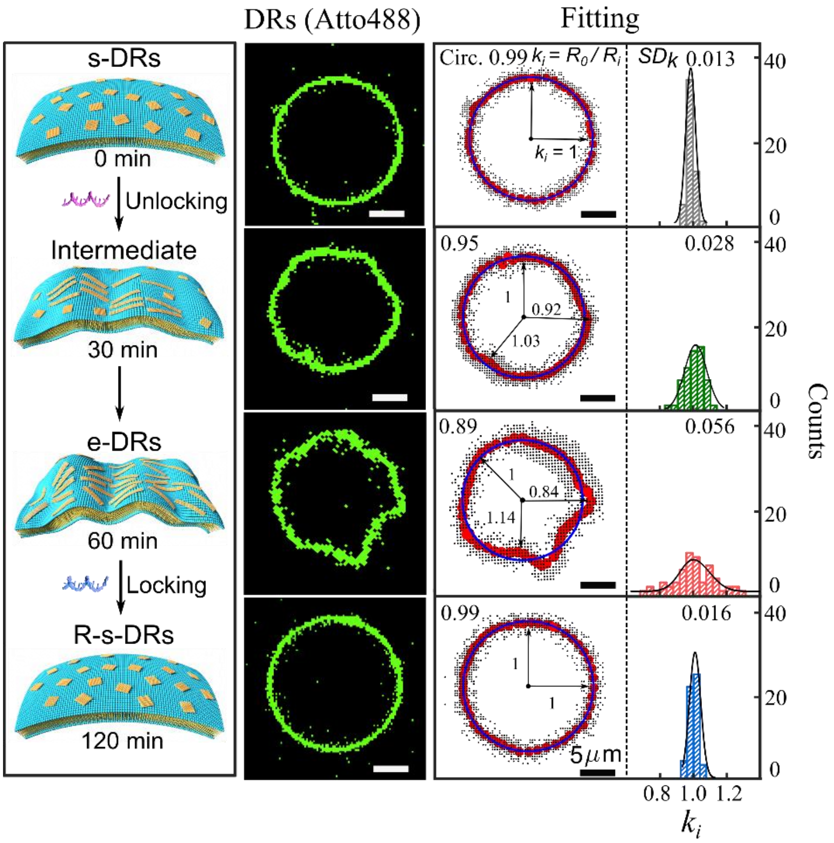

Fig. S20. Evaluation of the GUV reshaping at different states by shape tracing of the DNA raft-bound GUV slices and calculation of the normalized local curvature k_i and the standard deviation of the normalized local curvature SD_k of the GUVs. k_i and SD_k are used to evaluate the degree of GUV remodeling by shape tracing and fitting the contours of the GUVs using MATLAB (See methods and Supplementary Video 4) according to reference [7,8] with minor modifications from raw GUV images (512 × 512 pixels). The results (Fitting column) show that the distribution of k_i is much broader in the cases of the intermediate-state DNA rafts and e-DRs, but much shaper in the cases of the s-DRs and R-s-DRs. SD_k experiences a transition from 0.013 to 0.028, to 0.056, and to 0.016.

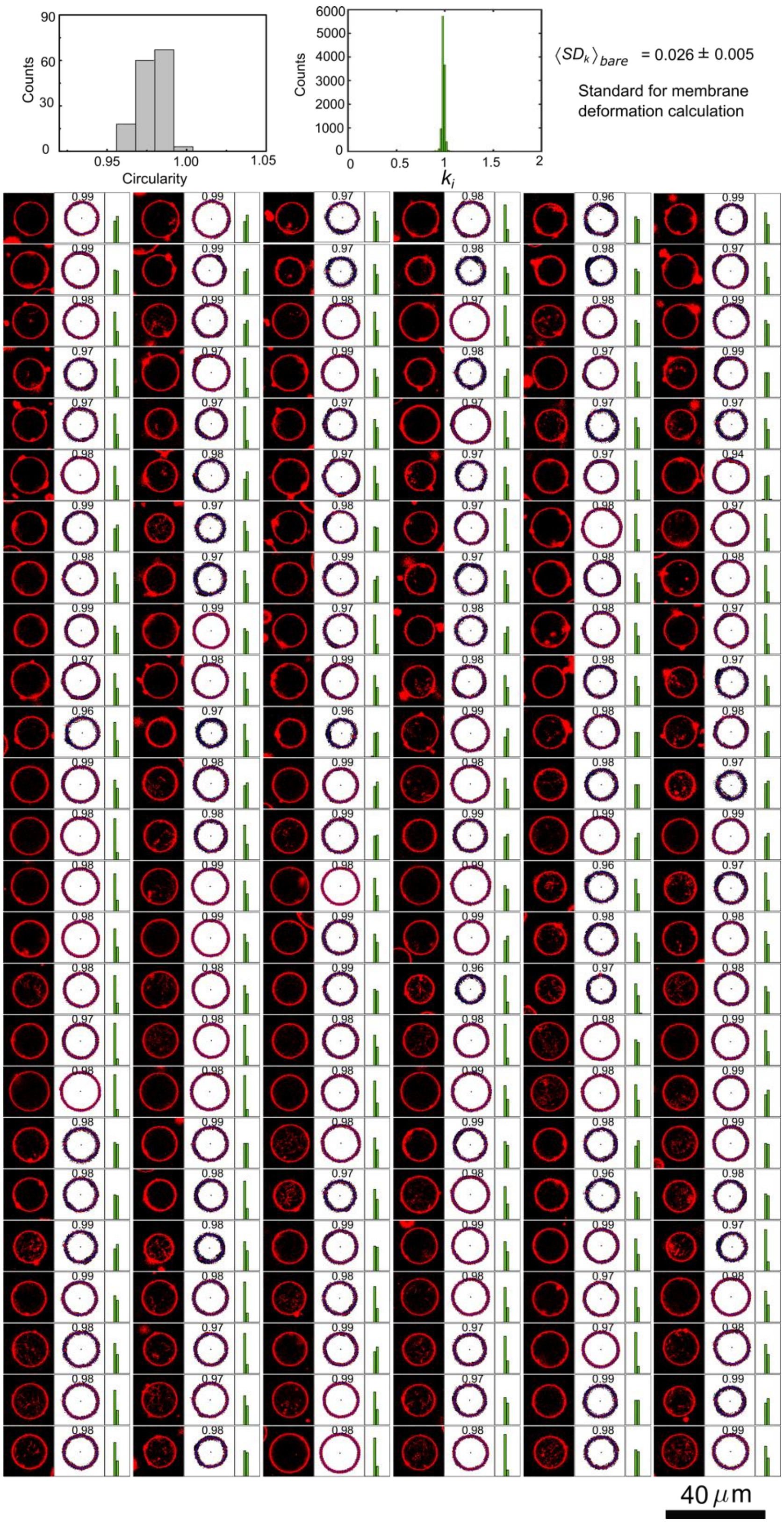

Fig. S21. Confocal images and calculation of $\langle SD_k \rangle$ of 150 bare GUVs membranes (without binding of the DNA rafts). The mean value, $\langle SD_k \rangle$ of 150 bare GUV membranes is used as the standard value for calculating the membrane deformation efficiencies. GUVs are considered deformed, if values of their SD_k are higher than $\langle SD_k \rangle_{bare} = 0.026$. Scale bare: 40 μm.

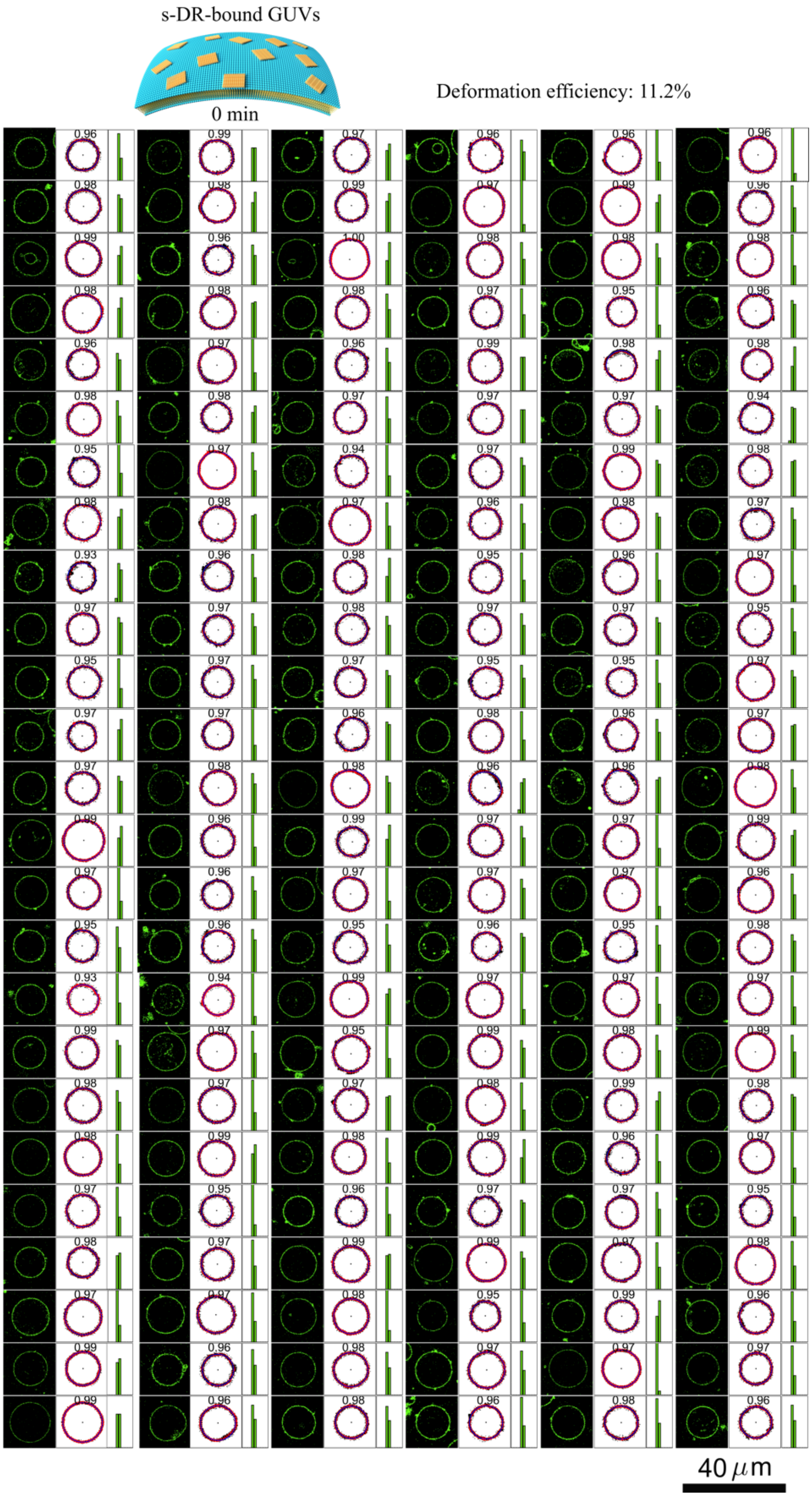

Fig. S22. s-DR-bound GUVs. The deformation of GUVs by the s-DRs is weak with deformation efficiency ~11.2%. Scale bare: 40 μm.

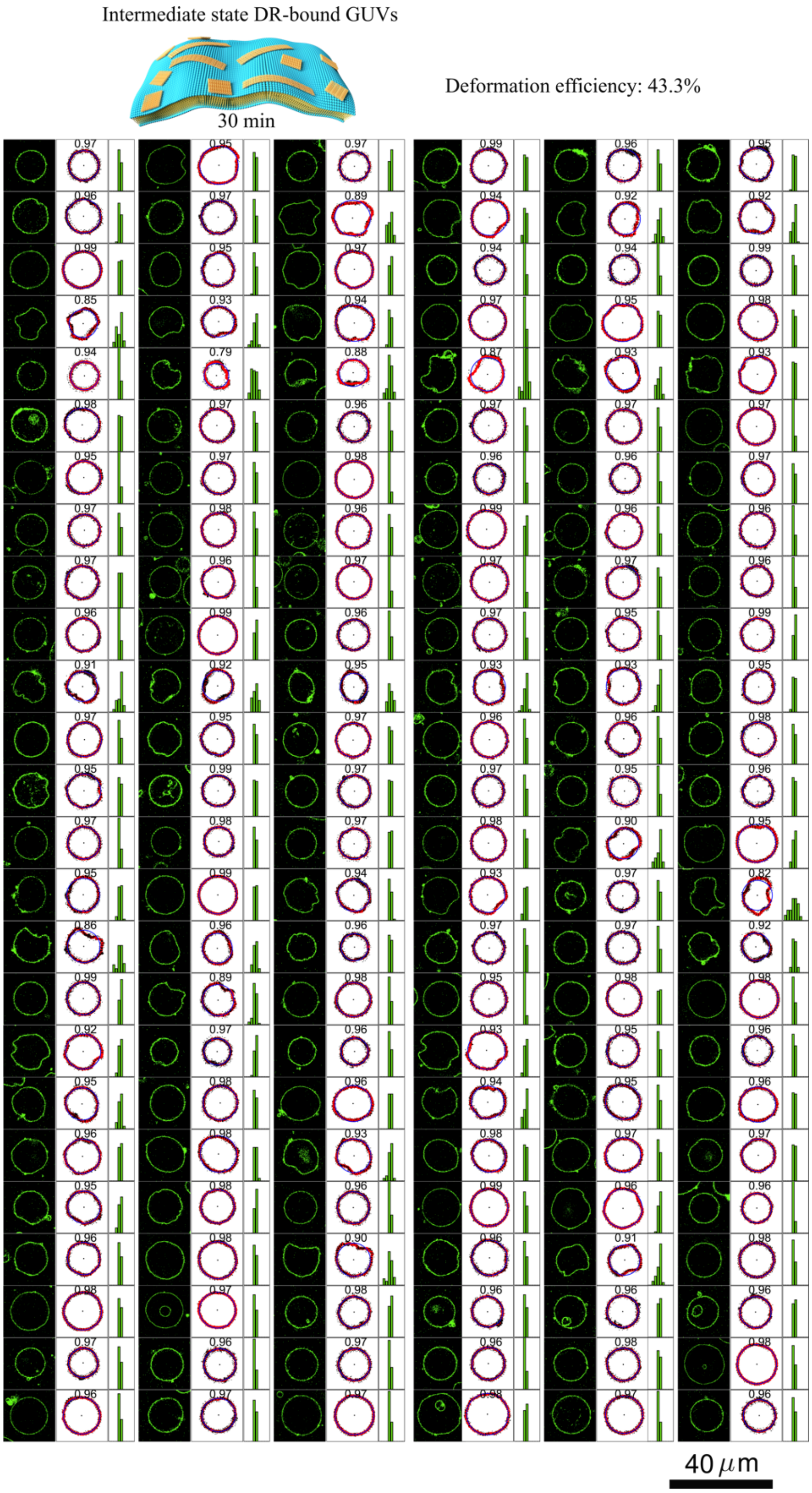

Fig. S23. Intermediate state DR-bound GUVs. After addition of unlocking strands for 30 min, the deformation efficiency is increased to ~ 43.3%. Scale bare: 40 μm.

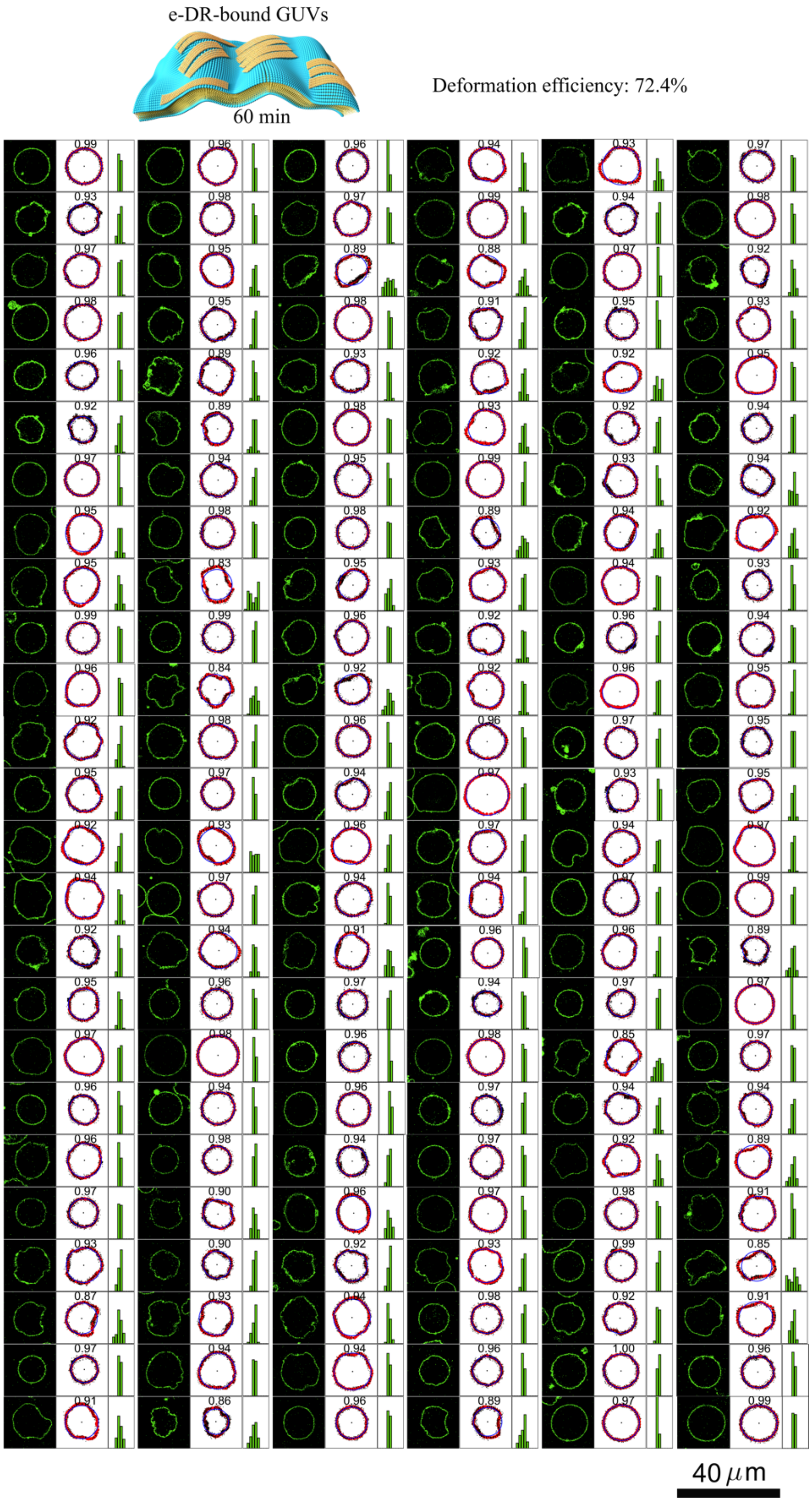

Fig. S24. e-DR-bound GUVs. After addition of unlocking strands for 60 min, the s-DRs are transformed to e-DRs and the deformation efficiency is ~ 72.4%. Scale bare: 40 μm.

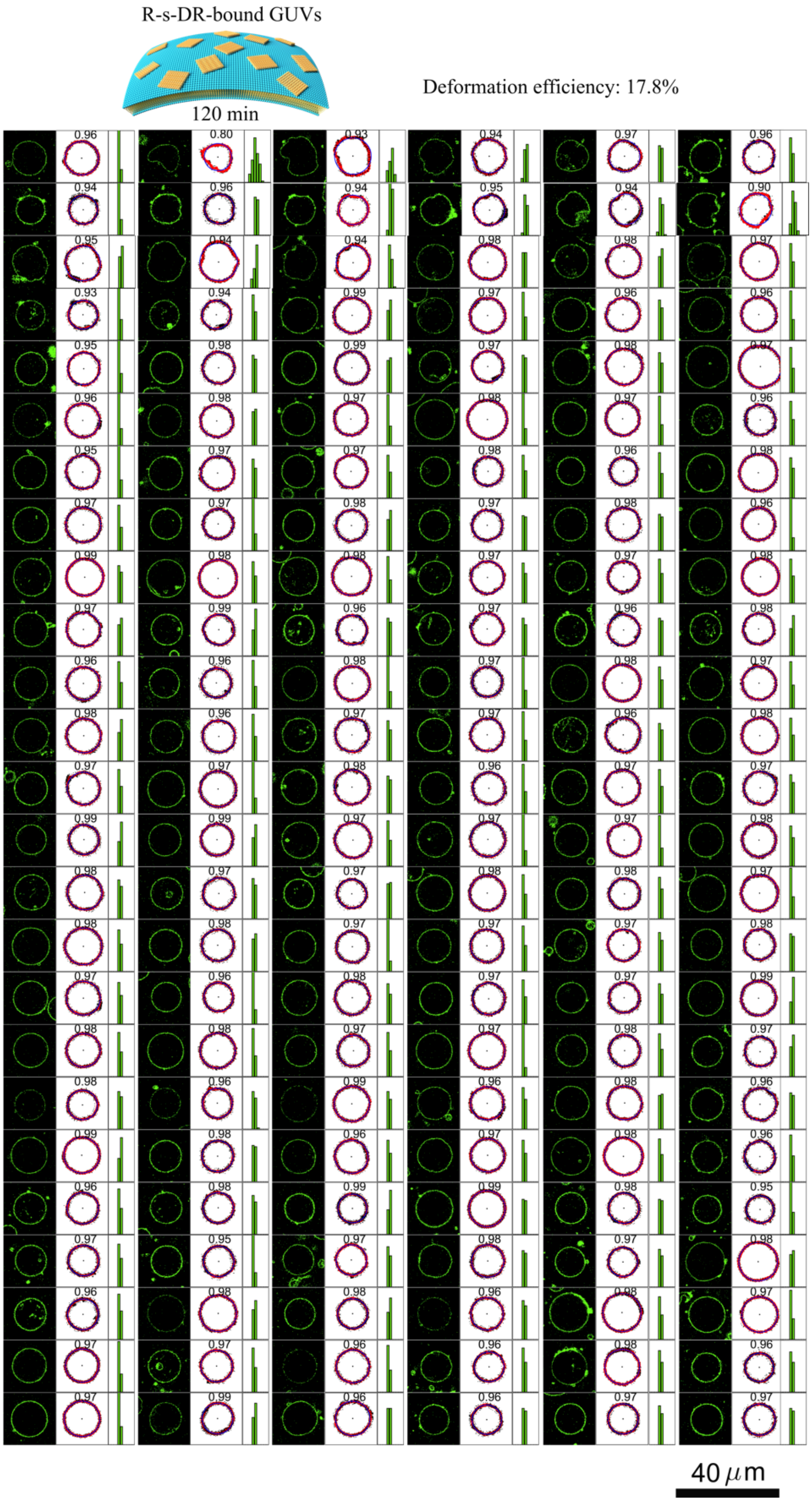

Fig. S25. R-s-DR-bound GUVs. Reconfiguration of the e-DRs to R-s-DRs. The deformation efficiency is decreased to ~17.8%. Scale bare: 40 μm.

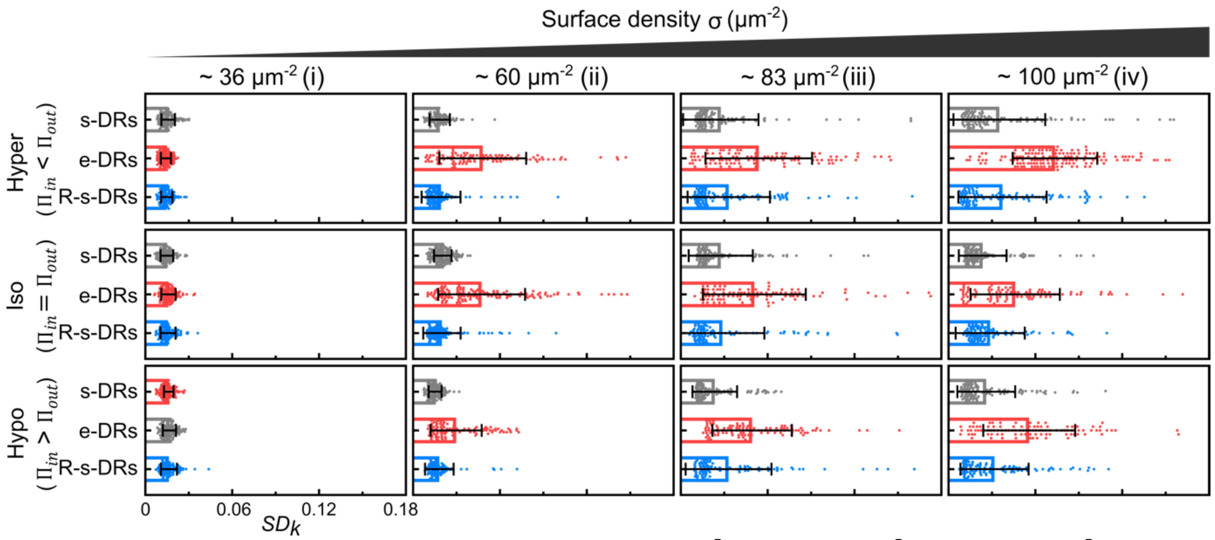

Fig. S26. Influence of the surface density σ = ~ 36 μm^{-2} (i), ~ 60 μm^{-2} (ii), ~ 83 μm^{-2} (iii), and ~ 100 μm^{-2} (iv) of the DNA rafts and osmotic pressure applied to GUVs (isosmotic buffer, hyper- or hypoosmotic buffers) on the degree of GUV remodeling. Data represent mean ± SD from three independent experiments. Representative confocal images of GUVs are shown in Supplementary Data. At a low σ = ~ 36 μm^{-2} (Fig. S26 (i)), neither the conformation state of the DNA rafts, nor osmolarity notably affects the GUV morphology (Table S1). n for s-DRs, e-DRs, and R-s-DRs are as follows: 113, 107, and 106 in hyper buffer; 106, 110, and 107 in iso buffer and 106, 106, and 109 in hypo buffer, respectively. By increasing σ to ~ 60 μm^{-2} (Fig. S26 (ii)), substantial differences are resolved among the three states. Specifically, under the isosmotic condition ($\Pi_{in} = \Pi_{out}$), the distribution of SD_k is only slightly broadened at the s-DR state, whereas SD_k at the e-DR state becomes exceedingly dispersive, revealing a high degree of GUV remodeling. At the R-s-DR state, approximately 17.8% of the GUVs are not fully recovered (Table S2). This could result from the nonperfect transformations of the DNA rafts, which tightly pin the GUVs at the e-DR state and refrain their morphology from recovery. Furthermore, it is evident that hyperosmotic ($\Pi_{in} < \Pi_{out}$) and hypoosmotic ($\Pi_{in} > \Pi_{out}$) stress enhances and restrains the GUV remodeling due to the deflated (low tension) and inflated (high tension) nature of the GUVs, respectively. n for s-DRs, e-DRs, and R-s-DRs are as follows: 110, 110, and 108 in hyper buffer; 152, 152, and 152 in iso buffer and 119, 107, and 106 in hypo buffer, respectively. With a further increase to ~ 83 μm^{-2} (Fig. S26 (iii)), σ starts to play a dominating role. Even under hypoosmotic stress, the s-DRs can readily induce GUV deformations (Table S3). n for s-DRs, e-DRs, and R-s-DRs are as follows: 114, 106, and 110 in hyper buffer; 117, 106, and 106 in iso buffer and 115, 108, and 106 in hypo buffer, respectively.When σ is increased to ~ 100 μm^{-2} (Fig. S26 (iv)), the differences among the three states become ambiguous under different osmolarity conditions (Table S4).). n for s-DRs, e-DRs, and R-s-DRs are as follows: 119, 149, and 110 in hyper buffer; 130, 108, and 119 in iso buffer and 120, 117, and 116 in hypo buffer, respectively. The decreased diffusion coefficients at high σ also supported these results (Fig. S27 In the following, if not particularly mentioned, σ = ~ 60 μm^{-2} and an isosmotic condition are applied, as they give rise to the most distinct behavior among different states.

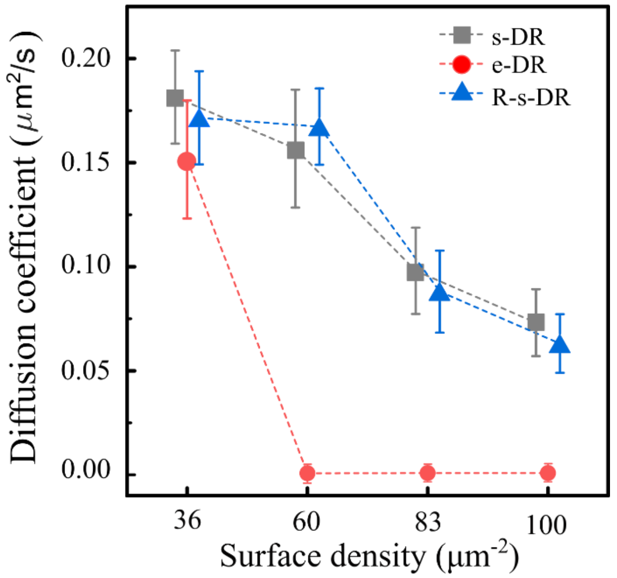

Fig. S27. Lateral membrane diffusion coefficients of the DRs at different states as a function of the surface density of the s-DRs calculated from three independent FRAP experiments. Data represent mean ± SD (n = 9).

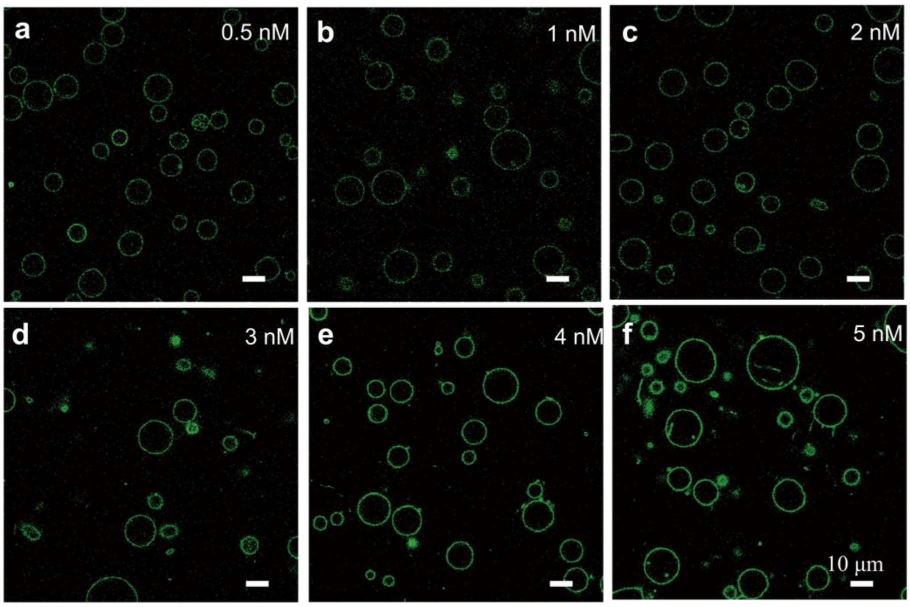

Fig. S28. Binding of free, non-patterned cholesterol anchors. Cholesterol-modified single-stranded DNA strands (fluorescently labeled with carboxyfluorescein (FAM)) of different concentrations are incubated with GUVs. The results show that GUV remodeling is negligible. Scale bars: 10 μm.

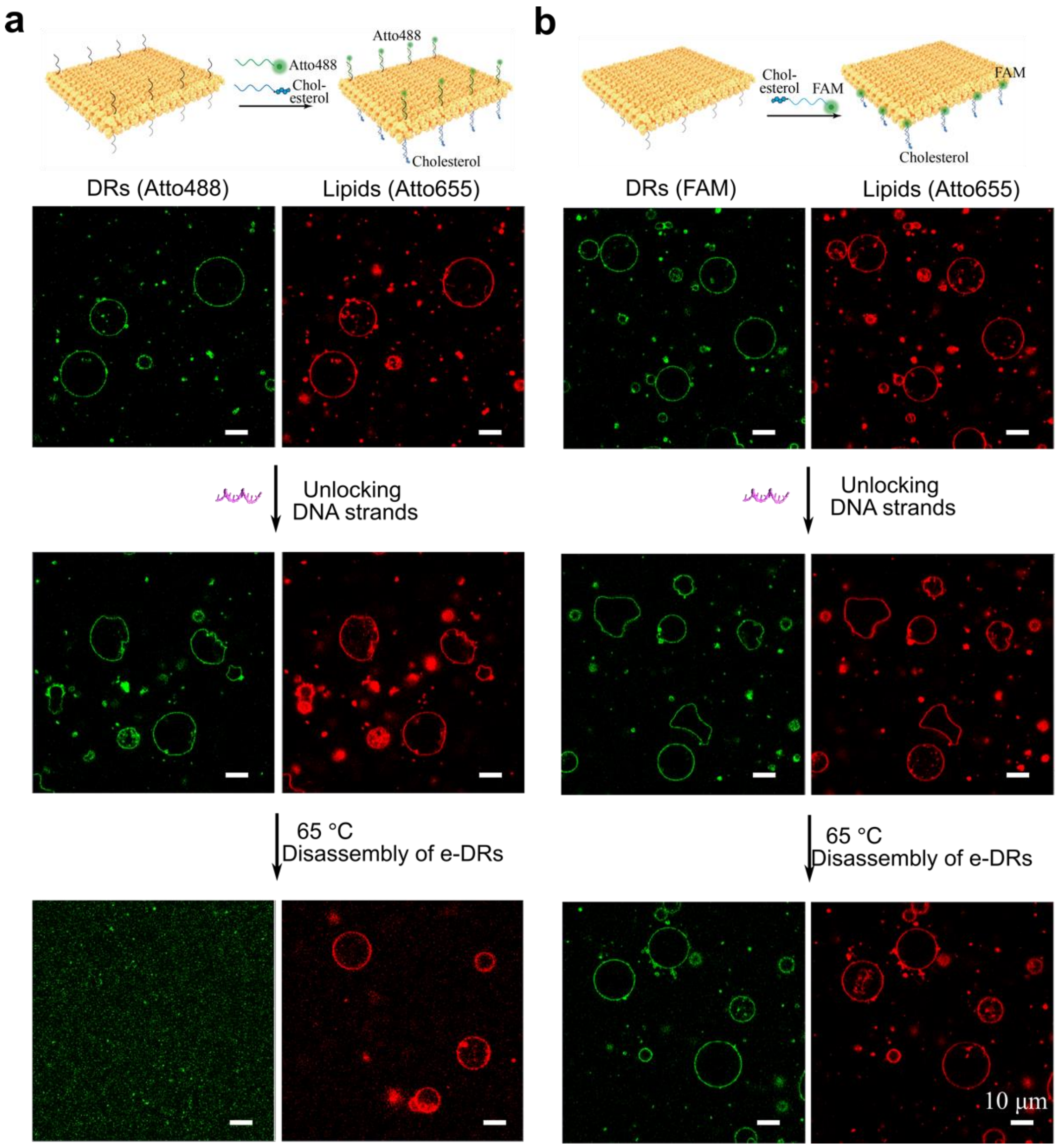

Fig. S29. (**a**) Atto488-labeled s-DRs are transformed to e-DRs on Atto655-labled GUVs by the addition of unlocking strands, yielding GUV deformations. Increasing the temperature to 65 °C leads to the dissociation of the DNA rafts from the GUVs, which results in their spherical shape recovery. (**b**) Cholesterol anchors on the s-DRs are labeled with FAM but the s-DRs are not labeled. Transformation of the s-DRs to e-DRs on the GUVs leads to deformations. Increasing the temperature to 65 °C gives rise to the dissociation of the DNA rafts from the GUVs. The cholesterols remain and become non-patterned on the GUVs. No GUV deformations are visible. Scale bars: 10 μm.

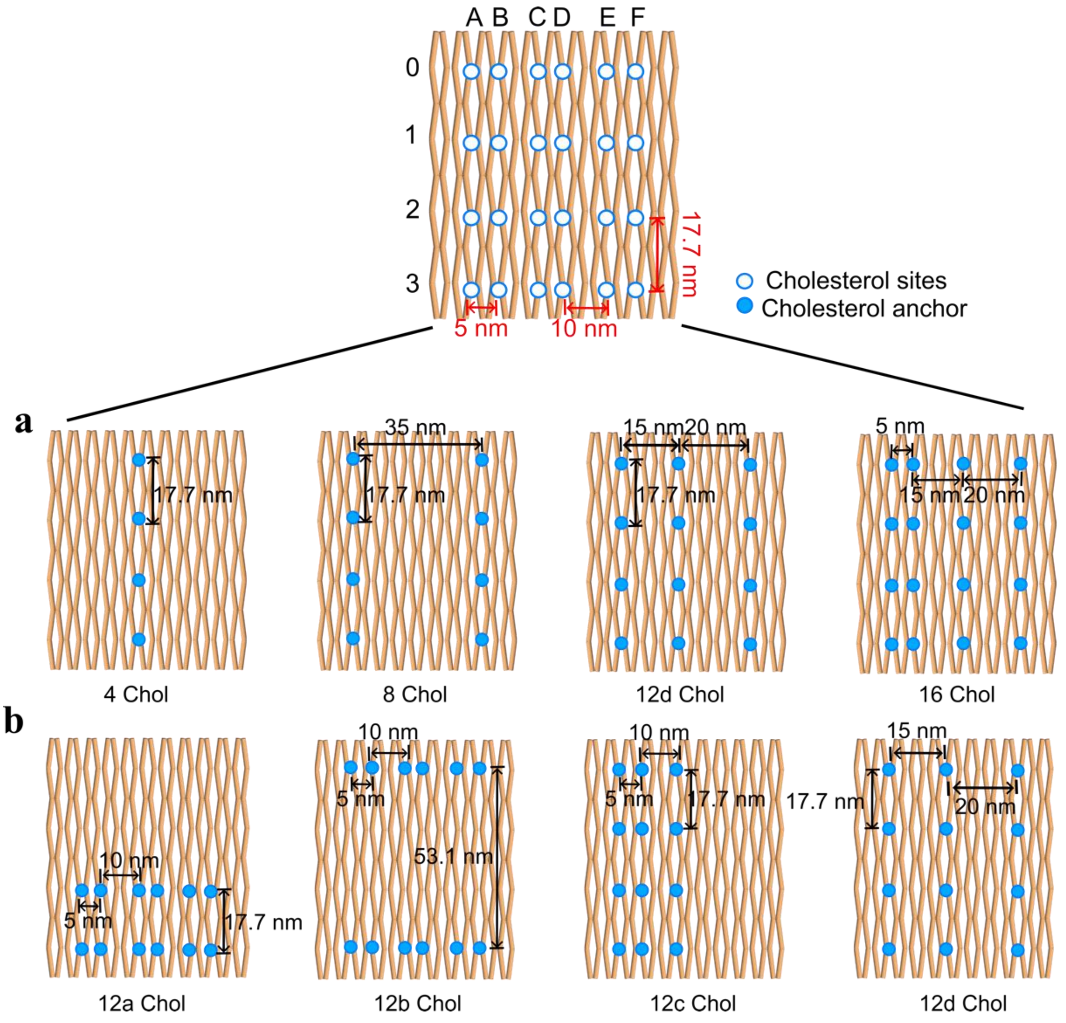

Fig. S30. (**a**) Different cholesterol numbers (4, 8, 12 and 16). (**b**) 12 cholesterol sites in 4 different patterns.

a

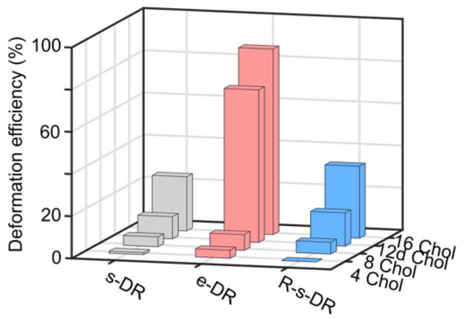

	4 Chol	8 Chol	12d Chol	16 Chol
**s-DR-bound GUVs**	1% (n=105)	5.6% (n=107)	11.2% (n=152)	25.7% (n=109)
**e-DR-bound GUVs**	2.9% (n=105)	8.4% (n=107)	72.4% (n=152)	87.9% (n=116)
**R-s-DR-bound GUVs**	0 (n=105)	5.6% (n=107)	17.8% (n=152)	34.1% (n=126)

b

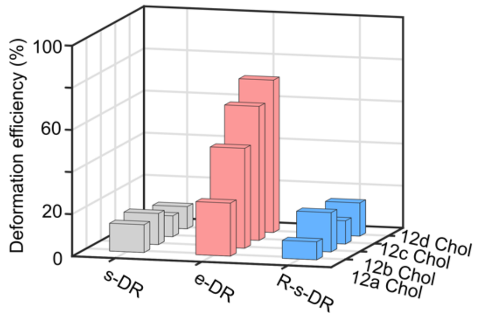

	12a Chol	12b Chol	12c Chol	12d Chol
**s-DR-bound GUVs**	13.2% (n=106)	15.7% (n=115)	9.9% (n=111)	11.2% (n=152)
**e-DR-bound GUVs**	26.1% (n=115)	48.3% (n=118)	64.5% (n=110)	72.4% (n=152)
**R-s-DR-bound GUVs**	9.4% (n=117)	18.7% (n=107)	11% (n=109)	17.8% (n=152)

Fig. S31. Comparison of the membrane deformation efficiencies for different cholesterol patterns. (**a**) Different cholesterol numbers. (**b**) 12 cholesterol sites distributed in different patterns. It is worth mentioning that 12d corresponds to the pattern used in Fig. 1 and Fig. 2a. 12d shows the narrowest SD_k distribution, whereas 12a, 12b, and 12c all lead to GUV deformations to different extents. This can be understood as follows: in the case of 12d, the cholesterol sites are evenly far spaced among one another, whereas for 12a, 12b, and 12c, closely-spaced cholesterol sites facilitate the firm binding of the DNA rafts on the membranes. However, after the reconfiguration to the e-DR state, 12d generates the highest degree of GUV deformations. This can be attributed to the fact that the cholesterol sites are tightly spaced and arranged in parallel lines, introducing strong anchoring of the DNA rafts to the membrane. Meanwhile, these lines are widely expanded from the middle to the two ends, which help to well establish the elongated shape of the DNA raft. As a result, 12d that is associated with the most distinct cholesterol pattern alteration from the s-DR to e-DR state gives rise to the highest degree of GUV remodeling.

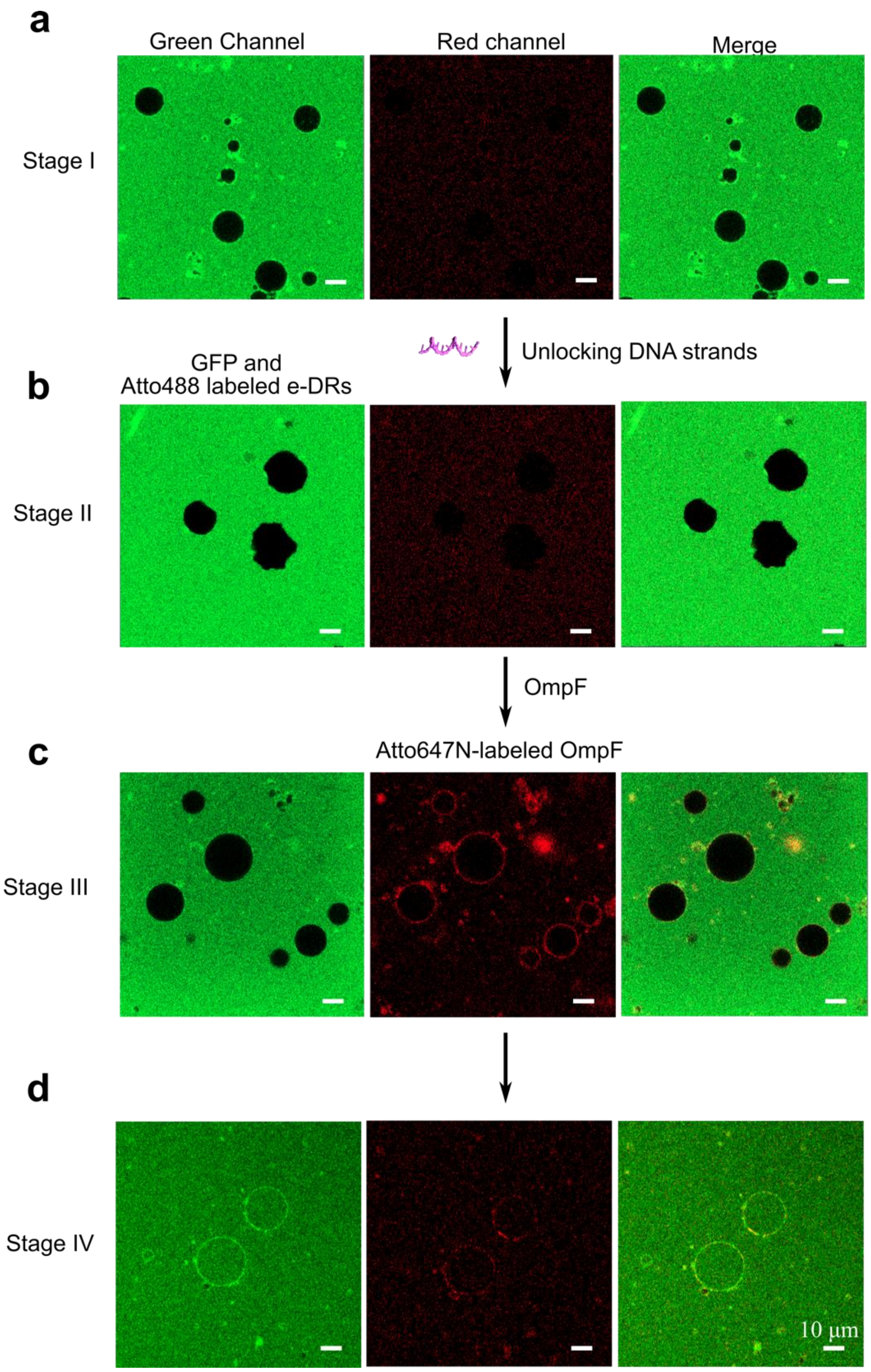

Fig. S32. Membrane perforation and GFP influx through the synthetic channels. For the results in the manuscript in Fig. 3a, the DNA rafts are labeled with Cy3, so that the GFP-filled GUV can be clearly visualized by distinguishing the border of GUV. In this independent experiment, the DNA rafts are marked with Atto488 to clearly distinguish from Atto647N-modified OmpF. OmpF can mediate the exchange of small solutes (sucrose, Na^+, Mg^{2+}, Cl^-, etc.) to restore osmotic balance across the membrane. More specifically, sucrose (220 mM) from the interior and $MgCl_2$ (5 mM), NaCl (92.5 mM) from the exterior can diffuse through OmpF out of and into the GUV, respectively. Scale bars: 10 μm.

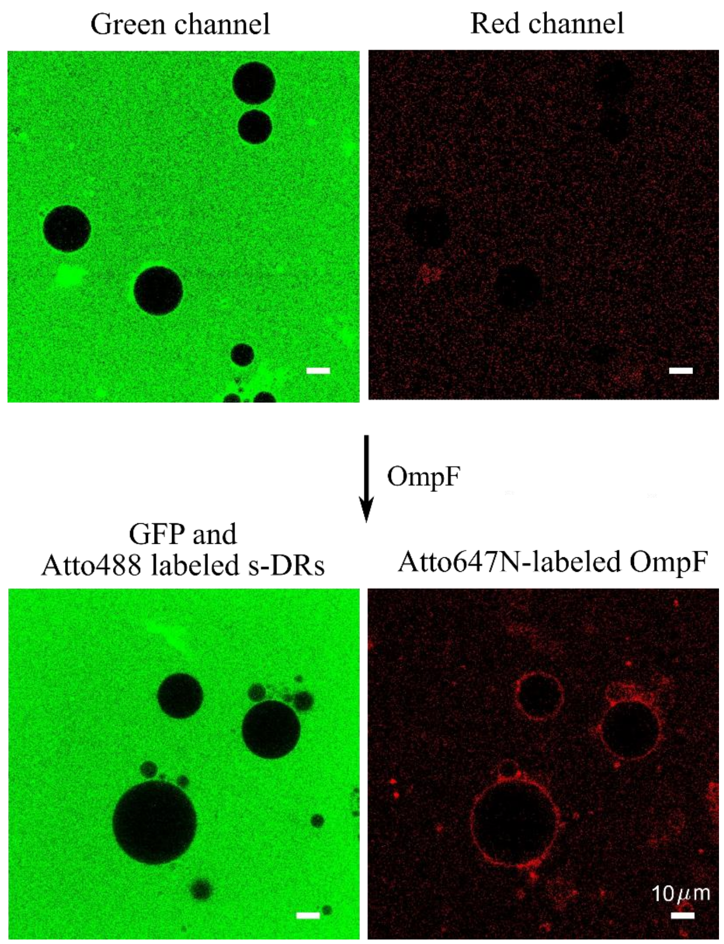

Fig. S33. s-DR-bound GUVs. In the presence of OmpF, no GFP influx is observed. Scale bars: 10 μm.

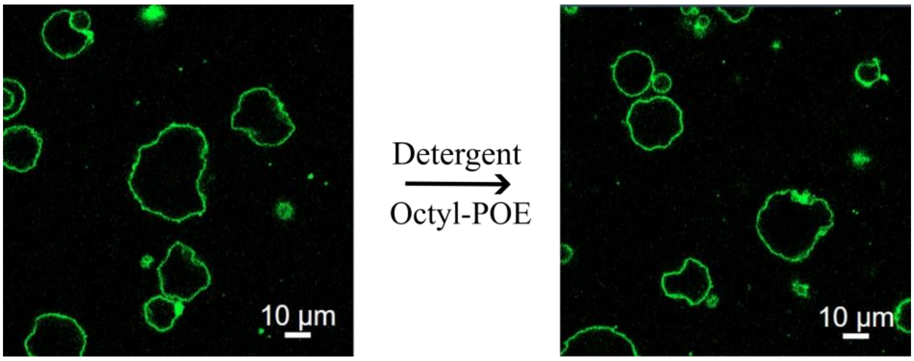

Fig. S34. Influence of detergent Octyl-POE on the membrane recovery process. Incubation of detergent Octyl-POE with the e-DR-bound deformed GUVs does not release the GUV deformations. The detergent concentration was the same as that used for the addition of OmpF.

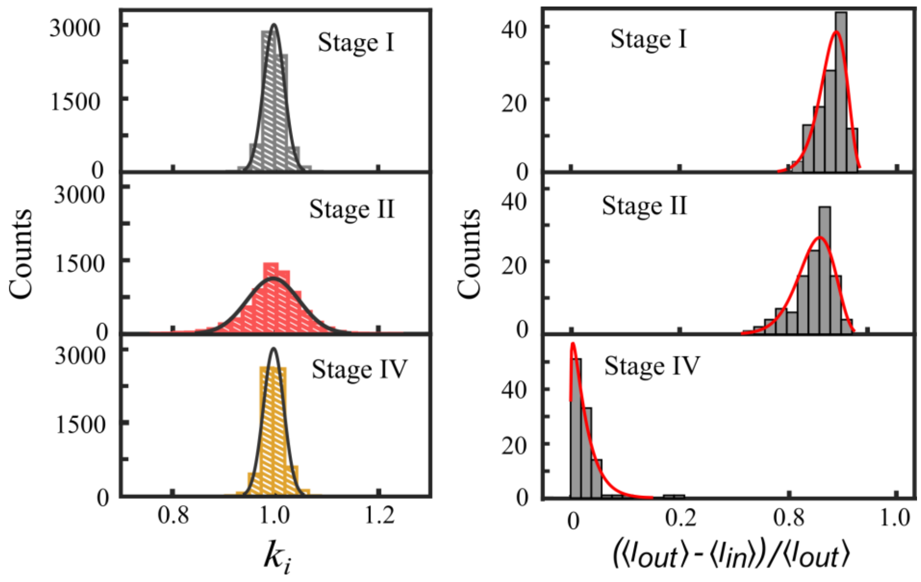

Fig. S35. Statistics of k_i and the normalized fluorescence intensity difference of GUVs by analyzing n>100 GUVs per stage from three independent experiments.

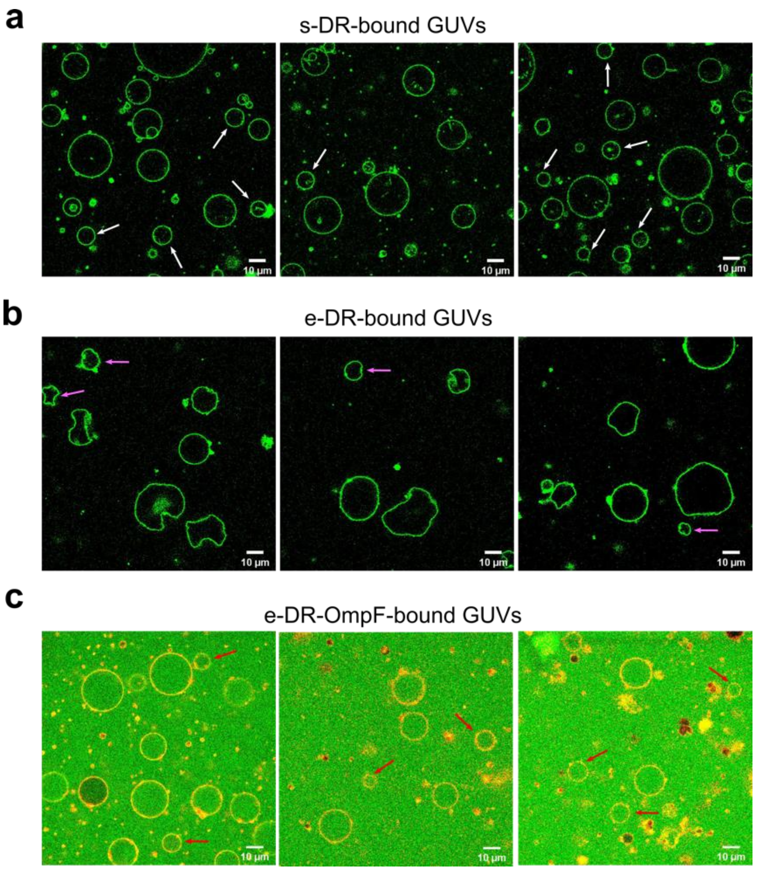

Fig. S36. For both small GUVs (<10 μm, white arrows) and large GUVs (>10 μm, pink arrows), they all undergo the process of membrane deformations, GUV shape recovery, synthetic channel formation, and cargo transport across the membranes. (**a**) s-DR-bound GUVs. (**b**) e-DR-bound GUVs. (**c**) e-DR-OmpF-bound GUVs.

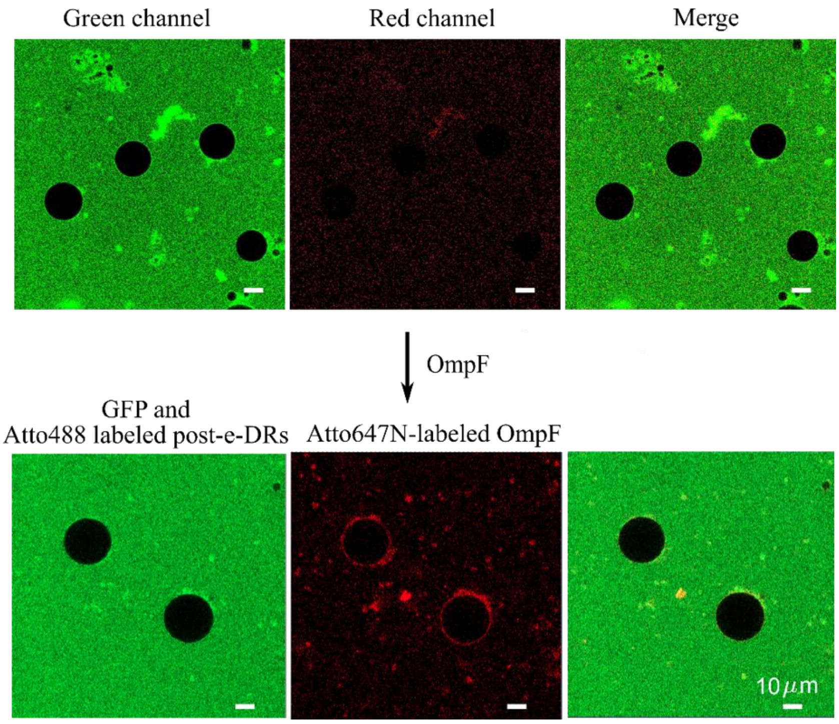

Fig. S37. Post-e-DR-bound GUVs without deformations. In the presence of OmpF, no GFP influx is observed. Scale bars: 10 μm.

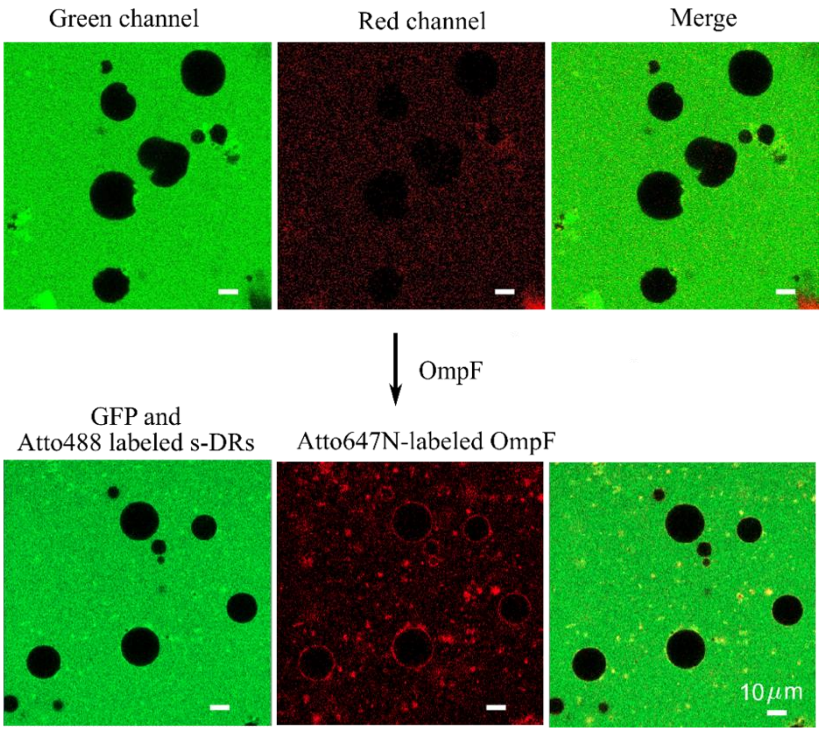

Fig. S38. s-DR-bound deformed GUVs. GUVs are deformed by binding of the s-DRs with a surface density of ~ 100 μm^{-2} (Fig. S26 (iv)). In the presence of OmpF, no GFP influx is observed after the shape recovery of the GUVs. Scale bars: 10 μm.

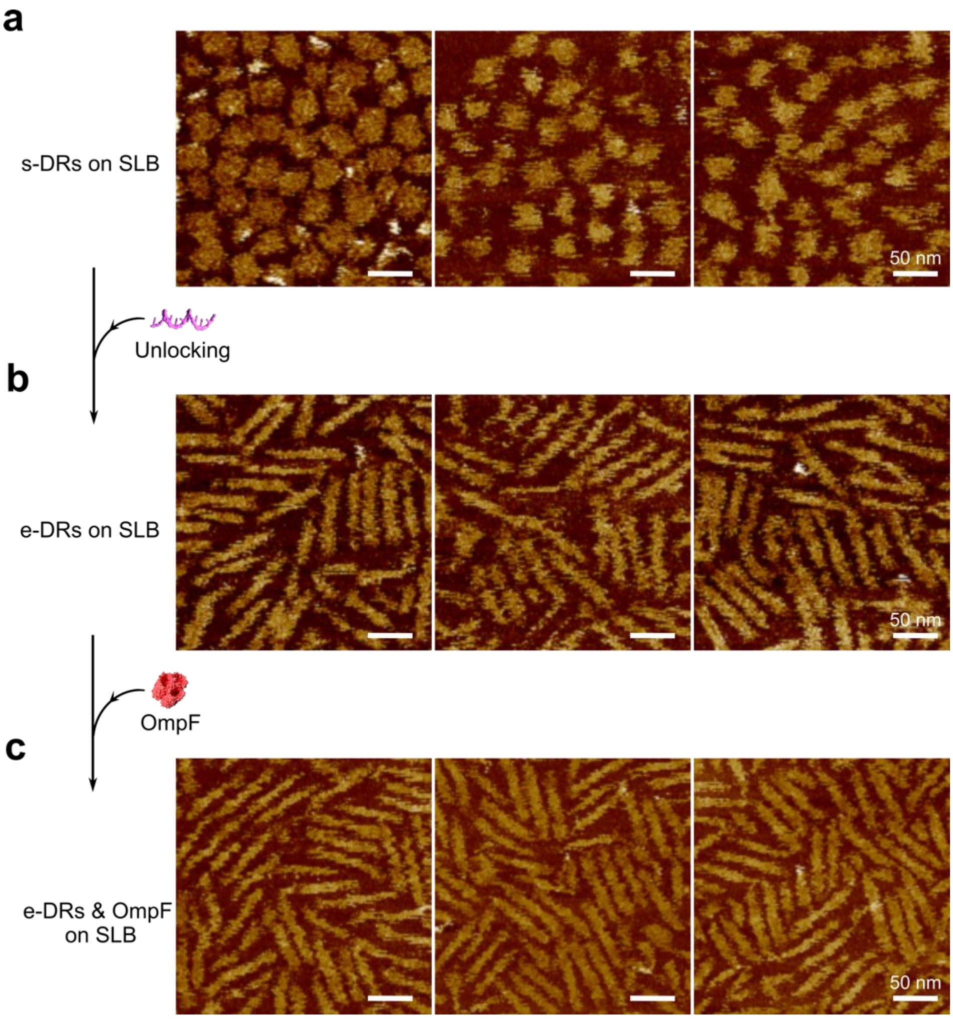

Fig. S39. Combined system of the DRs and OmpF on a 2D SLB characterized by AFM. (**a**) s-DRs are first bound to a 2D SLB, displaying a disordered arrangement on the membrane. (**b**) Addition of unlocking strands triggers the transformation from the s-DRs to e-DRs. The e-DRs tend to align side by side through rearrangements on the membrane, creating local order. (**c**) Subsequent addition of OmpF does not affect the distribution of the e-DRs on the membrane, and no synthetic channels are visible in the AFM images. Scale bars: 50 nm.

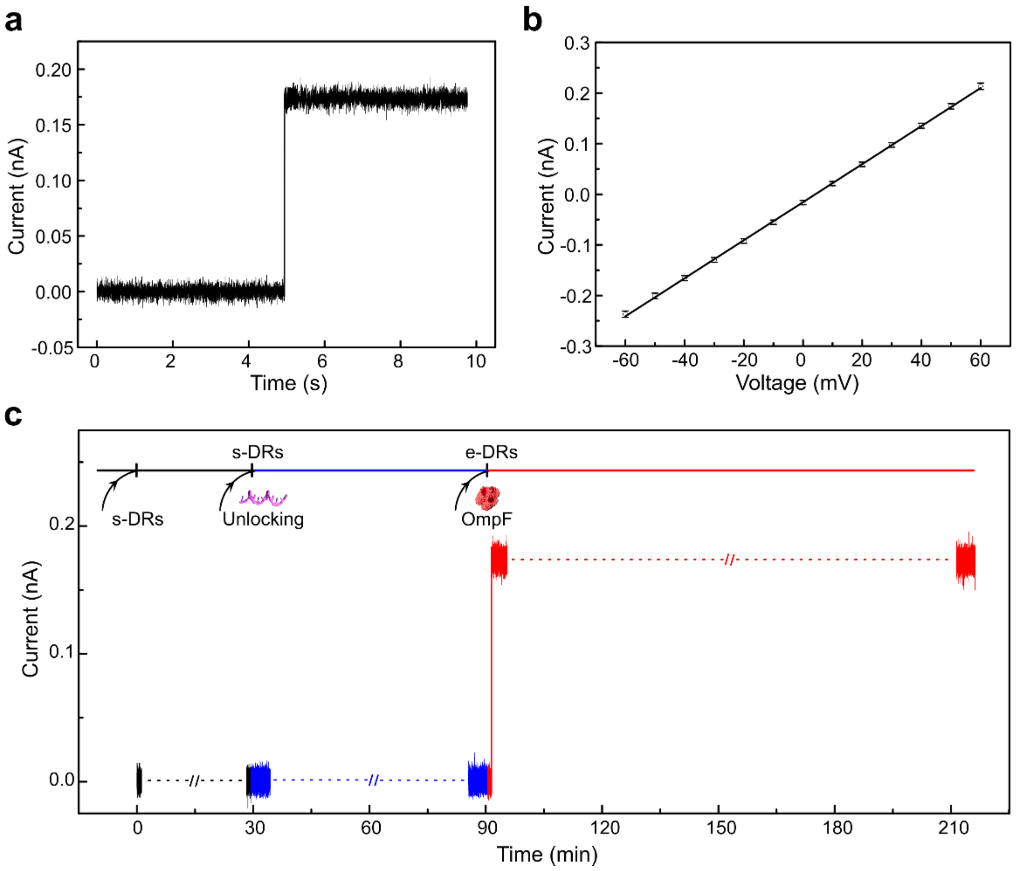

Fig. S40. (**a**) Current trace showing a single OmpF (1 M KCl, 20 mM HEPES, pH 7.4) inserted into a planar lipid membrane, recorded at an applied voltage of +50 mV. (**b**) Average current–voltage graph of OmpF for voltages ranging from −60 mV to +60 mV in steps of 10 mV. Data represent mean ± SEM (n = 3). (**c**) Current traces of the s-DR binding to the membrane (0-30 min, 25 °C) and transformation of the s-DRs to e-DRs (30-90 min, 40 °C) by the addition of unlocking strands, recorded at an applied voltage of +50 mV. No channel-forming currents are detectable. Then, OmpF is introduced into the system. The immediate current detection indicates the insertion of OmpF into the membrane. The current persists for up to 2 hours without any observable changes (90-210 min, 25 °C), confirming the absence of synthetic channel formation. The measurements are performed on 4-cavity microelectrode cavity array (MECA) chips (Ionera, Germany) using an Orbit Mini platform (Nanion Technologies, Germany).

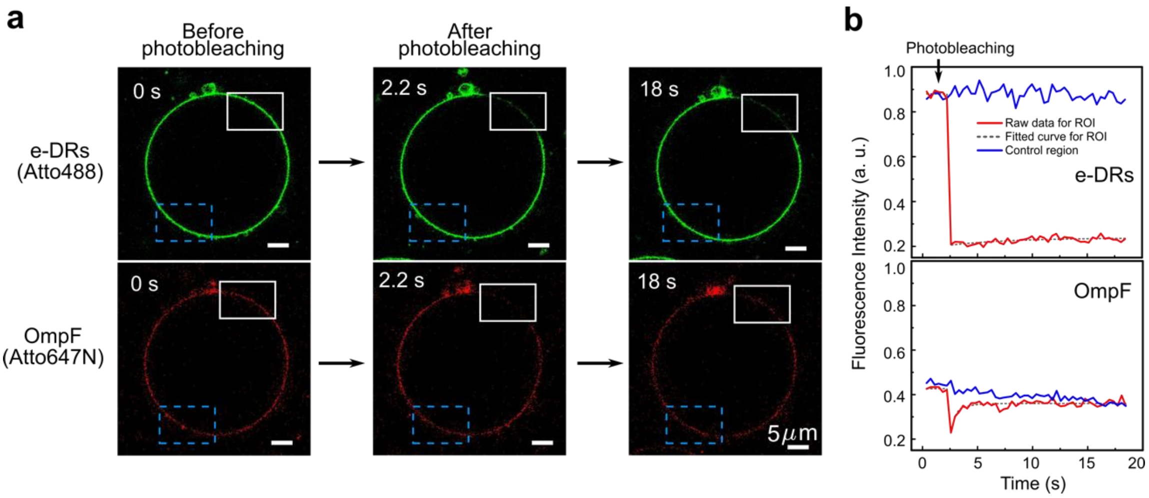

Fig. S41. FRAP measurements of the e-DR-OmpF-GUV system. (**a**) Confocal images of a single GUV before photobleaching (left), 2.2 s after photobleaching (middle) and 18 s after photobleaching (right). The e-DRs and OmpF are labeled with Atto488 and Atto647N, respectively. The laser wavelengths for photobleaching of Atto488 and Atto647N are 488 nm and 639 nm, respectively. The photobleached area (region of interest, ROI) is highlighted by the white box. The dashed blue box represents a control region without photobleaching. (**b**) Fluorescence intensity recovery after photobleaching over time. Top: after FRAP, the fluorescence signal of the Atto488-labeled e-DRs is not recovered (red). The fluorescence signal of the control region remains nearly unchanged (blue). Bottom: after FRAP, the fluorescence signal of the Atto647N-labeled OmpF is recovered to the same level (red) as that of the control region (blue). The slight intensity decrease (both red and blue) results from the fluorescence quenching of the relatively weaker Atto647N signal (compared to that of Atto488) during the image data acquisition process. Scale bars:5 μm.

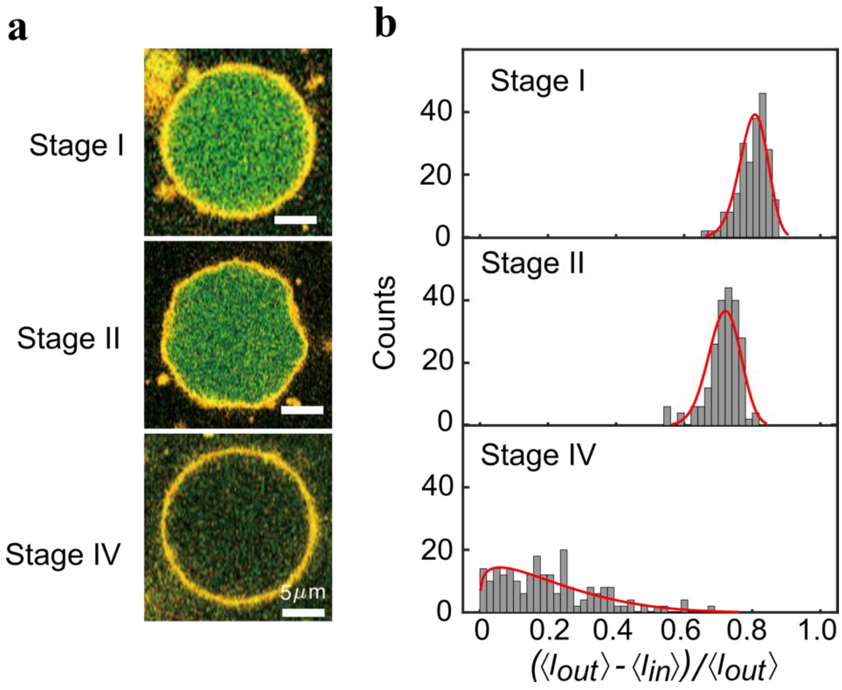

Fig. S42. Formation of the synthetic channels is confirmed also by the efflux experiment. Data obtained from three independent experiments. Scale bars: 5 μm.

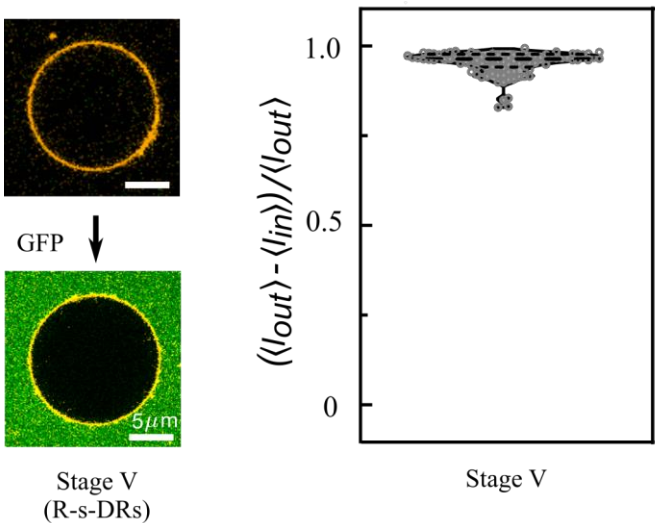

Fig. S43. Closure of the synthetic channels at stage V results in a very low GFP influx. Left: confocal images of the R-s-DR bound GUV before (top) and after (bottom) the addition of GFP. Orange: Cy3 labeled R-s-DRs. Green: GFP. Yellow: merged signals of Cy3 and GFP. Right: violin plot of the normalized fluorescence intensity difference of GUVs by analyzing n= 91 GUVs.

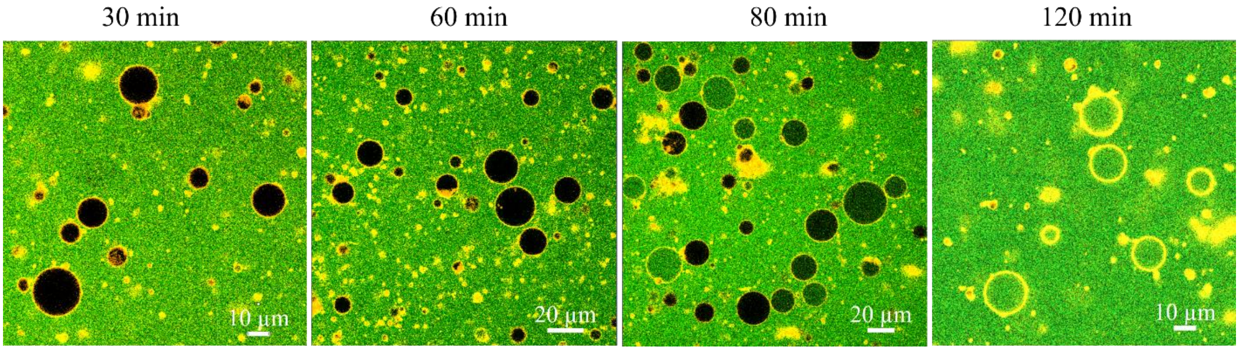

Fig. S44. Inhibition of the membrane perforation and closure of the synthetic channels by driving the transformation from the e-DRs to R-s-DRs at different time intervals from stage II. Yellow: merged signals of Cy3 and GFP. Green: GFP.

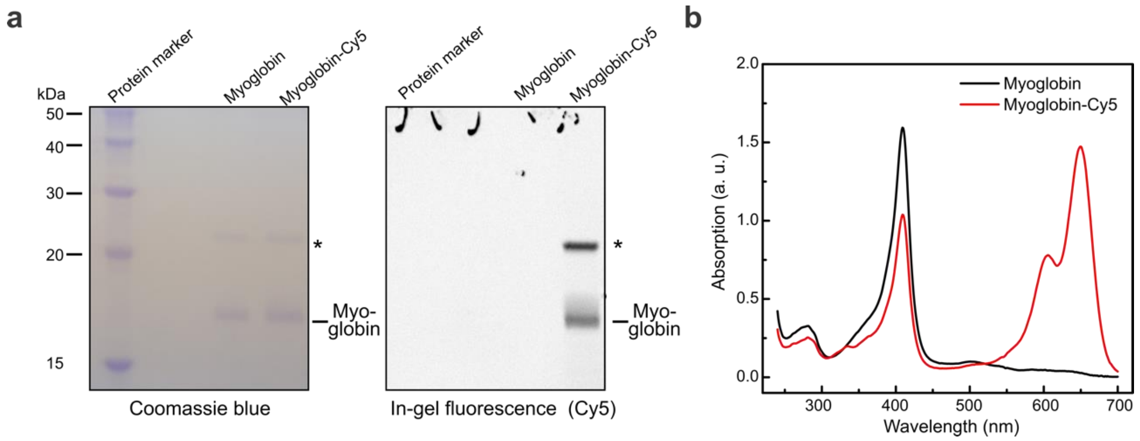

Fig. S45. Analysis of myoglobin labeled by fluorescent Cy5. (**a**) SDS-PAGE of non-labelled and Cy5-labeled myoglobin (~ 0.2 μg each). Left, coomassie blue staining; right, in-gel fluorescence (Cy5). *Unknown combination protein. (**b**) UV−vis absorbance spectra of myoglobin and myoglobin-Cy5. The absorption peaks around 418 nm and 650 nm are attributed to myoglobin and Cy5, respectively, indicating the successful Cy5 labeling of myoglobin.

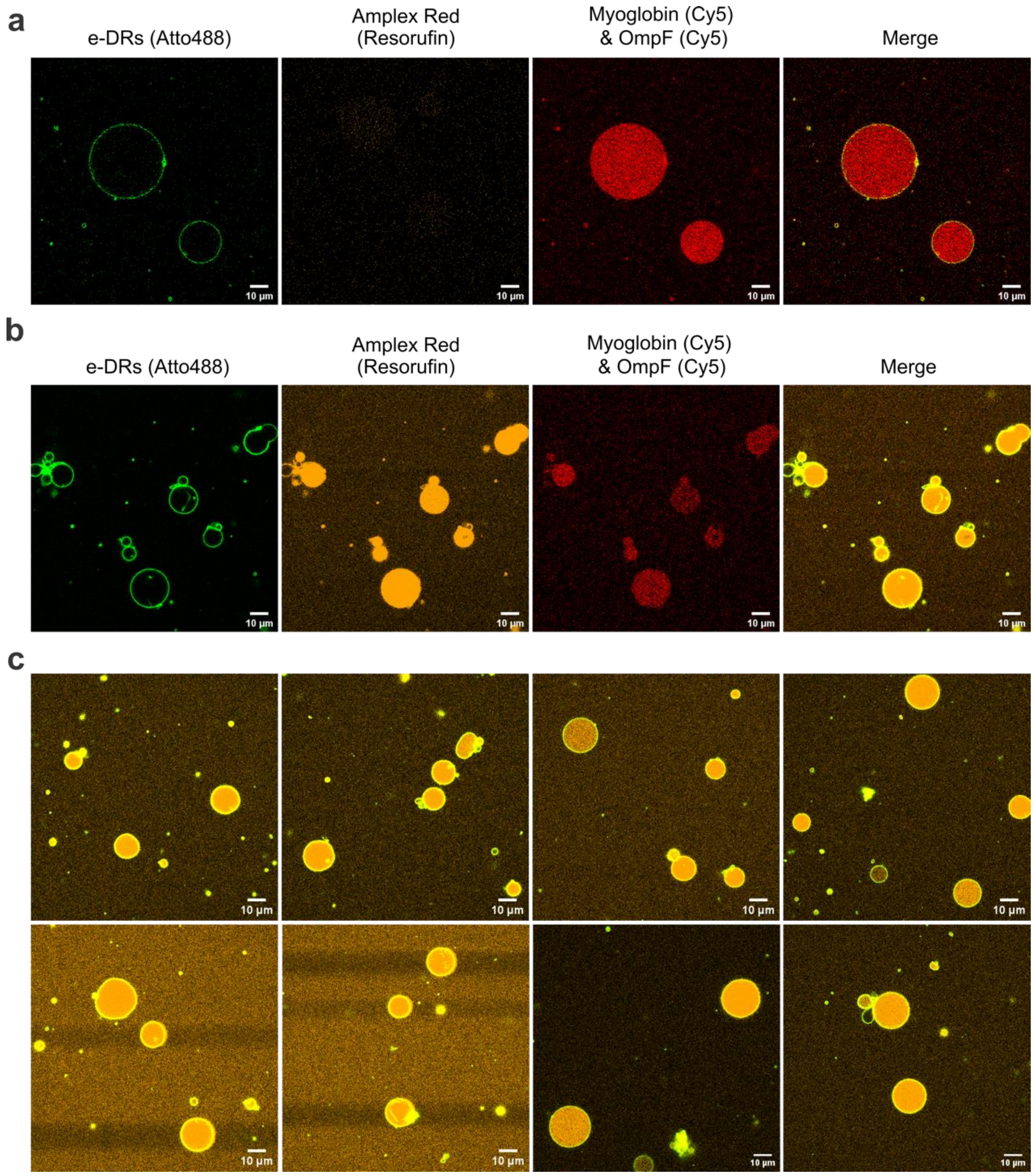

Fig. S46. Confocal images of different channels in the absence (**a**) and presence (**b**) of glucose influx via OmpF inhibits and promotes the glucose oxidase (GOx)-myoglobin enzyme cascade reactions, respectively. (**c**) Overview confocal images of the GOx-myoglobin enzyme cascade reactions in the presence of glucose, demonstrating system reproducibility.

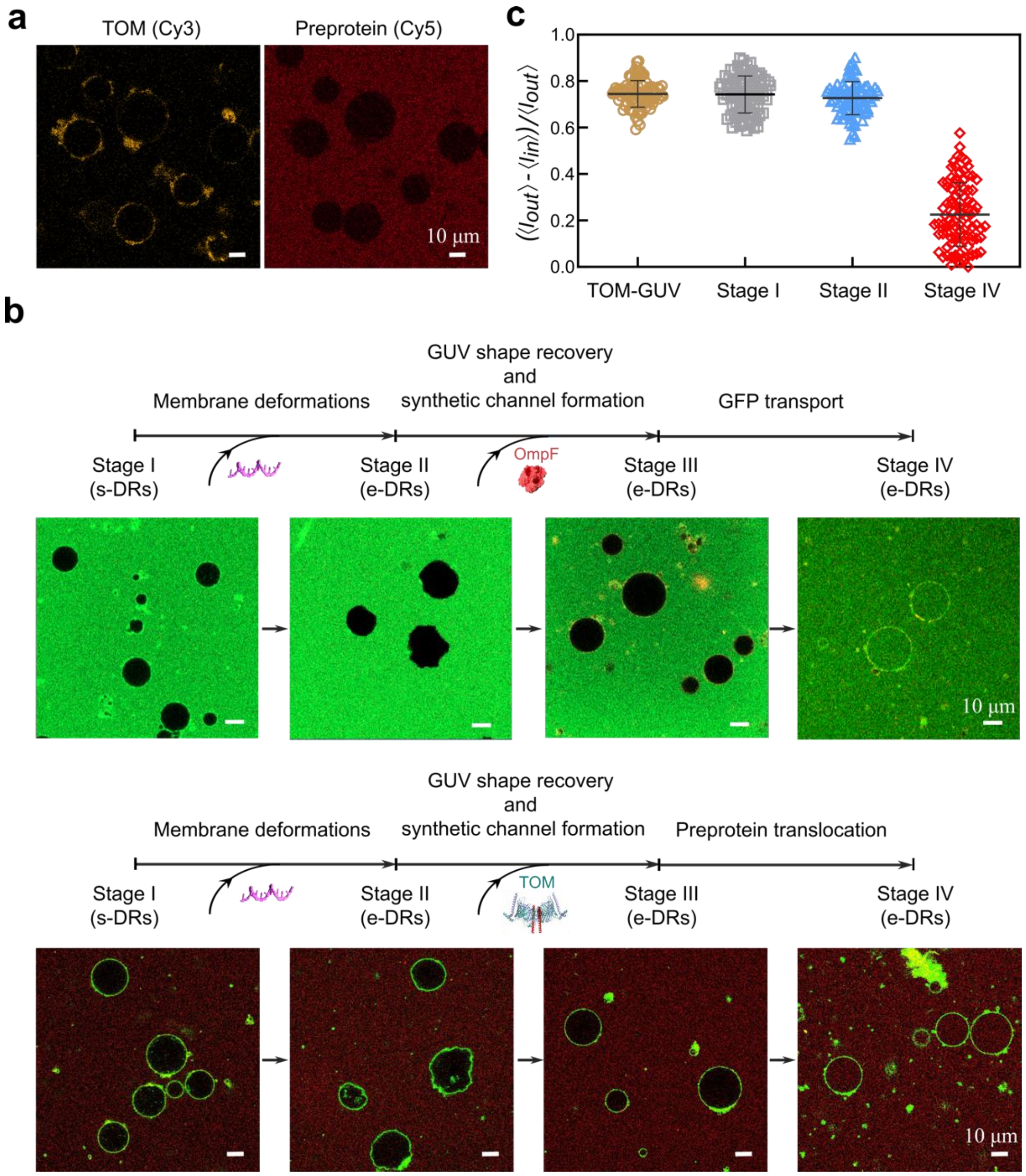

Fig. S47. Formation of synthetic channels by the DNA raft architectures. (**a**) TOM core complex containing a 6×His tag at subunit Tom22 was isolated and fluorescently labelled as previously described [9,10,11]. Left: reconstitution of preprotein translocase of the outer membrane of mitochondria (TOM core complex, pore diameter ~2.5 nm, Cy3-labeled) into the GUV membranes. Right: after the successful reconstitution of TOM (~ 300 nM), mitochondrial preproteins (Su9-MBP, ~40 kDa, courtesy of Pamela Ornelas, Max Planck Institute of Biophysics, Frankfurt, Cy5-labeled) is added into the extracellular space. The Su9-MBP was covalently labelled with the fluorescent dye Cy5-maleimide according to the reported method [11]. (**b**) Top: Fig. 3a from the main text. Bottom*:* Time course of the Su9-MBP influx in the hybrid DNA raft-TOM-GUV system. It shows that the new system with TOM exhibits the same set of phenomena as OmpF (Fig. 3a), namely, membrane deformation, GUV shape recovery, synthetic channel formation and cargo transport across the membranes. (**c**) Statistics of the fluorescence intensity difference $(\langle I_{out}\rangle - \langle I_{in}\rangle)/\langle I_{out}\rangle$ for TOM-GUVs (n = 87), DNA raft-TOM-GUVs at stages I (n = 82), II (n = 83), and IV (n = 96), accordingly. No evident influx of Su9-MBP is observed in the case of bare GUVs with TOM reconstitution (brown), at stage I (grey), or at stage II (blue), whereas obvious transport of Su9-MBP across the GUV

membranes is detected at stage IV (red). Data represent mean ± SD from three independent experiments. Scale bars: 10 μm.

Table S1. Comparison of the GUV deformation efficiencies by the 12d Chol DRs at three different states on GUVs with a surface density of ~ 36 μm^{-2} and different osmotic pressures (Supplementary Data S1 to S9).

**Surface density: ~ 36 μm^{-2}**	Hypo ($\Pi_{in} > \Pi_{out}$)	Iso ($\Pi_{in} = \Pi_{out}$)	Hyper ($\Pi_{in} < \Pi_{out}$)
s-DR-bound GUVs	1.3% (n=106)	2.8% (n=106)	4.4% (n=113)
e-DR-bound GUVs	3.8% (n=106)	2.7% (n=110)	1.9% (n=107)
R-s-DR-bound GUVs	3.7% (n=109)	3.7% (n=107)	0 (n=106)

Table S2. Comparison of the GUV deformation efficiencies by the 12d Chol DRs at three different states on GUVs with a surface density of ~ 60 μm^{-2} and different osmotic pressures (Supplementary Data S10 to S15).

**Surface density: ~ 60 μm^{-2}**	Hypo ($\Pi_{in} > \Pi_{out}$)	Iso ($\Pi_{in} = \Pi_{out}$)	Hyper ($\Pi_{in} < \Pi_{out}$)
s-DR-bound GUVs	1.7% (n=119)	11.2% (n=152)	10% (n=110)
e-DR-bound GUVs	41.1% (n=107)	72.4% (n=152)	80% (n=110)
R-s-DR-bound GUVs	7.5% (n=106)	17.8% (n=152)	11.1% (n=108)

Table S3. Comparison of the GUV deformation efficiencies by the 12d Chol DRs at three different states on GUVs with a surface density of ~ 83 μm^{-2} and different osmotic pressures (Supplementary Data S16 to S24).

**Surface density: ~ 83 μm^{-2}**	Hypo ($\Pi_{in} > \Pi_{out}$)	Iso ($\Pi_{in} = \Pi_{out}$)	Hyper ($\Pi_{in} < \Pi_{out}$)
s-DR-bound GUVs	30.4% (n=115)	29.1% (n=117)	30.7% (n=114)
e-DR-bound GUVs	73.1% (n=108)	69.8% (n=106)	75.5% (n=106)
R-s-DR-bound GUVs	38.7% (n=106)	26.4% (n=106)	39.1% (n=110)

Table S4. Comparison of the GUV deformation efficiencies by the 12d Chol DRs at three different states on GUVs with a surface density of ~ 100 μm^{-2} and different osmotic pressures (Supplementary Data S25 to S33).

**Surface density: ~ 100 μm^{-2}**	Hypo ($\Pi_{in} > \Pi_{out}$)	Iso ($\Pi_{in} = \Pi_{out}$)	Hyper ($\Pi_{in} < \Pi_{out}$)
s-DR-bound GUVs	31.7% (n=120)	22.3% (n=130)	40.3% (n=119)
e-DR-bound GUVs	74.4% (n=117)	75.9% (n=108)	92.6 % (n=149)
R-s-DR-bound GUVs	44 % (n=116)	31.1% (n=119)	47.3% (n=110)

Table S5 Summary of the experimental configurations for the formation of synthetic channels.

**Membrane system**	**DNA origami rafts**	**Protein pore**	**Formation of synthetic channels**	**Supported results**
3D GUV	s-DRs		×	Fig. S32a<br>Fig. S38
3D GUV	post-e-DRs		×	Fig. S37
3D GUV	s-DRs → e-DRs		×	Fig. S32b
3D GUV	s-DRs	OmpF	×	Fig. S33
3D GUV	post-e-DRs	OmpF	×	Fig. S37
3D GUV	s-DRs → e-DRs	OmpF	√	Fig. 3a<br>Fig. S32d
3D GUV	s-DRs → e-DRs	TOM	√	Fig. S47
2D planar membrane	s-DRs		×	Fig. S39a<br>Fig. S40c
2D planar membrane	s-DRs → e-DRs		×	Fig. S39b<br>Fig. S40c
2D planar membrane	s-DRs → e-DRs	OmpF	×	Fig. S39b<br>Fig. S40c

Table S6. Sequences of the s-DRs.

**Name**	**Sequence (5' - 3')**	**5' End**
1	AACGCCATCAAAAGTATAAGCAAATA	1[40]
2	GGCCTTCCTGTAGTGCTGCAAGGCGA	1[56]
3	ACATTAAATGTGAAATCATATGTACC	1[72]
4	CGTCGGATTCTCCGGCGATCGGTGCG	1[88]
5	GCGGATTGACCGTGTCTGGAGCAAAC	1[104]
6	ACGTTGGTGTAGAGGAAACCAGGCAA	1[120]

Name	Sequence (5' - 3')	5' End
7	ACCGTGCATCTGCAGGGTAGCTATTT	1[136]
8	TTTAAATTGTAAATCAGAGCATAAAG	2[39]
9	AAACAGGAAGATTATAATTCGCGTCT	2[55]
10	CCGGTTGATAATCCTTTTGCGGGAGA	2[71]
11	AAACTAGCATGTCGCGAGTAACAACC	2[87]
12	AAGAGAATCGATGCTCATATATTTTA	2[103]
13	TCATTGCCTGAGAAATGGGATAGGTC	2[119]
14	TTGAGAGATCTACGGGTGAGAAAGGC	2[135]
15	CTAAATCGGTTGTCTACTAATAGTAG	3[40]
16	TGACCCTGTAATAAGAAAAGCCCCAA	3[56]
17	AGCCTTTATTTCACTGTTTAGCTATA	3[72]
18	AATTTTTAGAACCAACGGTAATCGTA	3[88]
19	AATGCAATGCCTGAACGAGTAGATTT	3[104]
20	TAAAGATTCAAAAAAAGGCTATCAGG	3[120]
21	CGGAGACAGTCAAGTACGGTGTCTGG	3[136]
22	TAGCATTAACATCTCAGAAGCAAAGC	4[39]
23	GGTGGCATCAATTACCAAAAACATTA	4[55]
24	TTTTCATTTGGGGTCAAATATCGCGT	4[71]
25	AATGGTCAATAACACGCAAGGATAAA	4[87]
26	AGTTTGACCATTACTCCAACAGGTCA	4[103]
27	TCCCAATTCTGCGAGTAATGTGTAGG	4[119]
28	AAGTTTCATTCCAGTCATTTTTGCGG	4[135]
29	GGATTGCATCAAACCCTCAAATGCTT	5[40]
30	AGCCCGAAAGACTCGCGAGCTGAAAA	5[56]
31	TTTAATTCGAGCTAGACTGGATAGCG	5[72]
32	GACCGGAAGCAAAGATACATTTCGCA	5[88]
33	GGATTAGAGAGTAGAGAGGCTTTTGC	5[104]
34	CTTTTGATAAGAGTATAACAGTTGAT	5[120]
35	ATGGCTTAGAGCTGCAACACTATCAT	5[136]
36	TAAACAGTTCAGATTGGGCTTGAGAT	6[39]
37	ATTCATTGAATCCAAGATTAAGAGGA	6[55]
38	TCCAATACTGCGGCGATTTTAAGAAC	6[71]
39	TAGTAAAATGTTTTCAAAGCGAACCA	6[87]
40	AAAAGAAGTTTTGTACGTTAATAAAA	6[103]
41	AAACCAAAATAGCCCTTTAATTGCTC	6[119]
42	AACCCTCGTTTACATCAGTTGAGATT	6[135]
43	GGTTTAATTTCAAACCCAAATCAACG	7[40]
44	GAATTACCTTATGAATCGTCATAAAT	7[56]
45	TGGCTCATTATACAGGCTGGCTGACC	7[72]
46	GGGAAGAAAAATCCCAGAGGGGGTAA	7[88]
47	CGAACTAACGGAAAACTGACCAACTT	7[104]
48	GGTAGAAAGATTCCAGACGACGATAA	7[120]
49	TAGGAATACCACATCCATGTTACTTA	7[136]
50	TAACAAAGCTGCTGGGTAGCAACGGC	8[39]
51	CCGGATATTCATTCTTTAATCATTGT	8[55]
52	TTCATCAAGAGTAGTTTCCATTAAAC	8[71]
53	AGACCAGGCGCATCAGTCAGGACGTT	8[87]
54	TGAAAGAGGACAGAAACGAAAGAGGC	8[103]
55	CATAAGGGAACCGCAACATTATTACA	8[119]

Name	Sequence (5' - 3')	5' End
56	GCCGGAACGAGGCATTATACCAAGCG	8[135]
57	TACAGAGGCTTTGTCGCTGAGGCTTG	9[40]
58	TTTTCATGAGGAAATCTTGACAAGAA	9[56]
59	GGGTAAAATACGTGTTGCGCCGACAA	9[72]
60	AGGCACCAACCTAATGAACGGTGTAC	9[88]
61	AAAAGAATACACTGGTTTATCAGCTT	9[104]
62	TTGACCCCCAGCGGCAGACGGTCAAT	9[120]
63	CGAAACAAAGTACCACGTTGAAAATC	9[136]
64	CAGGGAGTTAAAGCCCAATAGGAACC	10[39]
65	CCGATATATTCGGAGGACTAAAGACT	10[55]
66	TGACAACAACCATACAACGCCTGTAG	10[71]
67	CTTGATACCGATAAATGCCACTACGA	10[87]
68	GCTTTCGAGGTGAAAGTTTTGTCGTC	10[103]
69	CTTTAATTGTATCAAAACACTCATCT	10[119]
70	TCCAAAAAAAAAGGTGCTAAACAACTT	10[135]
71	CATGTACCGTAACAGTACCGCCACCC	11[40]
72	ACCAGTACAAACTCGCCCACGCATAA	11[56]
73	CATTCCACAGACAGGTTGATATAAGT	11[72]
74	GCGTAACGATCTAATTTCTTAAACAG	11[88]
75	TTTCCAGACGTTATTAGGATTAGCGG	11[104]
76	CTGTATGGGATTTCTCCAAAAGGAGC	11[120]
77	TCAACAGTTTCAGATTATTCTGAAAC	11[136]
78	TCAGAACCGCCACAGCATTGACAGGA	12[39]
79	ACTCAGGAGGTTTACTGAGTTTCGTC	12[55]
80	ATAGCCCGGAATAACAAATAAATCCT	12[71]
81	GTGCCGTCGAGAGGCCCTCATAGTTA	12[87]
82	GGTTTTGCTCAGTCGTTCCAGTAAGC	12[103]
83	CTCAAGAGAAGGAGTAAATGAATTTT	12[119]
84	ATGAAAGTATTAAATAAGTTTTAACG	12[135]
85	GGTTGAGGCAGGTGCCGCCACCCTCA	13[40]
86	TTGATATTCACAAGGTGTATCACCGT	13[56]
87	CATTAAAGCCAGACATAATCAAAATC	13[72]
88	TCTCTGAATTTACACCAGGCGGATAA	13[88]
89	GTCATACATGGCTTTTTCATCGGCAT	13[104]
90	GAGTGTACTGGTAGAGGCTGAGACTC	13[120]
91	GGGTCAGTGCCTTGTAATCAGTAGCG	13[136]
92	GAACCGCCACCCTTTTGTCACAATCA	14[39]
93	CGCCTCCCTCAGACAGACGATTGGCC	14[55]
94	ACCGGAACCAGAGGGGCGACATTCAA	14[71]
95	TTTGCCATCTTTTATGGAAAGCGCAG	14[87]
96	TTTCGGTCATAGCTCATTAAAGGTGA	14[103]
97	AGACTGTAGCGCGTTTGATGATACAG	14[119]
98	ACAGAATCAAGTTGCCAGCAAAATCA	14[135]
99	ATAGAAAATTCATGTTAGCAAACGTA	15[40]
100	GCCAAAGACAAAACCACCACCGGAAC	15[56]
101	CCGATTGAGGGAGAACGGAATACCCA	15[72]
102	TGACGGAAATTATCCCCTTATTAGCG	15[88]
103	ATTATCACCGTCAAAGCAGATAGCCG	15[104]
104	TTTGGGAATTAGATGCCTTTAGCGTC	15[120]

Name	Sequence (5' - 3')	5' End
105	CCAGTAGCACCATGAAACAATGAAAT	15[136]
106	GAAAATACATACAGCCAGTTACAAAA	16[39]
107	TATTACGCAGTATATGGTTTACCAGC	16[55]
108	AAAGAACTGGCATGATTTTTTGTTTA	16[71]
109	GAAACGCAATAATGGAAGGTAAATAT	16[87]
110	AACAAAGTTACCAACATAAAAACAGG	16[103]
111	TTTTAAGAAAAGTCCGACTTGAGCCA	16[119]
112	AGCAATAGCTATCCAAAGTCAGAGGG	16[135]
113	TAAACAGCCATATAGTTGCTATTTTG	17[40]
114	CCAAATAAGAAACGATTAAGACTCCT	17[56]
115	ACGTCAAAAATGATTTTAGCGAACCT	17[72]
116	TACAGAGAGAATAGAAGGAAACCGAG	17[88]
117	GAAGCGCATTAGAGCAAGCAAATCAG	17[104]
118	TGAACACCCTGAATTACCGAAGCCCT	17[120]
119	TAATTGAGCGCTAAACAAGCAAGCCG	17[136]
120	CACCCAGCTACAAAATAAGAGAATAT	18[39]
121	TTAAATCAAGATTTATTTATCCCAAT	18[55]
122	CCCGACTTGCGGGCGACAATAAACAA	18[71]
123	AGAACGCGAGGCGAAATAGCAGCCTT	18[87]
124	ATATAGAAGGCTTTAGATAAGTCCTG	18[103]
125	ACCGCGCCCAATACGGGAGAATTAAC	18[119]
126	TTTTTATTTTCATTAGAAACCAATCA	18[135]
127	AAAGTACCGACAAAACAGTAGGGCTT	19[40]
128	TCTGTCCAGACGAAGGTTTTGAAGCC	19[56]
129	CATGTTCAGCTAAGTTTAGTATCATA	19[72]
130	TGTTTATCAACAAATCCGGTATTCTA	19[88]
131	AACAAGAAAAATATAAGGCGTTAAAT	19[104]
132	ATTTACGAGCATGCGTAGGAATCATT	19[120]
133	ATAATCGGCTGTCTTTCATCTTCTGA	19[136]
134	AATTGAGAATCGCCTGTAAATCGTCG	20[39]
135	AAAGCCAACGCTCAAGGTAAAGTAAT	20[55]
136	TGCGTTATACAAACGATAGCTTAGAT	20[71]
137	CTAGAAAAAGCCTTGCAGAACGCGCC	20[87]
138	AAGAATAAACACCCATAGGTCTGAGA	20[103]
139	ACCGTGTGATAAAATATCCCATCCTA	20[119]
140	CCTAAATTTAATGACTATATGTAAAT	20[135]
141	CTATTAATTAATTTTTAACAATTTCA	21[40]
142	CTTGAAAACATAGTTCTTACCAGTAT	21[56]
143	TAAGACGCTGAGACTGAGCAAAAGAA	21[72]
144	AATTTATCAAAATGGAATCATAATTA	21[88]
145	GACTACCTTTTTATTGAATACCAAGT	21[104]
146	GTTGGGTTATATAGTTTGAAATACCG	21[120]
147	GCTGATGCAAATCGTAACAGTACCTT	21[136]
148	TTTGAATTACCTTTGGCCCTGAGAGA	22[39]
149	AAAATTAATTACATTCCCTTAGAATC	22[55]
150	GATGATGAAACAAGAAAATCCTGTTT	22[71]
151	TCATTTCAATTACAGAGTCAATAGTG	22[87]
152	TACAAAATCGCGCAAAGAATAGCCCG	22[103]
153	TCGCCTGATTGCTACCTCCGGCTTAG	22[119]

Name	Sequence (5' - 3')	5' End
154	TTACATCGGGAGACACTATTAAAGAA	22[135]

Table S7. Sequences of unlocking DNA strands to reconfigure the s-DRs to e-DRs.

Name	Sequence (5'- 3'), Unlocking DNA strands	5' End
1	TAATACTAGATTAAAATTCGCATTAAATTTTTGTTAAATCAGCTCATTTTGTAAAACGACGG	2[23]
2	AATTCTAGCAATTAATGCCGGAGCAGTTTGAGGGGACGACGACAGTATCGGCCTCAGGAAGA	2[151]
3	ATCCTTATATAGGCAAGGCAAAGAATTAGCAAAATTAAGCAATAAAGCCCGTTAATATTTTG	4[23]
4	TTAGCTATTAATATGCAACTAAAATCACCATCAATATGATATTCAACCGTTCTAGCTGATAA	4[151]
5	TTGACGATACCATAAATCAAAAATCAGGTCTTTACCCTGACTATTATAGCAATAAATCATAC	6[23]
6	GACAATTTGTAGGCATAGTAAGATAATTGCTGAATATAATGCTGTAGCTCAACATGTTTTAA	6[151]
7	GCTCCAAAAGAGGCTTGCCCTGACGAGAAACACCAGAACGAGTAGTAAAAAACGAGAATGAC	8[23]
8	GTTTGCCACTATCCGCGACCTGCTTCAACTAATGCAGATACATAACGCCAAAAGGAATTACG	8[151]
9	ACCACATGCGGATCGTCACCCTCAGCAGCGAAAGACAGCATCGGAACGACATTCAGTGAATA	10[23]
10	TTCTCTGTAGATAATAATTTTTTAACGGAGATTTGTATCATCGCCTGATAAATTGTGTCGAA	10[151]
11	AGTAGCATCCACCCTCAGAGCCACCACCCTCATTTTCAGGGATAGCAAGGCCGCTTTTGCGG	12[23]
12	TATGGACCCGTATTTCGGAACCTCGGAGTGAGAATAGAAAGGAACAACTAAAGGAATTGCGA	12[151]
13	CAACTGTAGACTCAGAGCCGCCACCAGAACCACCACCAGAGCCGCCGCCCCTCAGAACCGCC	14[23]
14	GTCTAGGACGCGATAGCAGCACCGAGTAACAGTGCCCGTATAAACAGTTAATGCCCCCTGCC	14[151]
15	TGAAGTGGACATATAAAAGAAACGCAAAGACACCACGGAATAAGTTTATCAGAGCCACCACC	16[23]
16	GAGGAACCTAATAATAAGAGCAATACCATTAGCAAGGCCGGAAACGTCACCAATGAAACCAT	16[151]
17	CCAGTCAATACTTACCAACGCTAACGAGCGTCTTTCCAGAGCCTAATTTTAAAGGTGGCAAC	18[23]
18	CACGATAGGGCGCACTCATCGAGATATCAGAGAGATAACCCACAAGAATTGAGTTAAGCCCA	18[151]
19	TCCAAGCGATGCCAACATGTAATTTAGGCAGAGGCATTTTCGAGCCAGTTTTTATCCTGAAT	20[23]
20	CCTTTAAGCAATATTTTAGTTAATTTCCTTATCATTCCAAGAACGGGTATTAAACCAAGTAC	20[151]
21	AAAGAGCTCGAGTACATAAATCAATATATGTGAGTGAATAACCTTGCTTCATATTTAACAAC	22[23]
22	CCTTTAAGCAAGATGAATATACACAATCGCAAGACAAAGAACGCGAGAAAACTTTTTCAAAT	22[151]

Table S8. Releasing DNA strands with toehold triggers and square - lock DNA strands (these two types of DNA strands are called together as Locking DNA strands) to reconfigure the e-DRs back to R-s-DRs.

Name	Sequence (5'- 3'), Locking DNA strands	5' End
	**Square - lock DNA strands**	
1	TTAAGTTGGGTAACGCCAGGGTTTTCCCAGTCACGACGTTTTAACCAATAGG	0[55]
2	GGCCTCTTCGCTATTACGCCAGCTGGCGAAAGGGGGATGCCAGCTTTCATCA	0[87]
3	AGCGCCATTCGCCATTCAGGCTGCGCAACTGTTGGGAAGGTGGGAACAAACG	0[119]
4	TCGCACTCCAGCCAGCTTTCCGGCACCGCTTCTGGTGCCTGGGCGCATCGTA	0[151]
5	ACCAGTGAGACGGGCAACAGCTGATTGCCCTTCACCGCCTTTTAATGGAAAC	23[8]
6	GTTGCAGCAAGCGGTCCACGCTGGTTTGCCCCAGCAGGCACATCAAGAAAAC	23[40]
7	GATGGTGGTTCCGAAATCGGCAAAATCCCTTATAAATCAAGAGGCGAATTAT	23[72]
8	AGATAGGGTTGAGTGTTGTTCCAGTTTGGAACAAGAGTCAACAATAACGGAT	23[104]
9	CGTGGACTCCAACGTCAAAGGGCGAAAAACCGTCTATCACAGGTTTAACGTC	23[136]
	**Releasing DNA strands with toehold triggers**	
23	CCGTCGTTTTACAAAATGAGCTGATTTAACAAAAATTTAATGCGAATTTTAATCTAGTATTA	
24	TCTTCCTGAGGCCGATACTGTCGTCGTCCCCTCAAACTGCTCCGGCATTAATTGCTAGAATT	
25	CAAAATATTAACGGGCTTTATTGCTTAATTTTGCTAATTCTTTGCCTTGCCTATATAAGGAT	

Name	Sequence (5'- 3'), Locking DNA strands	5' End
26	TTATCAGCTAGAACGGTTGAATATCATATTGATGGTGATTTTAGTTGCATATTAATAGCTAA	
27	GTATGATTTATTGCTATAATAGTCAGGGTAAAGACCTGATTTTTGATTTATGGTATCGTCAA	
28	TTAAAACATGTTGAGCTACAGCATTATATTCAGCAATTATCTTACTATGCCTACAAATTGTC	
29	GTCATTCTCGTTTTTTACTACTCGTTCTGGTGTTTCTCGTCAGGGCAAGCCTCTTTTGGAGC	
30	CGTAATTCCTTTTGGCGTTATGTATCTGCATTAGTTGAAGCAGGTCGCGGATAGTGGCAAAC	
31	TATTCACTGAATGTCGTTCCGATGCTGTCTTTCGCTGCTGAGGGTGACGATCCGCATGTGGT	
32	TTCGACACAATTTATCAGGCGATGATACAAATCTCCGTTAAAAAATTATTATCTACAGAGAA	
33	CCGCAAAAGCGGCCTTGCTATCCCTGAAAATGAGGGTGGTGGCTCTGAGGGTGGATGCTACT	
34	TCGCAATTCCTTTAGTTGTTCCTTTCTATTCTCACTCCGAGGTTCCGAAATACGGGTCCATA	
35	GGCGGTTCTGAGGGGCGGCGGCTCTGGTGGTGGTTCTGGTGGCGGCTCTGAGTCTACAGTTG	
36	GGCAGGGGGCATTAACTGTTTATACGGGCACTGTTACTCGGTGCTGCTATCGCGTCCTAGAC	
37	GGTGGTGGCTCTGATAAACTTATTCCGTGGTGTCTTTGCGTTTCTTTTATATGTCCACTTCA	
38	ATGGTTTCATTGGTGACGTTTCCGGCCTTGCTAATGGTATTGCTCTTATTATTAGGTTCCTC	
39	GTTGCCACCTTTAAAATTAGGCTCTGGAAAGACGCTCGTTAGCGTTGGTAAGTATTGACTGG	
40	TGGGCTTAACTCAATTCTTGTGGGTTATCTCTCTGATATCTCGATGAGTGCGCCCTATCGTG	
41	ATTCAGGATAAAAACTGGCTCGAAAATGCCTCTGCCTAAATTACATGTTGGCATCGCTTGGA	
42	GTACTTGGTTTAATACCCGTTCTTGGAATGATAAGGAAATTAACTAAAATATTGCTTAAAGG	
43	GTTGTTAAATATGAAGCAAGGTTATTCACTCACATATATTGATTTATGTACTCGAGCTCTTT	
44	ATTTGAAAAAGTTTTCTCGCGTTCTTTGTCTTGCGATTGTGTATATTCATCTTGCTTAAAGG	

Table S9. Staples with extension for hybridization with the TEG-Cholesterol modified DNA strands (cholesterol sites) on the DNA rafts.

Name	Sequence (5'—3')	5' End
Chol-ST	*Chol-TEG*-TTTTTAACCAGACCACCCATAGCAT	
FAM-Chol-ST	*Chol-TEG*-TTTTTAACCAGACCACCCATAGCAT-*FAM*	
4[39]-C	TAGCATTAACATCTCAGAAGCAAAGCTTTTATGCTATGGGTGGTCTGGTT	4[39]
4[71]-C	TTTTCATTTGGGGTCAAATATCGCGTTTTTATGCTATGGGTGGTCTGGTT	4[71]
4[103]-C	AGTTTGACCATTACTCCAACAGGTCATTTTATGCTATGGGTGGTCTGGTT	4[103]
4[135]-C	AAGTTTCATTCCAGTCATTTTTGCGGTTTTATGCTATGGGTGGTCTGGTT	4[135]
6[39]-C	TAAACAGTTCAGATTGGGCTTGAGATTTTTATGCTATGGGTGGTCTGGTT	6[39]
6[135]-C	AACCCTCGTTTACATCAGTTGAGATTTTTTATGCTATGGGTGGTCTGGTT	6[135]
10[39]-C	CAGGGAGTTAAAGCCCAATAGGAACCTTTTATGCTATGGGTGGTCTGGTT	10[39]
10[71]-C	TGACAACAACCATACAACGCCTGTAGTTTTATGCTATGGGTGGTCTGGTT	10[71]
10[103]-C	GCTTTCGAGGTGAAAGTTTTGTCGTCTTTTATGCTATGGGTGGTCTGGTT	10[103]
10[135]-C	TCCAAAAAAAAAGGTGCTAAACAACTTTTTTATGCTATGGGTGGTCTGGTT	10[135]
12[39]-C	TCAGAACCGCCACAGCATTGACAGGATTTTATGCTATGGGTGGTCTGGTT	12[39]
12[71]-C	ATAGCCCGGAATAACAAATAAATCCTTTTTATGCTATGGGTGGTCTGGTT	12[71]
12[103]-C	GGTTTTGCTCAGTCGTTCCAGTAAGCTTTTATGCTATGGGTGGTCTGGTT	12[103]
12[135]-C	ATGAAAGTATTAAATAAGTTTTAACGTTTTATGCTATGGGTGGTCTGGTT	12[135]
16[39]-C	GAAAATACATACAGCCAGTTACAAAATTTTATGCTATGGGTGGTCTGGTT	16[39]
16[71]-C	AAAGAACTGGCATGATTTTTTGTTTATTTTATGCTATGGGTGGTCTGGTT	16[71]
16[103]-C	AACAAAGTTACCAACATAAAAACAGGTTTTATGCTATGGGTGGTCTGGTT	16[103]
16[135]-C	AGCAATAGCTATCCAAAGTCAGAGGGTTTTATGCTATGGGTGGTCTGGTT	16[135
18[39]-C	CACCCAGCTACAAAATAAGAGAATATTTTTATGCTATGGGTGGTCTGGTT	18[39]
18[71]-C	CCCGACTTGCGGGCGACAATAAACAATTTTATGCTATGGGTGGTCTGGTT	18[71]
18[103]-C	ATATAGAAGGCTTTAGATAAGTCCTGTTTTATGCTATGGGTGGTCTGGTT	18[103]
18[135]-C	TTTTTATTTTCATTAGAAACCAATCATTTTATGCTATGGGTGGTCTGGTT	18[135]

Table S10. Staples with extension for hybridization with the Atto488 modified DNA strands and FRET pair modified staple DNA strands on the DNA rafts.

Name	Sequence (5'—3')	5' End
Atto488-ST	Atto488-GGGTTTGGTGTTTTTT	
Atto-1	AAAAAACACCAAACCCTTTTTTTAAATTGTAAATCAGAGCATAAAG	2[39]
Atto-2	AAAAAACACCAAACCCTCCGGTTGATAATCCTTTTGCGGGAGA	2[71]
Atto-3	AAAAAACACCAAACCCTTTTAAGAGAATCGATGCTCATATATTTTA	2[103]]
Atto-4	AAAAAACACCAAACCCTTTTTTTGAGAGATCTACGGGTGAGAAAGGC	2[135]
Atto-5	AAAAAACACCAAACCCTTTTAATTGAGAATCGCCTGTAAATCGTCG	20[39]
Atto-6	AAAAAACACCAAACCCTTTTTGCGTTATACAAACGATAGCTTAGAT	20[71]
Atto-7	AAAAAACACCAAACCCTTTTAAGAATAAACACCCATAGGTCTGAGA	20[103]
Atto-8	AAAAAACACCAAACCCTTTTCCTAAATTTAATGACTATATGTAAAT	20[135]
FRET-Cy3	Cy3- AGCATGTCGCGAGTAACAACC	2[87]
FRET-Cy5	Cy5-TTTCCGGTTGATAATCCTTTTGCGGGAGA	2[71]

Table S11. Parameters for the determination of surface density of 36 ± 9 μm^{-2} (surface coverage of 14.0% ± 3.1%).

	GUV 1	GUV 2	GUV 3	GUV 4	GUV 5	GUV 6
N	3.5	4.0	4.7	5.8	4.5	6.8
σ (μm^{-2})	26	30	35	43	33	51
mean ± standard deviation (σ)	36 ± 9					
φ (%)	10.0	11.6	13.5	16.6	12.7	19.6
mean ± standard deviation (φ)	14.0% ± 3.1%					

Table S12. Parameters for the determination of surface density of 60 ± 10 μm^{-2} (surface coverage of 23.2% ± 3.5%)

	GUV 1	GUV 2	GUV 3	GUV 4	GUV 5	GUV 6
N	7.9	6.9	8.1	10.4	8.4	7.0
σ (μm^{-2})	59	51	60	77	62	52
mean ± standard deviation (σ)	60 ± 10					
φ (%)	22.7	19.6	23.1	29.6	23.9	20.0
mean ± standard deviation (φ)	23.2% ± 3.5%					

Table S13. Parameters for the determination of surface density of 83 ± 10 μm^{-2} (surface coverage of 32% ± 3.5%)

	GUV 1	GUV 2	GUV 3	GUV 4	GUV 5	GUV 6
N	11.2	11.0	12.1	10.2	13.2	9.4
σ (μm^{-2})	83	82	90	76	98	70
mean ± standard deviation (σ)	83 ± 10					
φ (%)	32.0	31.6	34.7	29.3	37.7	27.0
mean ± standard deviation (φ)	32% ± 3.5%					

Table S14. Parameters for the determination of surface density of 100 ± 20 μm^{-2} (surface coverage of 38.5% ± 7%).

	GUV 1	GUV 2	GUV 3	GUV 4	GUV 5	GUV 6
N	18.1	13.4	11.9	14.8	11.0	11.5
σ (μm^{-2})	135	100	88	110	82	85
mean ± standard deviation (σ)	100 ± 20					
φ (%)	52.0	38.5	33.9	42.4	31.6	32.7
mean ± standard deviation (φ)	38.5% ± 7%					

Supplementary Video 1. **a**: GUV deformations induced by the conformation change from the s-DRs to e-DRs after adding unlocking DNA strands. **b**: Control experiment by adding randomly-sequenced DNA strands.

Supplementary Video 2. a: 3D reconstruction of the s-DR-bound GUV based on the z-stack slices. **b**: 3D reconstruction of the e-DR-bound GUV based on the z-stack slices.

Supplementary Video 3. a: GUV shape recovery induced by the conformation change from the e-DRs to R-s-DRs after adding locking DNA strands. **b**: Control experiment by adding randomly-sequenced DNA strands.

Supplementary Video 4. Shape tracing of the GUV slice at the equatorial plane.

Supplementary Video 5. GFP influx in the hybrid DNA raft-OmpF-GUV system.

Supplementary Video 6. a: Sealing of the synthetic channels induced by the conformation change from the e-DRs to R-s-DRs after adding locking strands. **b**: In the absence of locking strands.

Supplementary Data S1 to S51. (Separate file)
Confocal images, the corresponding k_i and circularity ($C = {}^{4\pi A}/_{P^2}$, A is the area and P is the perimeter. For a perfect circle, C=1) of the individual GUVs with different surface densities (~ 36, 60, 83 and 100 μm^{-2}) of the DNA rafts (s-DRs, e-DRs and R-s-DRs) in isoosmotic buffer, after hyperosmotic or hypoosmotic shocks.

Confocal images, the corresponding k_i and circularity of the individual GUVs with different cholesterol patterns (4 Chol, 8 Chol and 16 Chol).

References

1. Song, J. *et al.* Reconfiguration of DNA molecular arrays driven by information relay. *Science* **357**, eaan3377, (2017).
2. Bates, M. A. & Frenkel, D. Phase behavior of two-dimensional hard rod fluids. *J. Chem. Phys* **112**, 10034-10041, (2000).
3. Czogalla, A., Kauert, D. J., Seidel, R., Schwille, P. & Petrov, E. P. DNA Origami Nanoneedles on Freestanding Lipid Membranes as a Tool To Observe Isotropic–Nematic Transition in Two Dimensions. *Nano Lett.* **15**, 649-655, (2015).
4. Müller, P., Schwille, P. & Weidemann, T. PyCorrFit-generic data evaluation for fluorescence correlation spectroscopy. *Bioinformatics* **30**, 2532-2533, (2014).
5. Stachowiak, J. C. *et al.* Membrane bending by protein–protein crowding. *Nat. Cell Biol.* **14**, 944-949, (2012).
6. Seifert, U. Configurations of fluid membranes and vesicles. *Adv. Phys.* **46**, 13-137, (1997).
7. Hess, S. T., Gudheti, M. V., Mlodzianoski, M. & Baumgart, T. Shape analysis of giant vesicles with fluid phase coexistence by laser scanning microscopy to determine curvature, bending elasticity, and line tension. *Methods Mol. Biol.* **400**, 367-387, (2007).
8. Gudheti, M. V., Mlodzianoski, M. & Hess, S. T. Imaging and shape analysis of GUVs as model plasma membranes: effect of trans DOPC on membrane properties. *Biophys. J.* **93**, 2011-2023, (2007).

9. Ahting, U. *et al.* The Tom Core Complex: The General Protein Import Pore of the Outer Membrane of Mitochondria. *J. Cell Biol.* **147**, 959-968, (1999).
10. Bausewein, T. *et al.* Cryo-EM Structure of the TOM Core Complex from Neurospora crassa. *Cell* **170**, 693-700.e697, (2017).
11. Joo, C. & Ha, T. Single-molecule FRET with total internal reflection microscopy. *Cold Spring Harb. protoc.* **2012**, pdb.top072058 (2012).
