# Supplementary material for: Morphology remodelling and membrane channel formation in synthetic cells via reconfigurable DNA nanorafts
Source: Nat Mater. 2025 Jan 13;24(2):278–86. doi: 10.1038/s41563-024-02075-9 (PMC11790494; doi:10.1038/s41563-024-02075-9)
Supplement: Supplementary file 9 — Supplementary Data Fig. 1–51. Confocal images and the corresponding ki of individual GUVs. [file 41563_2024_2075_MOESM9_ESM.pdf]

Supplementary Data for

Morphology remodeling and membrane channel formation in synthetic cells via reconfigurable DNA nanorafts

Sisi Fan[1,2,6], Shuo Wang[3,6], Longjiang Ding[1,2,6], Thomas Speck[4], Hao Yan[5]*, Stephan Nussberger[3]*, Na Liu[1,2]*

[1]2nd Physics Institute, University of Stuttgart, D-70569, Stuttgart, Germany.
[2]Max Planck Institute for Solid State Research, D-70569, Stuttgart, Germany.
[3]Department of Biophysics, Institute of Biomaterials and Biomolecular Systems, University of Stuttgart, D-70569, Stuttgart, Germany.
[4]Institute for Theoretical Physics IV, University of Stuttgart, D-70569, Stuttgart, Germany.
[5]Biodesign Center for Molecular Design and Biomimetics, Arizona State University, Tempe, AZ 85287, USA.
[6]These authors contributed equally to this work.
* Corresponding authors.
Email: hao.yan@asu.edu; stephan.nussberger@bio.uni-stuttgart.de; na.liu@pi2.uni-stuttgart.de

The file includes: Data S1 to S51

Data S1-S3: Confocal images, the corresponding k_i and circularity of individual GUVs by the 12d cholesterol anchored DNA rafts (s-DRs (Data S1), e-DRs (Data S2) and R-s-DRs (Data S3)) with a surface density of ~ 36 μm^{-2} after hyperosmotic shocks.

Data S4-S6: Confocal images, the corresponding k_i and circularity of individual GUVs by the 12d cholesterol anchored DNA rafts (s-DRs (Data S4), e-DRs (Data S5) and R-s-DRs (Data S6)) with a surface density of ~ 36 μm^{-2} in isoosmotic buffer.

Data S7-S9: Confocal images, the corresponding k_i and circularity of individual GUVs by the 12d cholesterol anchored DNA rafts (s-DRs (Data S7), e-DRs (Data S8) and R-s-DRs (Data S9)) with a surface density of ~ 36 μm^{-2} after hypoosmotic shocks.

Data S10-S12: Confocal images, the corresponding k_i and circularity of individual GUVs by the 12d cholesterol anchored DNA rafts (s-DRs (Data S10), e-DRs (Data S11) and R-s-DRs (Data S12)) with a surface density of ~ 60 μm^{-2} after hyperosmotic shocks.

Data S13-S15: Confocal images, the corresponding k_i and circularity of individual GUVs by the 12d cholesterol anchored DNA rafts (s-DRs (Data S13), e-DRs (Data S14) and R-s-DRs (Data S15)) with a surface density of ~ 60 μm^{-2} after hypoosmotic shocks.

Data S16-S18: Confocal images, the corresponding k_i and circularity of individual GUVs by the 12d cholesterol anchored DNA rafts (s-DRs (Data S16), e-DRs (Data S17) and R-s-DRs (Data S18)) with a surface density of ~ 83 μm^{-2} after hyperosmotic shocks.

Data S19-S21: Confocal images, the corresponding k_i and circularity of individual GUVs by the 12d cholesterol anchored DNA rafts (s-DRs (Data S19), e-DRs (Data S20) and R-s-DRs (Data S21)) with a surface density of ~ 83 μm^{-2} in isoosmotic buffer.

Data S22-S24: Confocal images, the corresponding k_i and circularity of individual GUVs by the 12d cholesterol anchored DNA rafts (s-DRs (Data S22), e-DRs (Data S23) and R-s-DRs (Data S24)) with a surface density of ~ 83 μm^{-2} after hypoosmotic shocks.

Data S25-S27: Confocal images, the corresponding k_i and circularity of individual GUVs by the 12d cholesterol anchored DNA rafts (s-DRs (Data S25), e-DRs (Data S26) and R-s-DRs (Data S27)) with a surface density of ~ 100 μm^{-2} after hyperosmotic shocks.

Data S28-S30: Confocal images, the corresponding k_i and circularity of individual GUVs by the 12d cholesterol anchored DNA rafts (s-DRs (Data S28), e-DRs (Data S29) and R-s-DRs (Data S30)) with a surface density of ~ 100 μm^{-2} in isoosmotic buffer.

Data S31-S33: Confocal images, the corresponding k_i and circularity of individual GUVs by the 12d cholesterol anchored DNA rafts (s-DRs (Data S31), e-DRs (Data S32) and R-s-DRs (Data S33)) with a surface density of ~ 100 μm^{-2} after hypoosmotic shocks.

Data S34-S36: Confocal images, the corresponding k_i and circularity of individual GUVs by the 12a cholesterol anchored DNA rafts (s-DRs (Data S34), e-DRs (Data S35) and R-s-DRs (Data S36)) in isoosmotic buffer.

Data S37-S39: Confocal images, and the corresponding k_i and circularity of individual GUVs by the 12b cholesterol anchored DNA rafts (s-DRs (Data S37), e-DRs (Data S38) and R-s-DRs (Data S39)) in isoosmotic buffer.

Data S40-S42: Confocal images and the corresponding k_i and circularity of individual GUVs by the 12c cholesterol anchored DNA rafts (s-DRs (Data S40), e-DRs (Data S41) and R-s-DRs (Data S42)) in isoosmotic buffer.

Data S43-S45: Confocal images, the corresponding k_i and circularity of individual GUVs by the 4 cholesterol anchored DNA rafts (s-DRs (Data S43), e-DRs (Data S44) and R-s-DRs (Data S45)) in isoosmotic buffer.

Data S46-S48: Confocal images, the corresponding k_i and circularity of individual GUVs by the 8 cholesterol anchored DNA rafts (s-DRs (Data S46), e-DRs (Data S47) and R-s-DRs (Data S48)) in isoosmotic buffer.

Data S49-S51: Confocal images, the corresponding k_i and circularity of individual GUVs by the 16 cholesterol anchored DNA rafts (s-DRs (Data S49), e-DRs (Data S50) and R-s-DRs (Data S51)) in isoosmotic buffer.

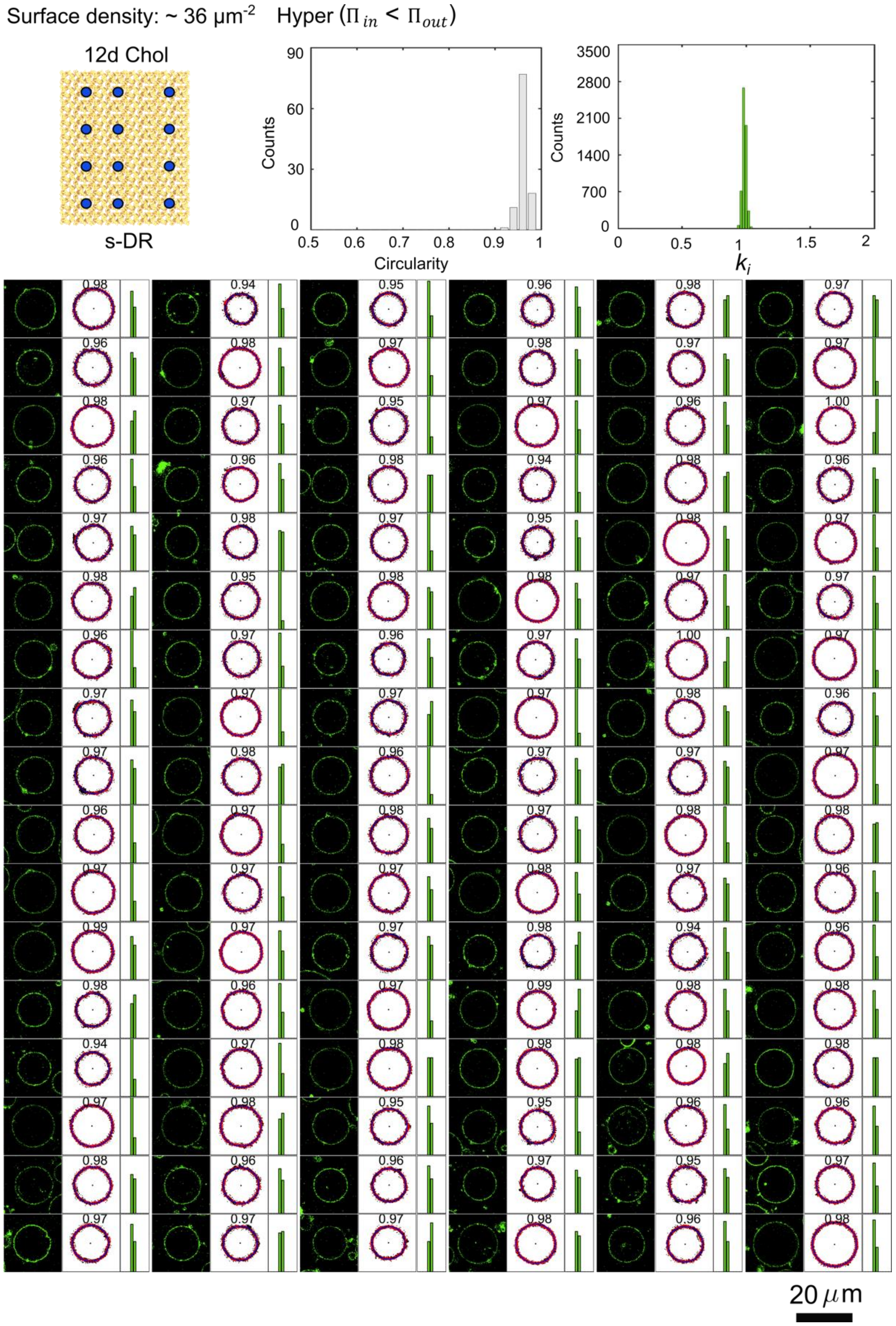

Data S1. 12d Chol s-DR-bound GUVs after hyperosmotic shocks. The deformation efficiency is ~4.4%.

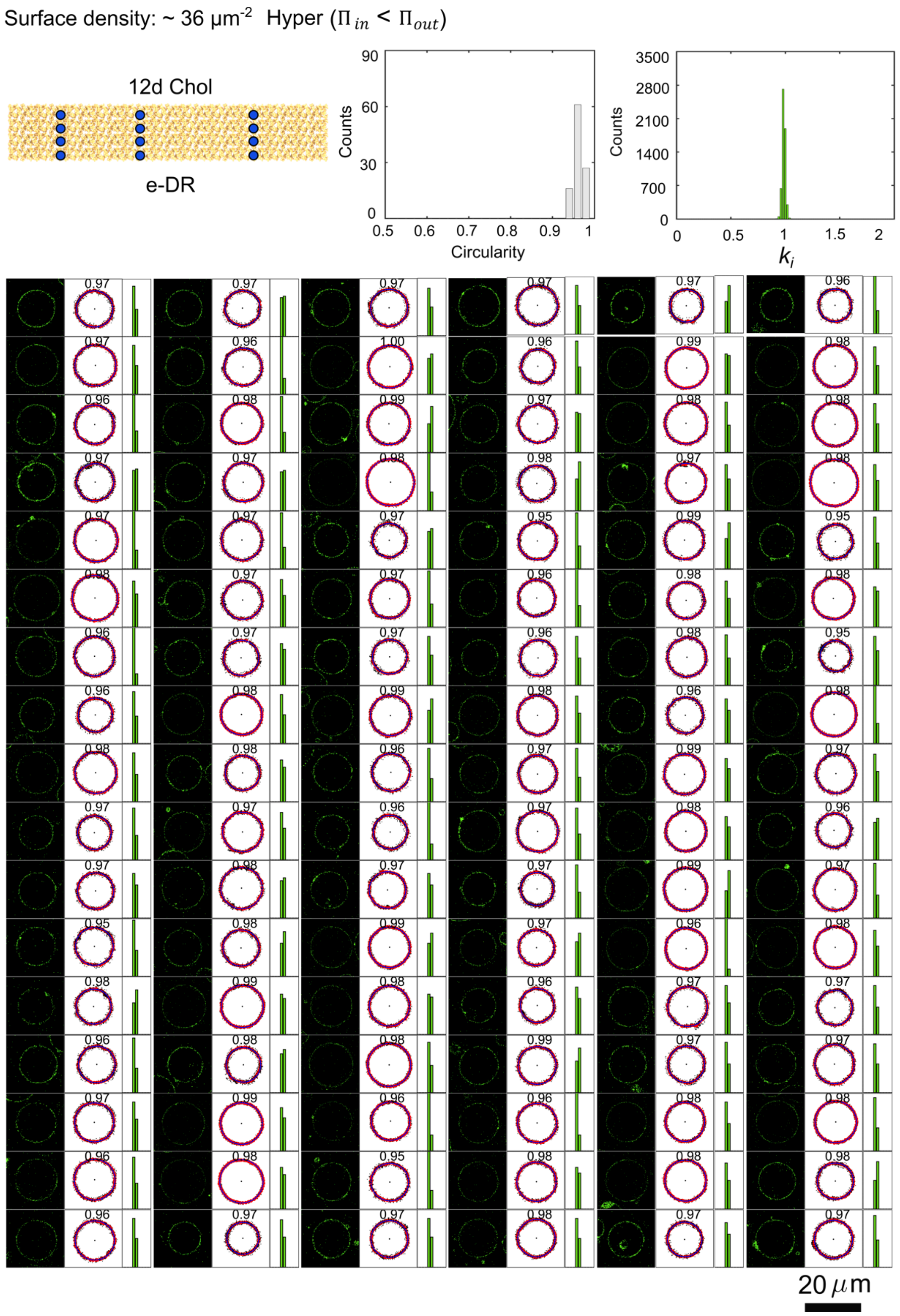

Data S2. 12d Chol e-DR-bound GUVs after hyperosmotic shocks. The deformation efficiency is ~1.9%.

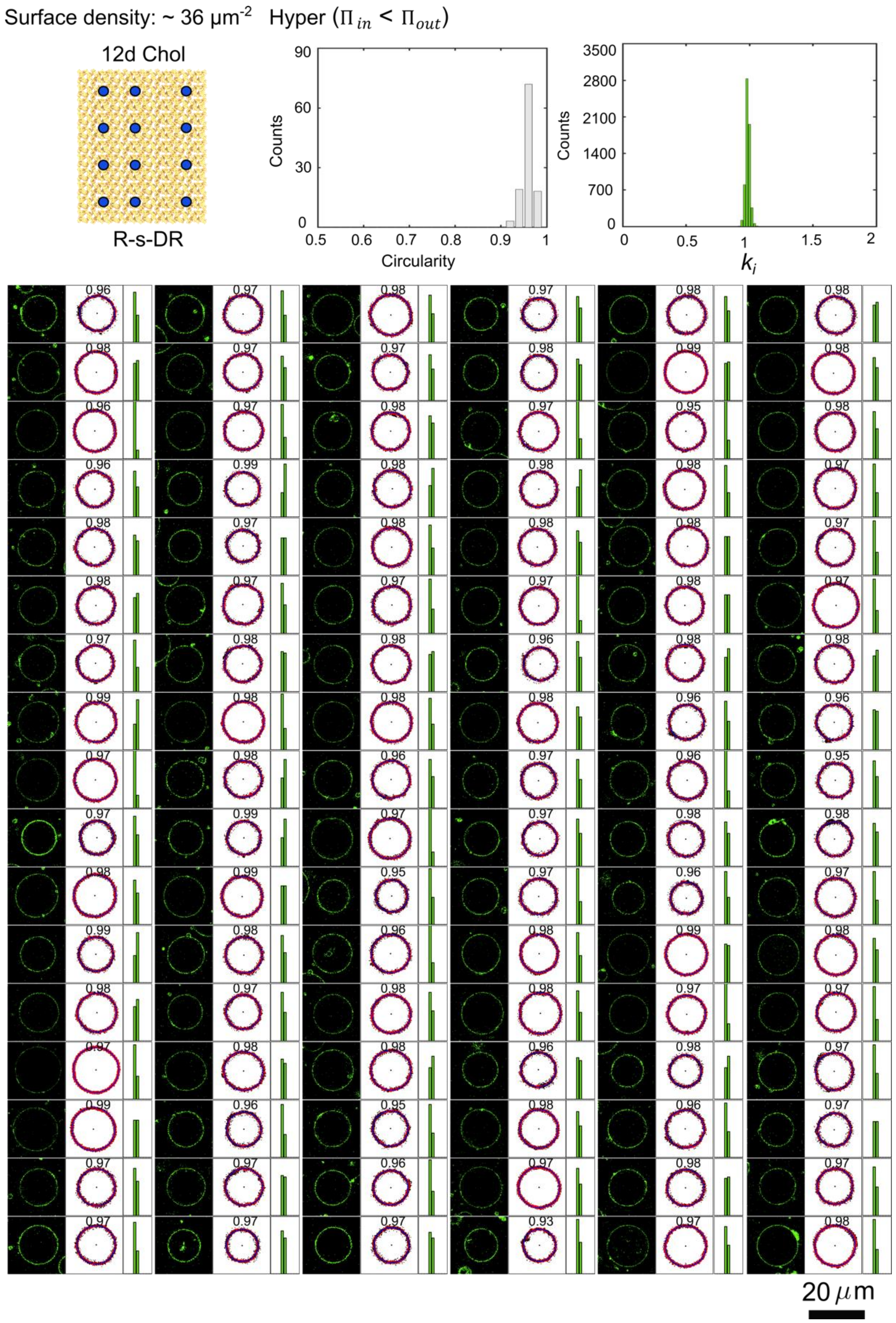

Data S3. 12d Chol R-s-DR-bound GUVs after hyperosmotic shocks. The deformation efficiency is ~0%.

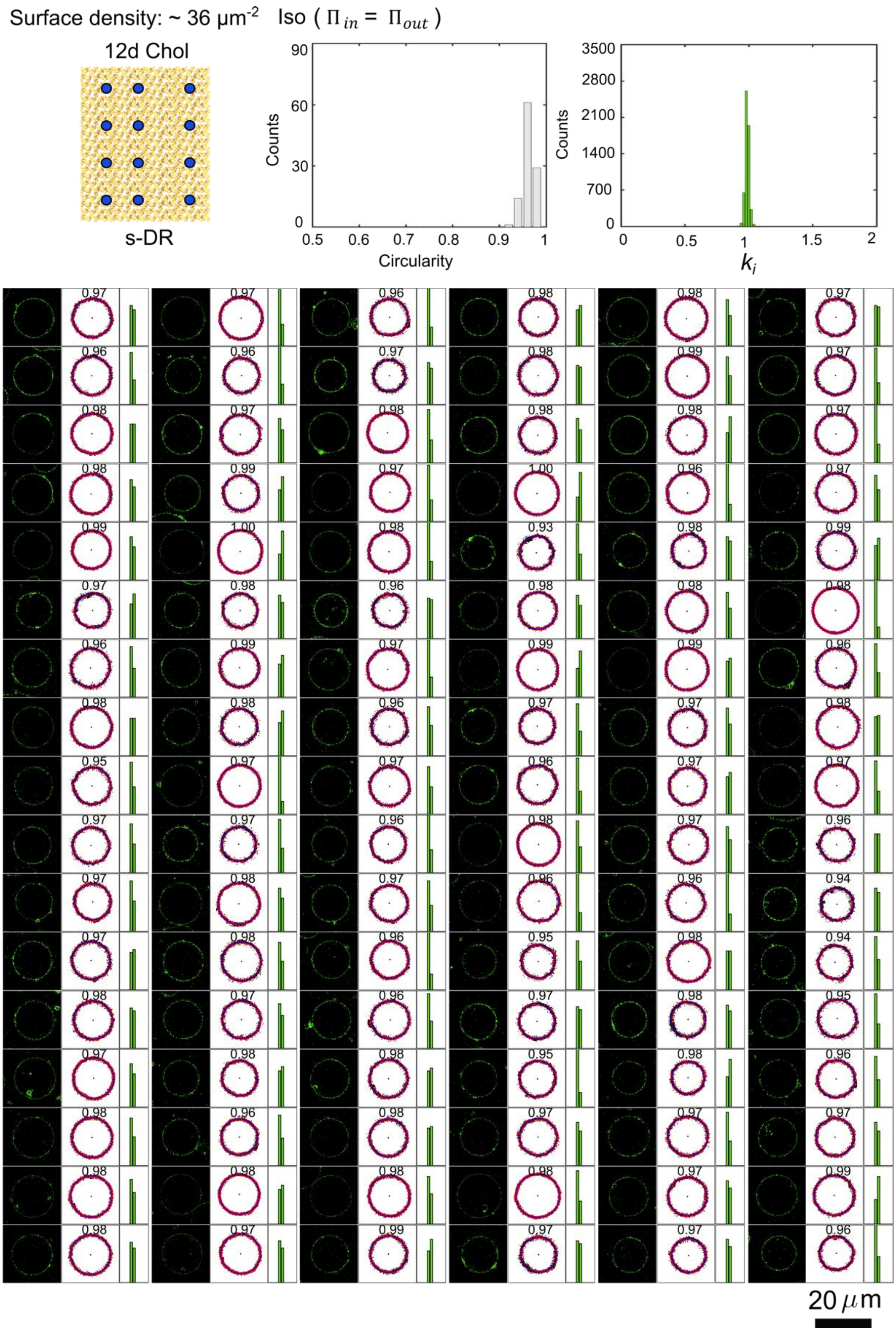

Data S4. 12d Chol s-DR-bound GUVs in isoosmotic buffer. The deformation efficiency is ~2.8%.

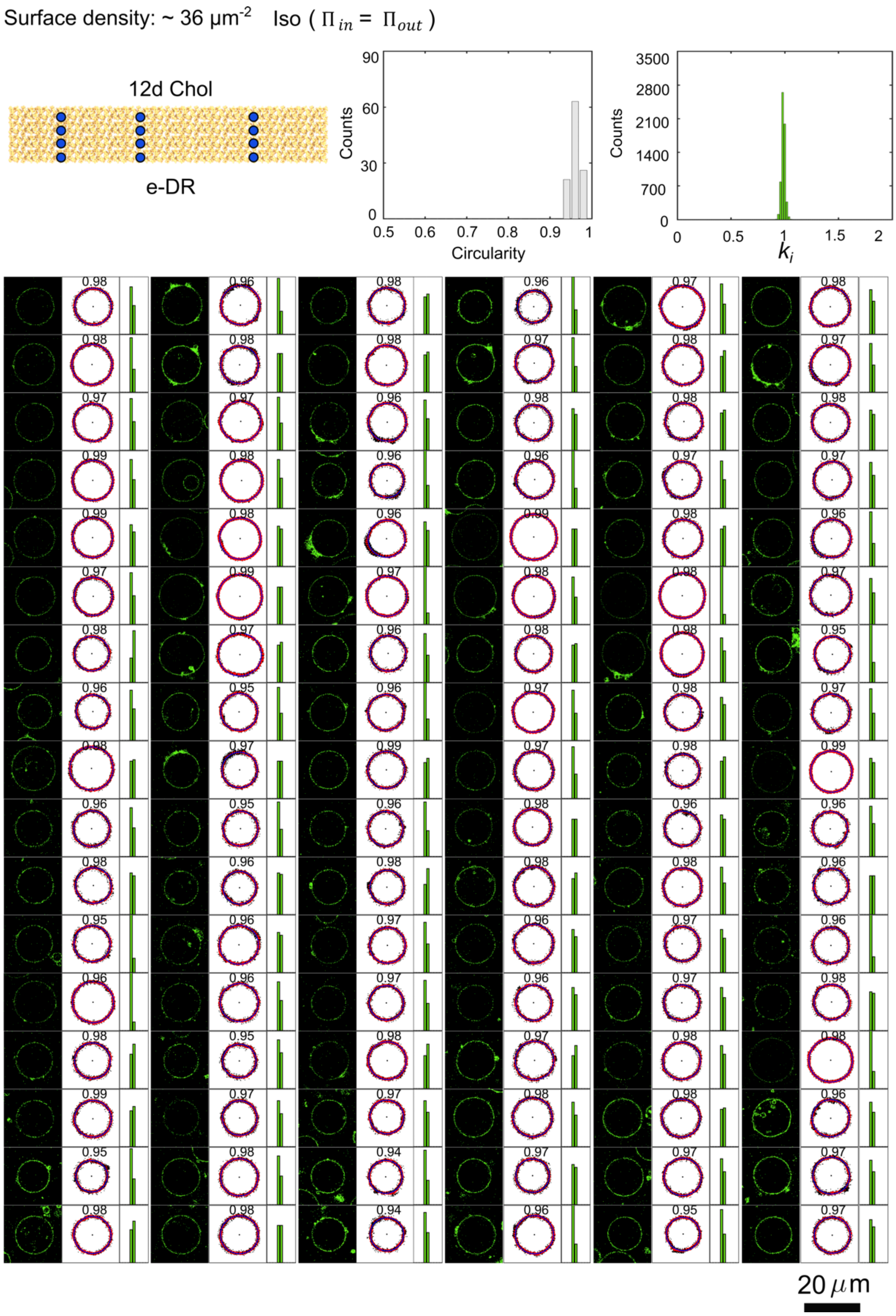

Data S5. 12d Chol e-DR-bound GUVs in isoosmotic buffer. The deformation efficiency is ~2.7%.

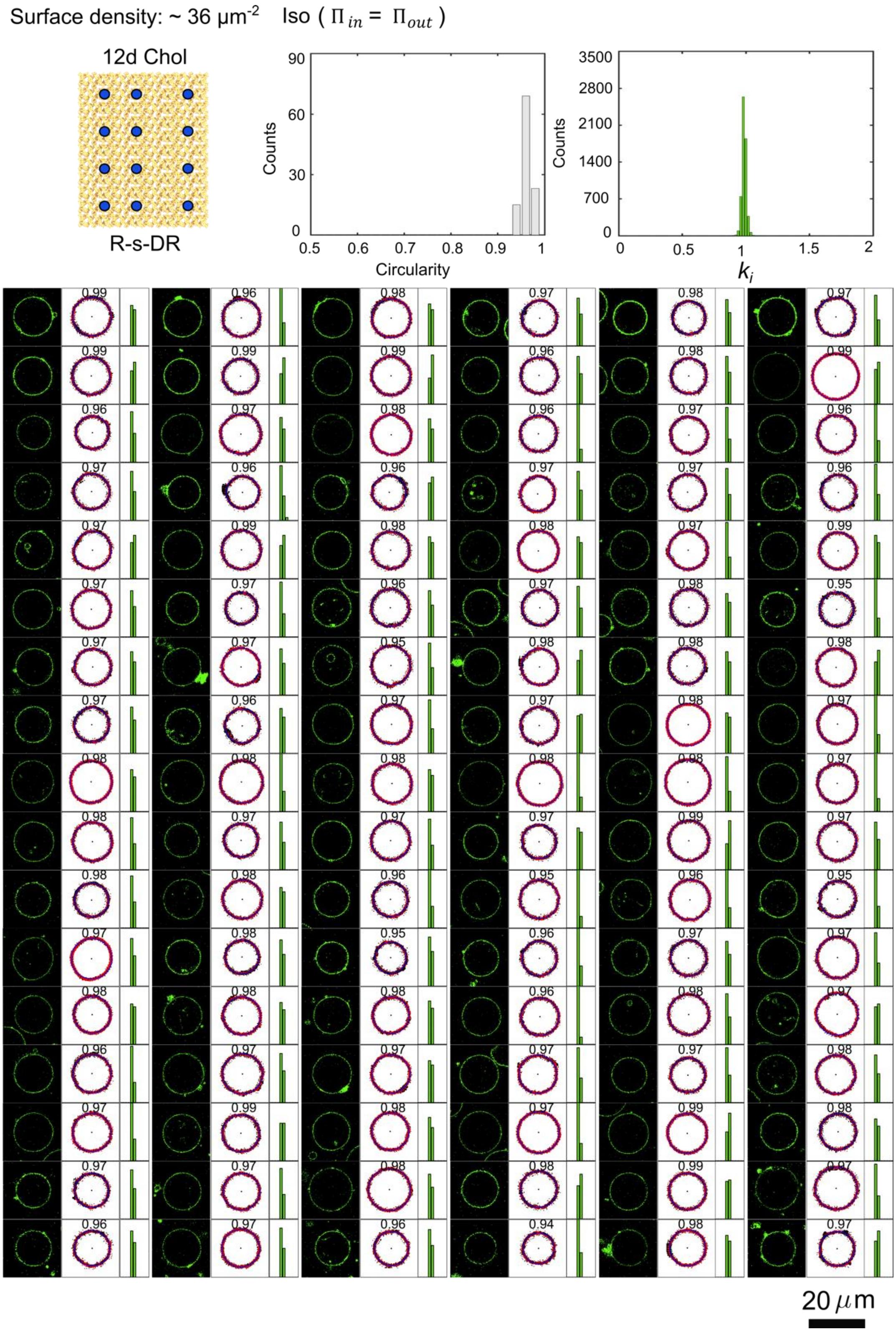

Data S6. 12d Chol R-s-DR-bound GUVs in isoosmotic buffer. The deformation efficiency is ~3.7%.

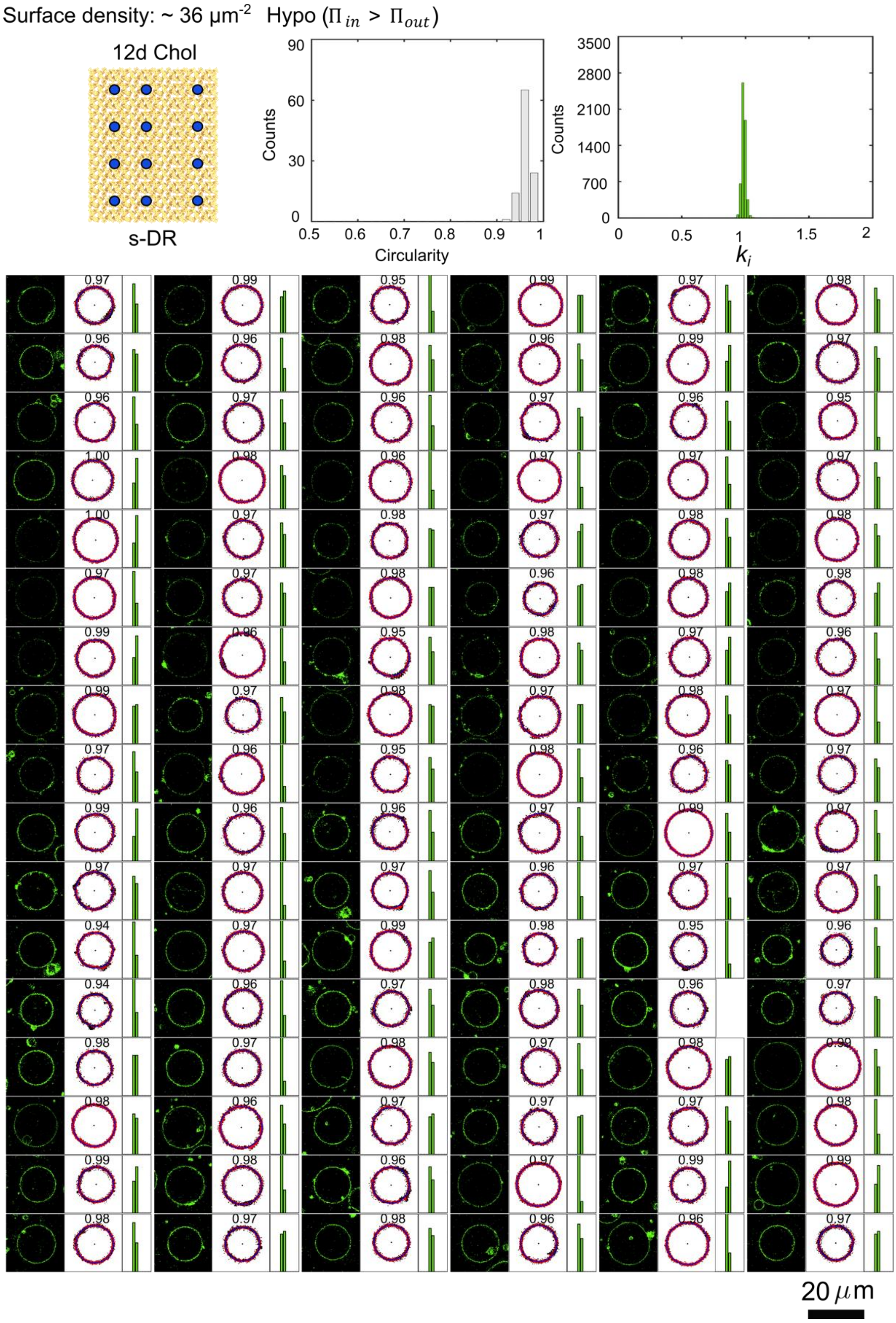

Data S7. 12d Chol s-DR-bound GUVs after hypoosmotic shocks. The deformation efficiency is ~1.3%.

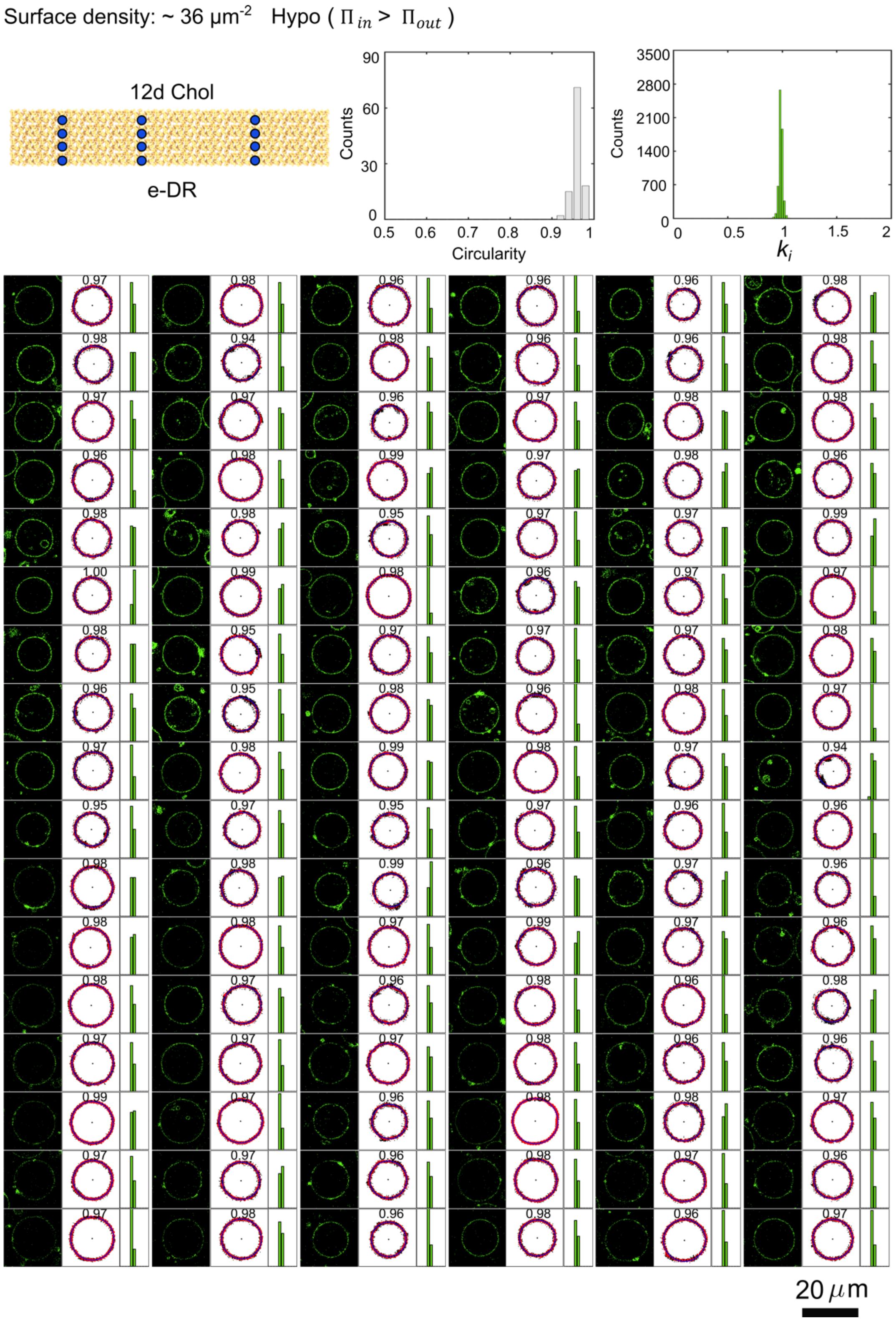

Data S8. 12d Chol e-DR-bound GUVs after hypoosmotic shocks. The deformation efficiency is ~3.8%.

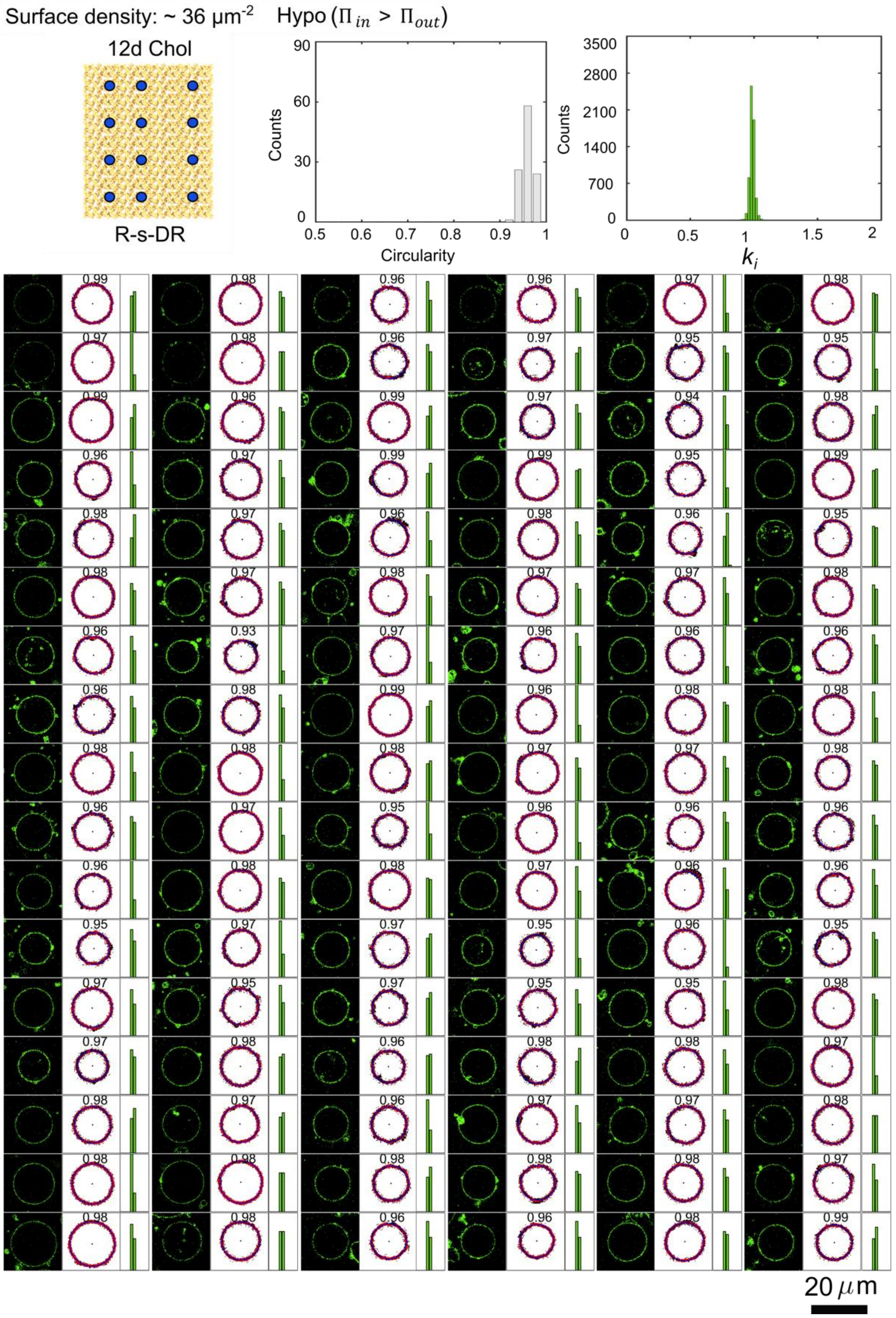

Data S9. 12d Chol R-s-DR-bound GUVs after hypoosmotic shocks. The deformation efficiency is ~3.7%.

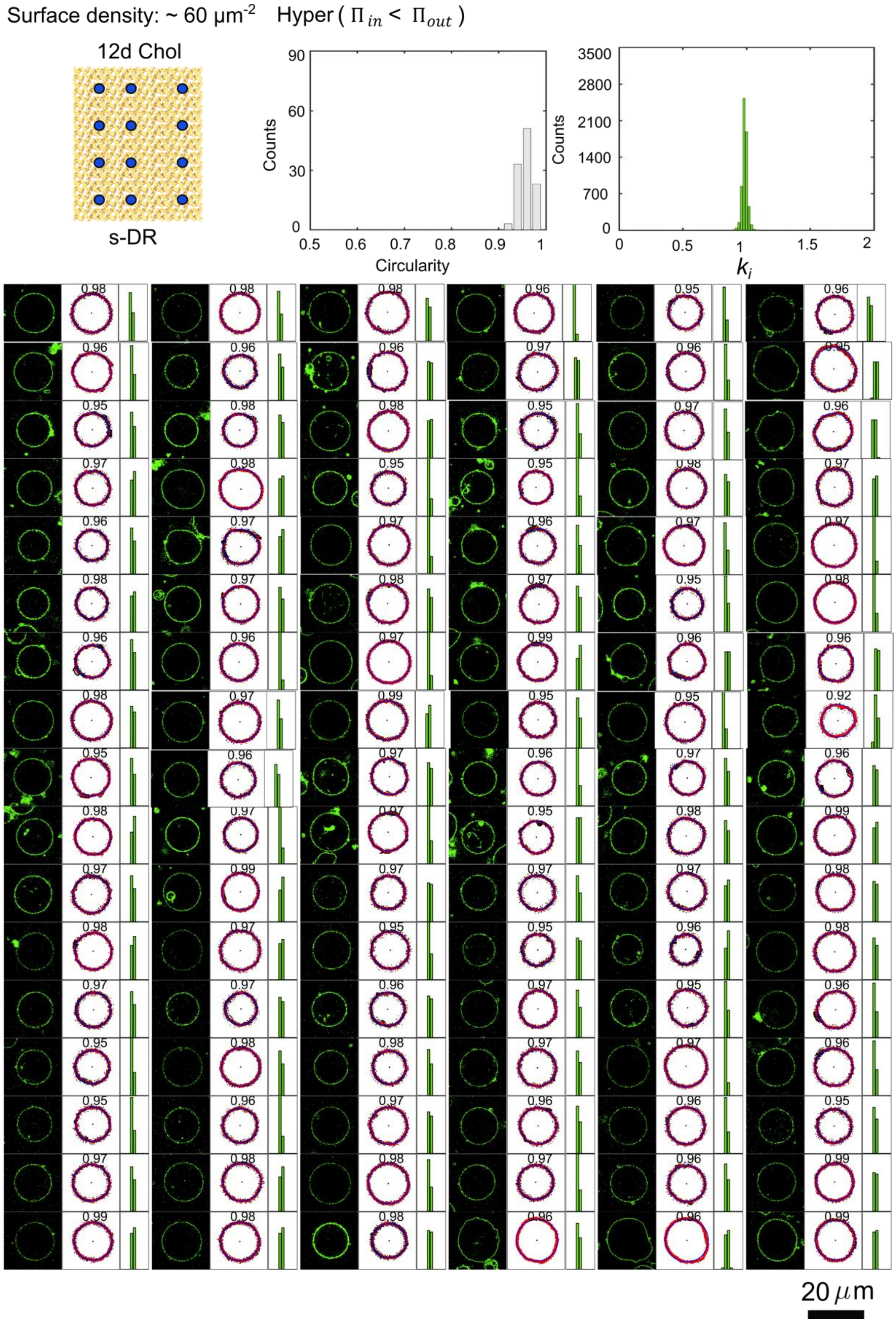

Data S10. 12d Chol s-DR-bound GUVs after hyperosmotic shocks. The deformation efficiency is ~10%.

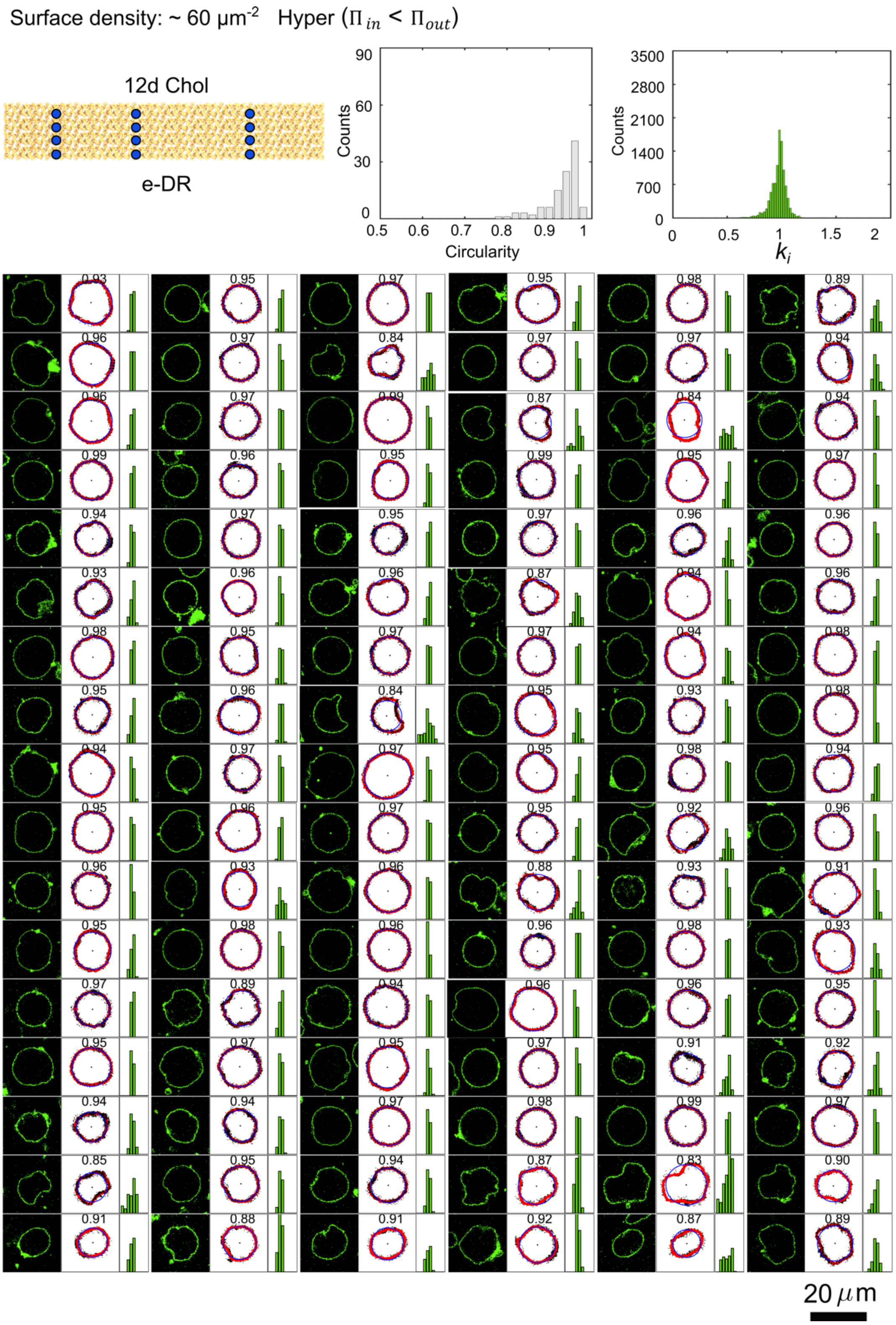

Data S11. 12d Chol e-DR-bound GUVs after hyperosmotic shocks. The deformation efficiency is ~80%.

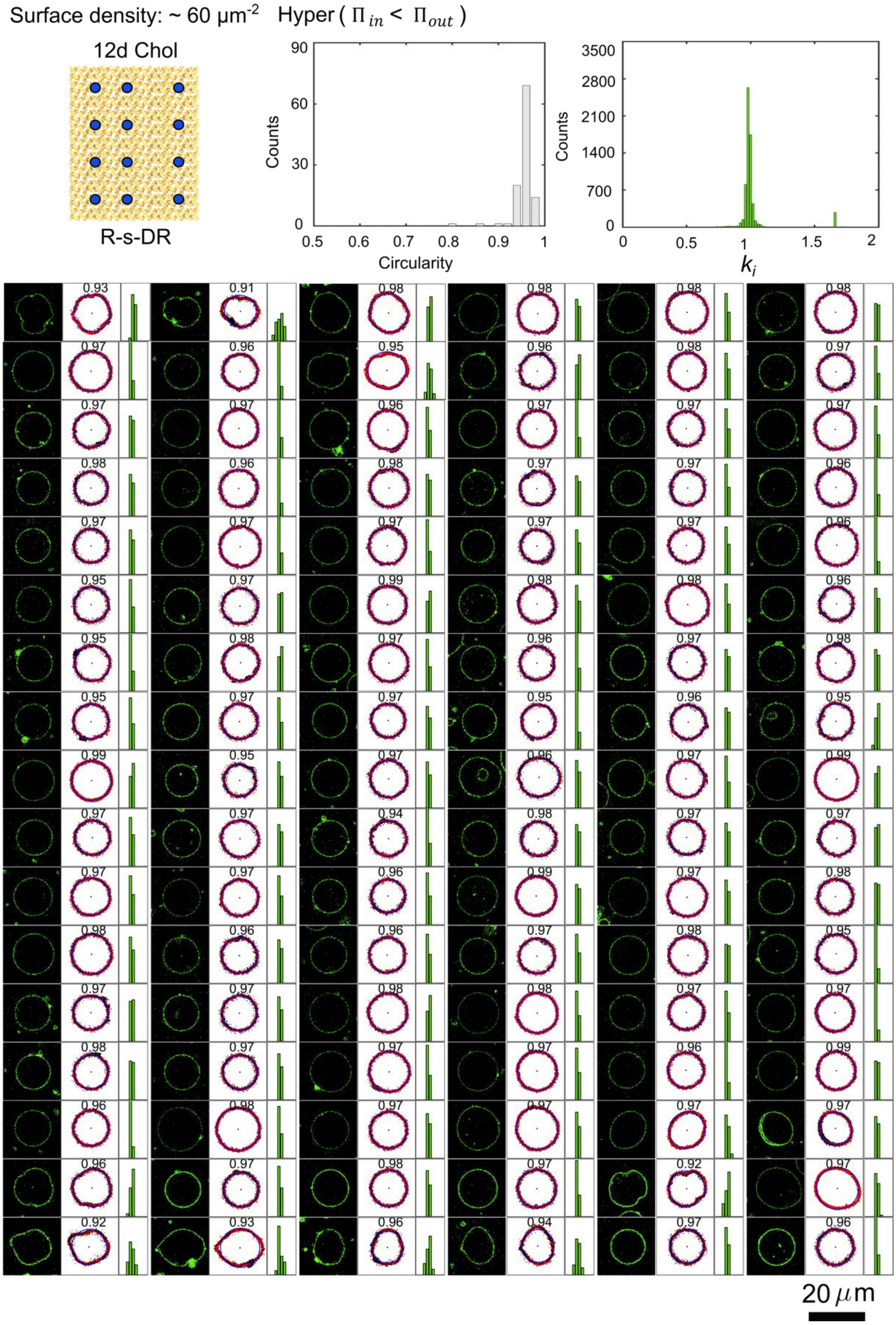

Data S12. 12d Chol R-s-DR-bound GUVs after hyperosmotic shocks. The deformation efficiency is ~11.1%.

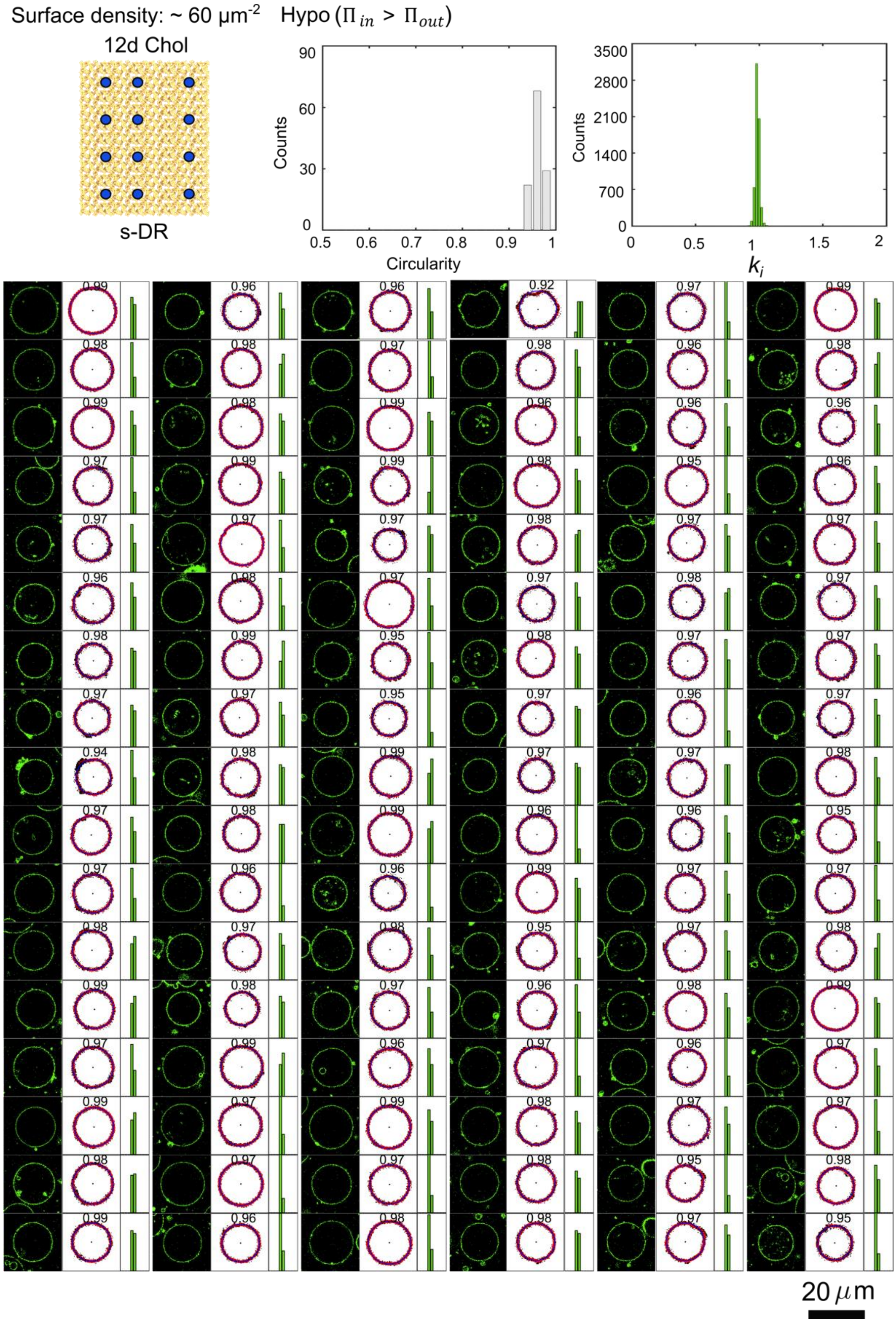

Data S13. 12d Chol s-DR-bound GUVs after hypoosmotic shocks. The deformation efficiency is ~1.7%.

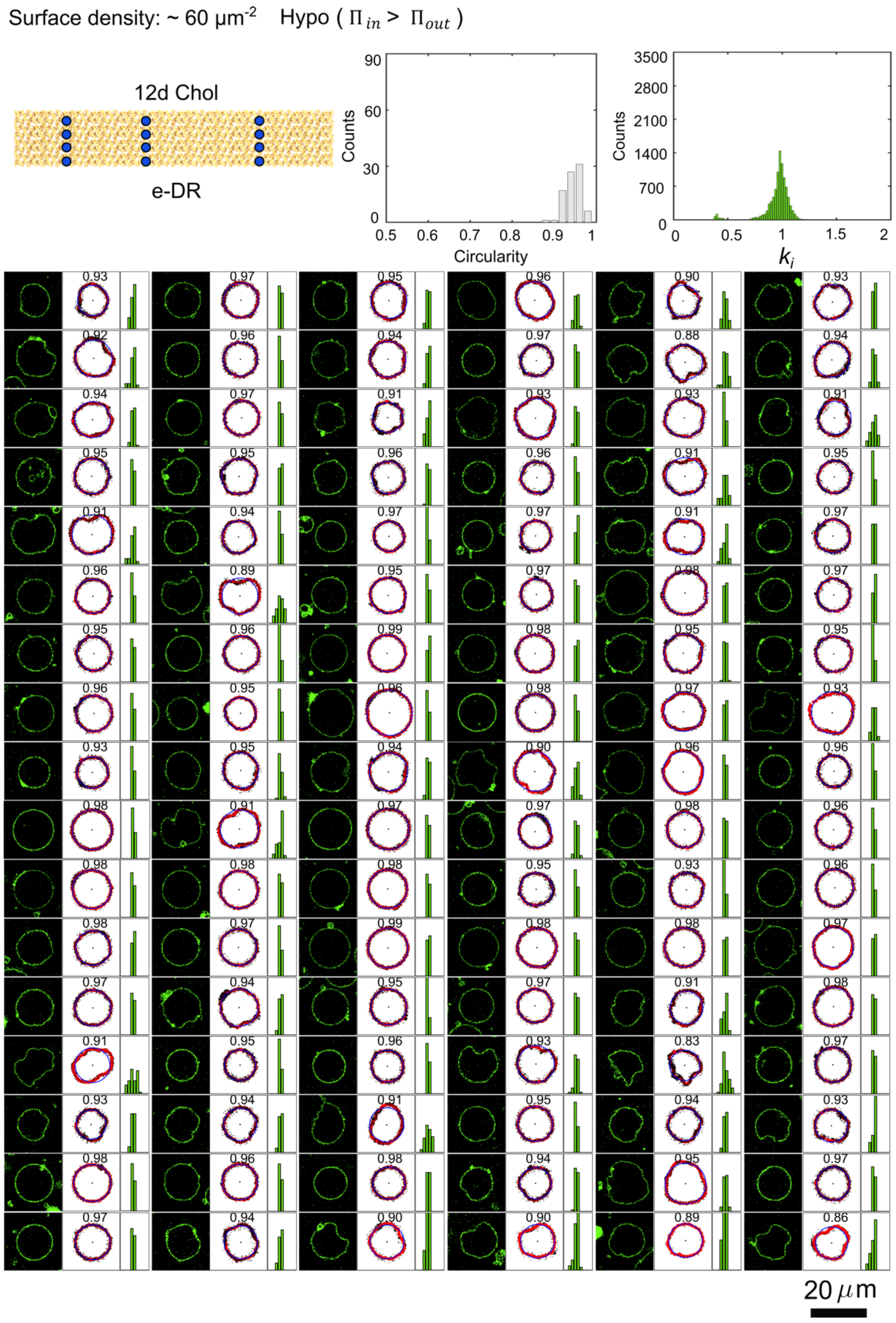

Data S14. 12d Chol e-DR-bound GUVs after hypoosmotic shocks. The deformation efficiency is ~41.1%.

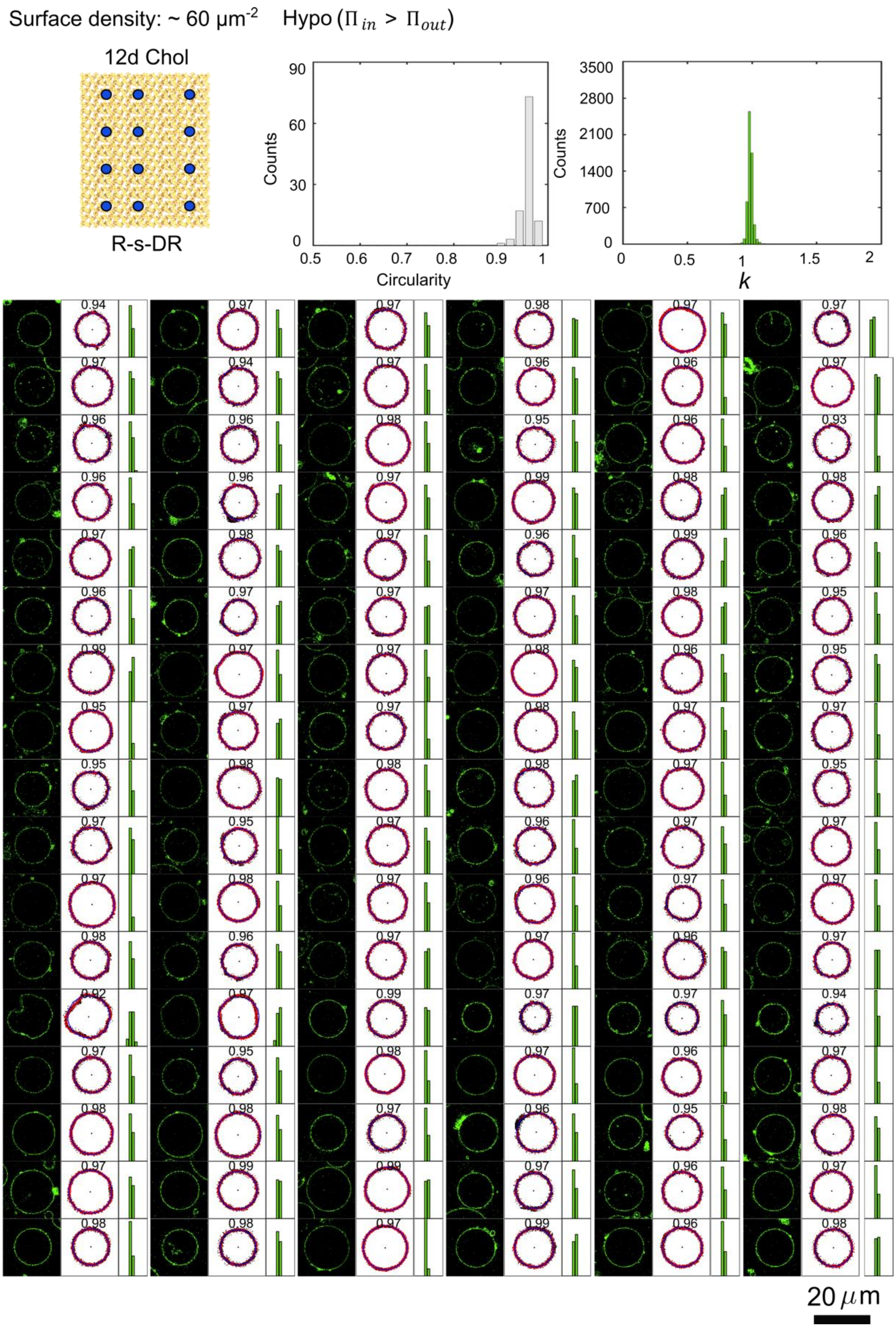

Data S15. 12d Chol R-s-DR-bound GUVs after hypoosmotic shocks. The deformation efficiency is ~7.5%.

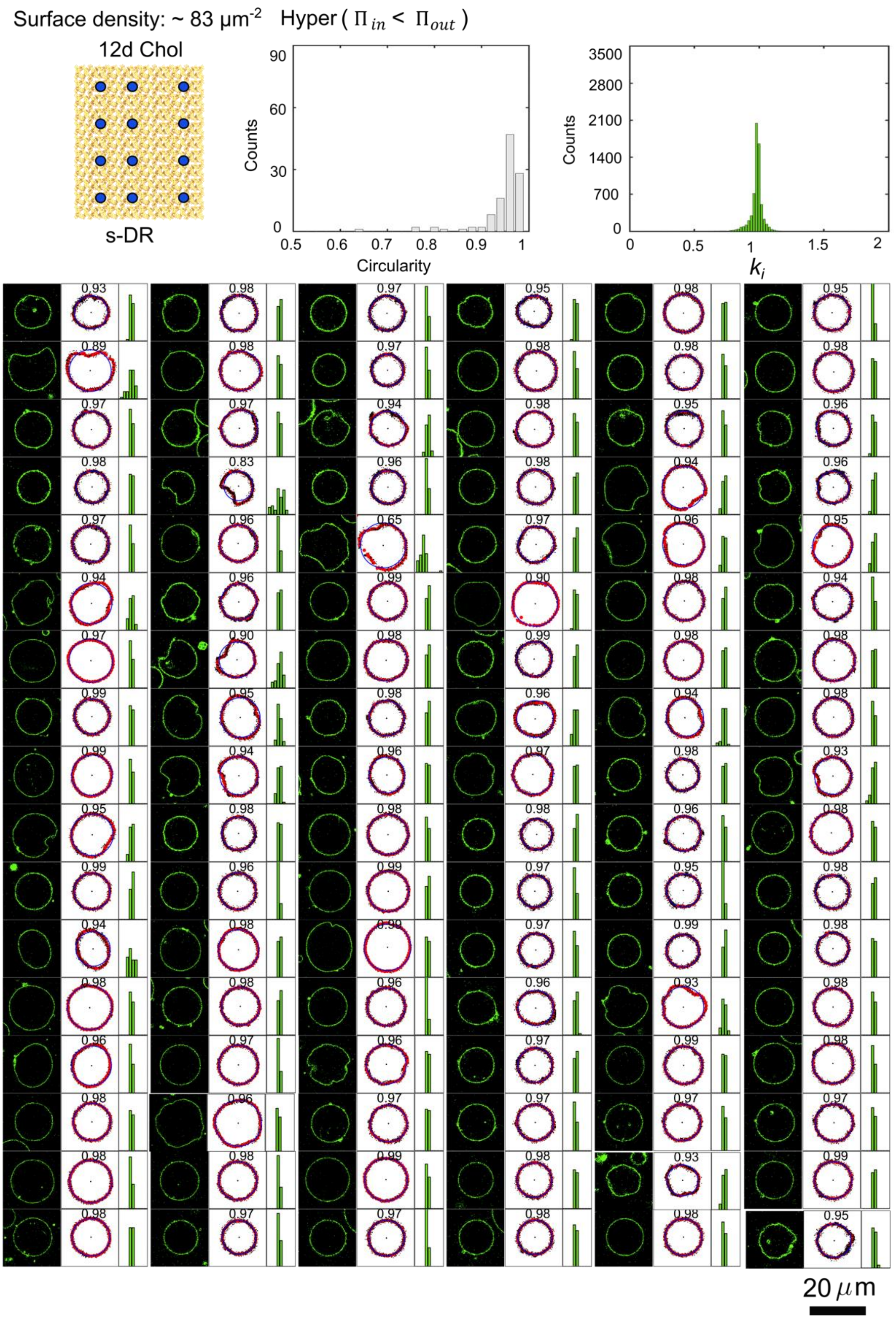

Data S16. 12d Chol s-DR-bound GUVs after hyperosmotic shocks. The deformation efficiency of the GUVs by 12d Chol s-DR is ~30.7%.

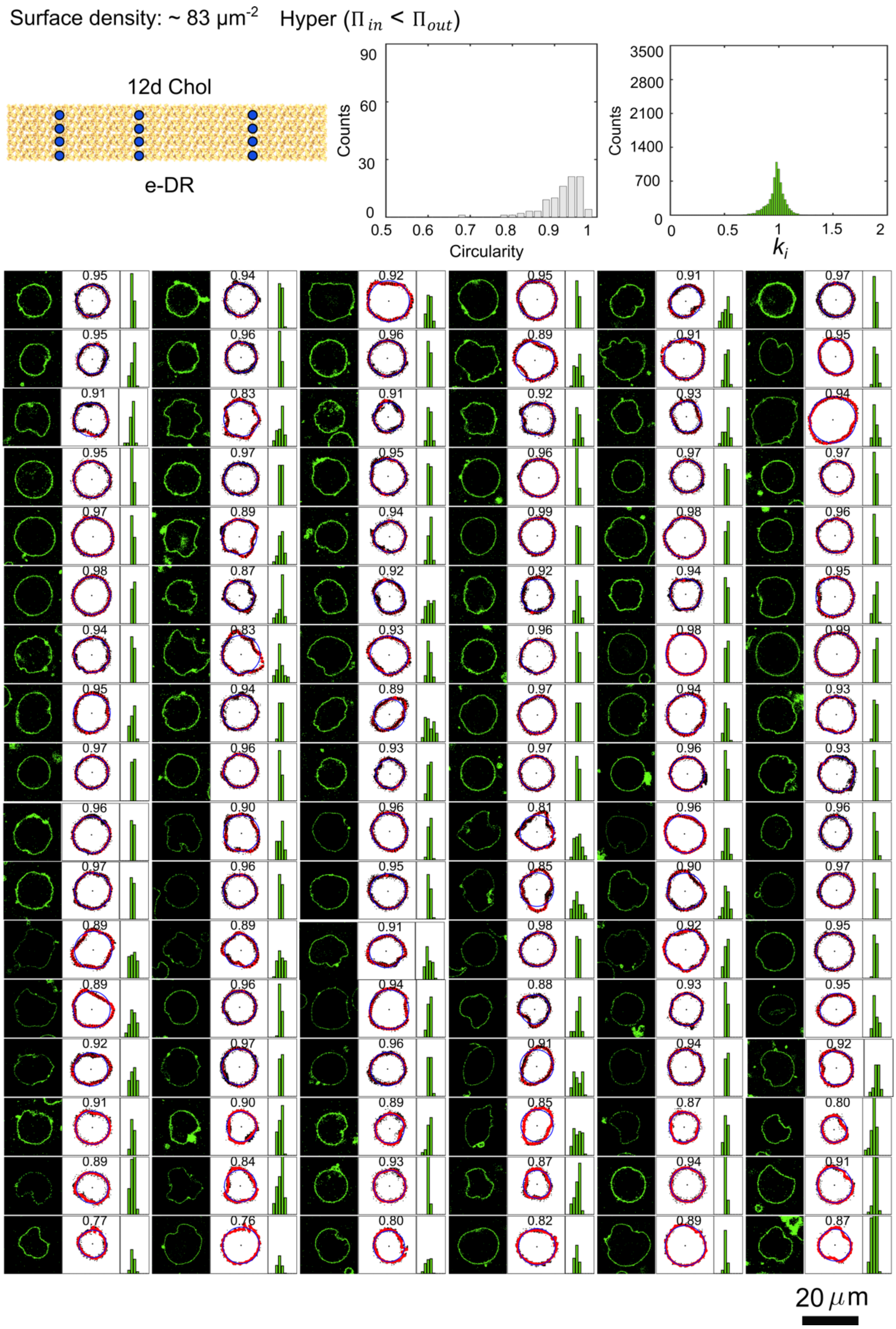

Data S17. 12d Chol e-DR-bound GUVs after hyperosmotic shocks. The deformation efficiency is ~75.5%.

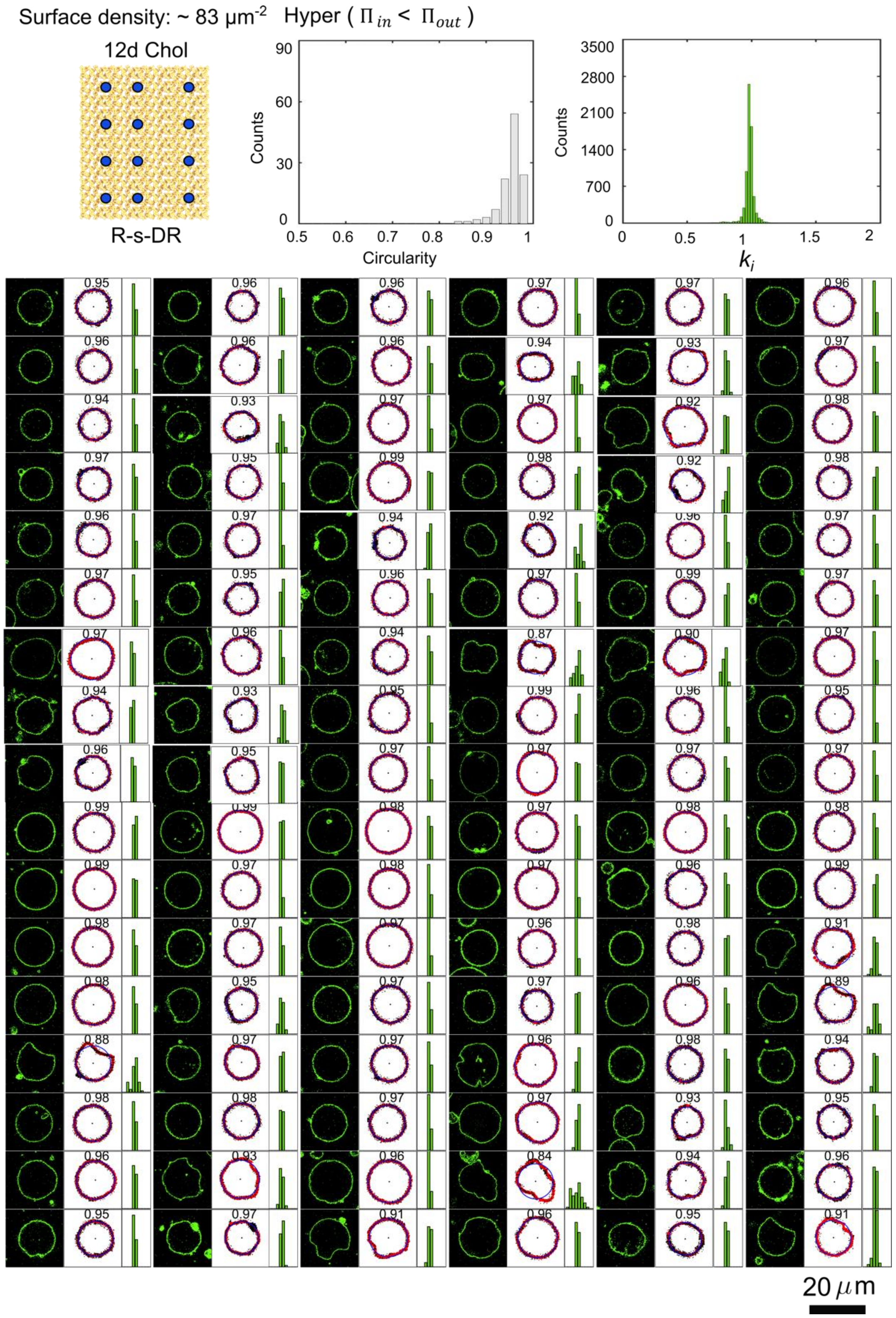

Data S18. 12d Chol R-s-DR-bound GUVs after hyperosmotic shocks. The deformation efficiency is ~39.1%.

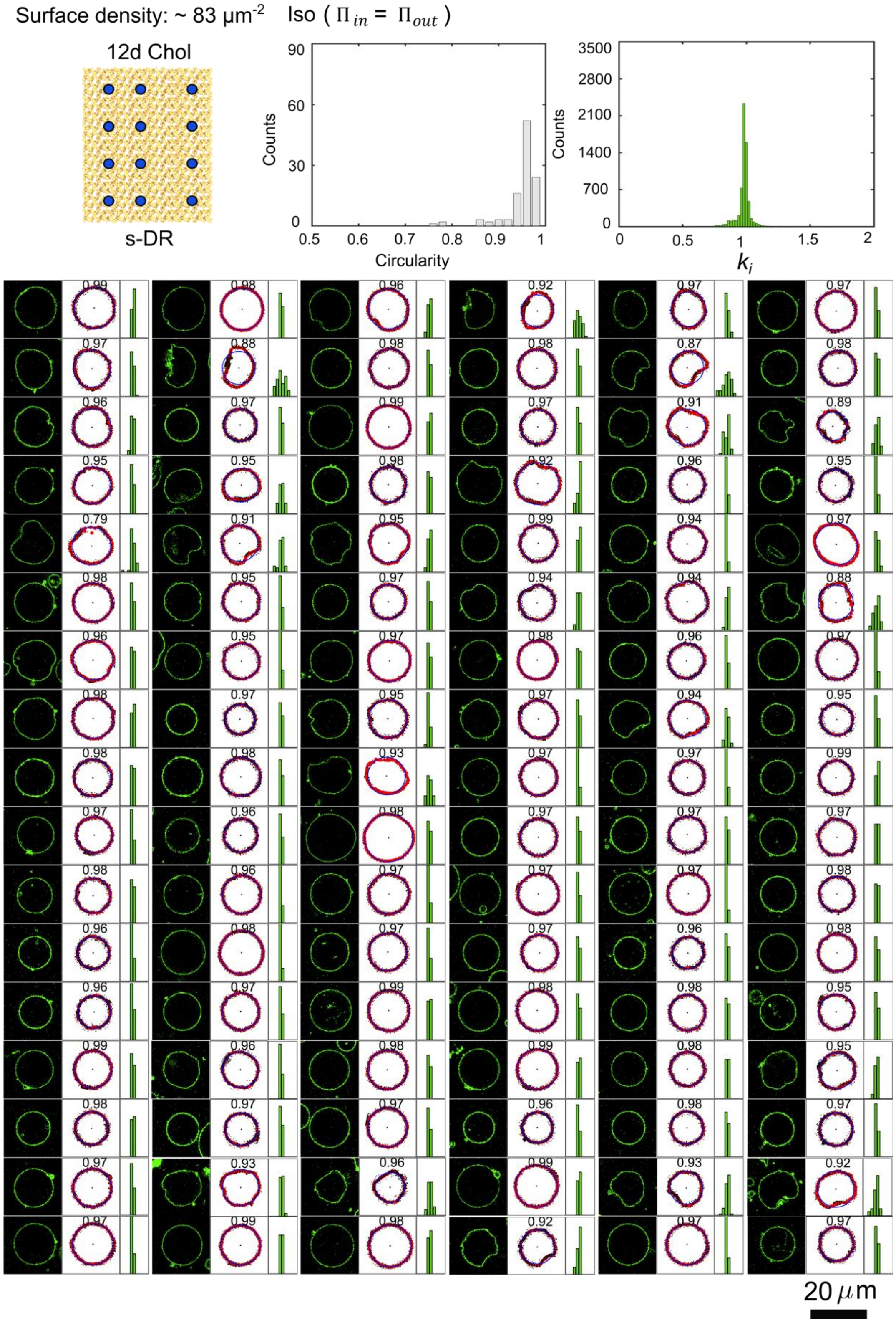

Data S19. 12d Chol s-DR-bound GUVs in isoosmotic buffer. The deformation efficiency is ~29.1%.

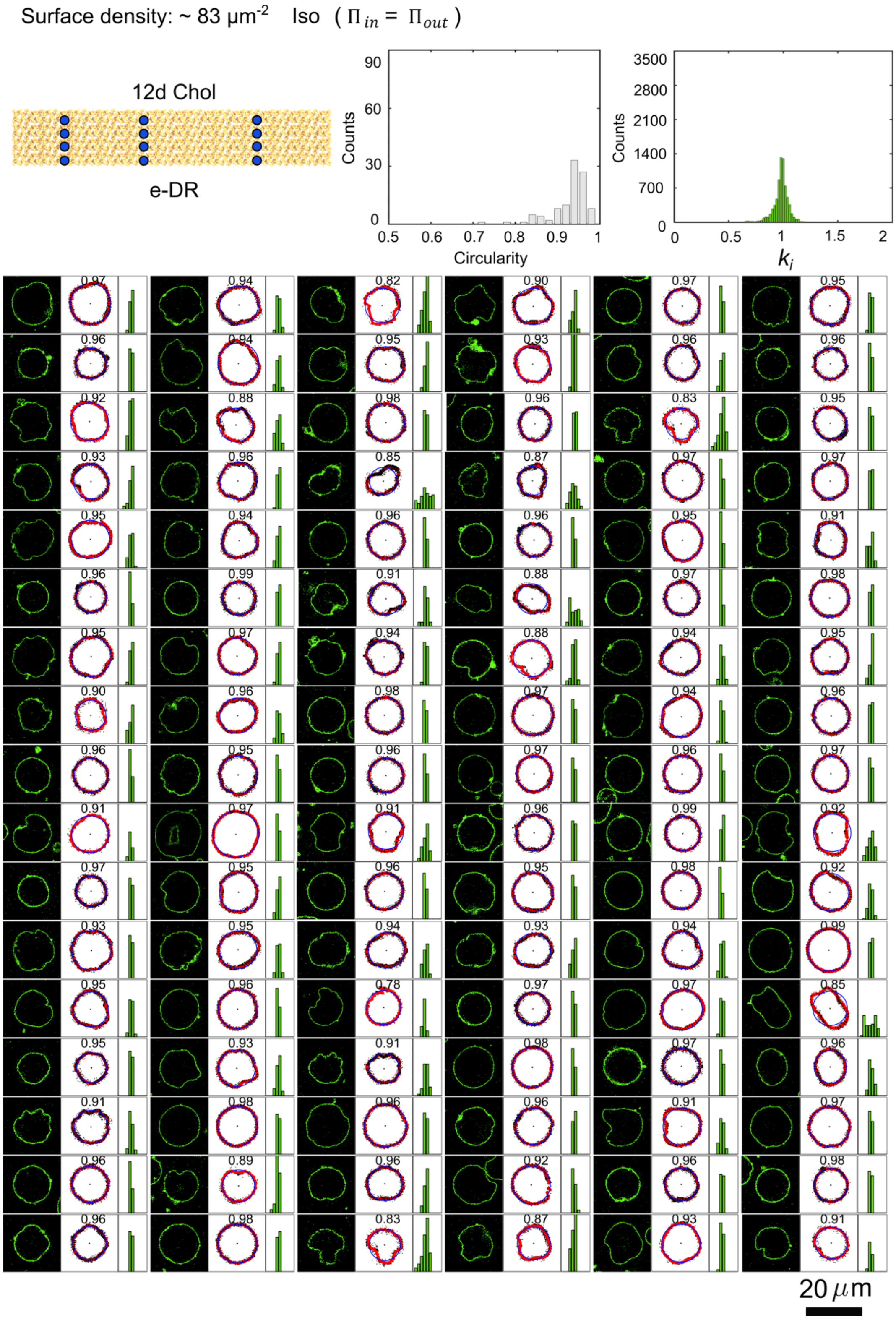

Data S20. 12d Chol e-DR-bound GUVs in isoosmotic buffer. The deformation efficiency is ~69.8%.

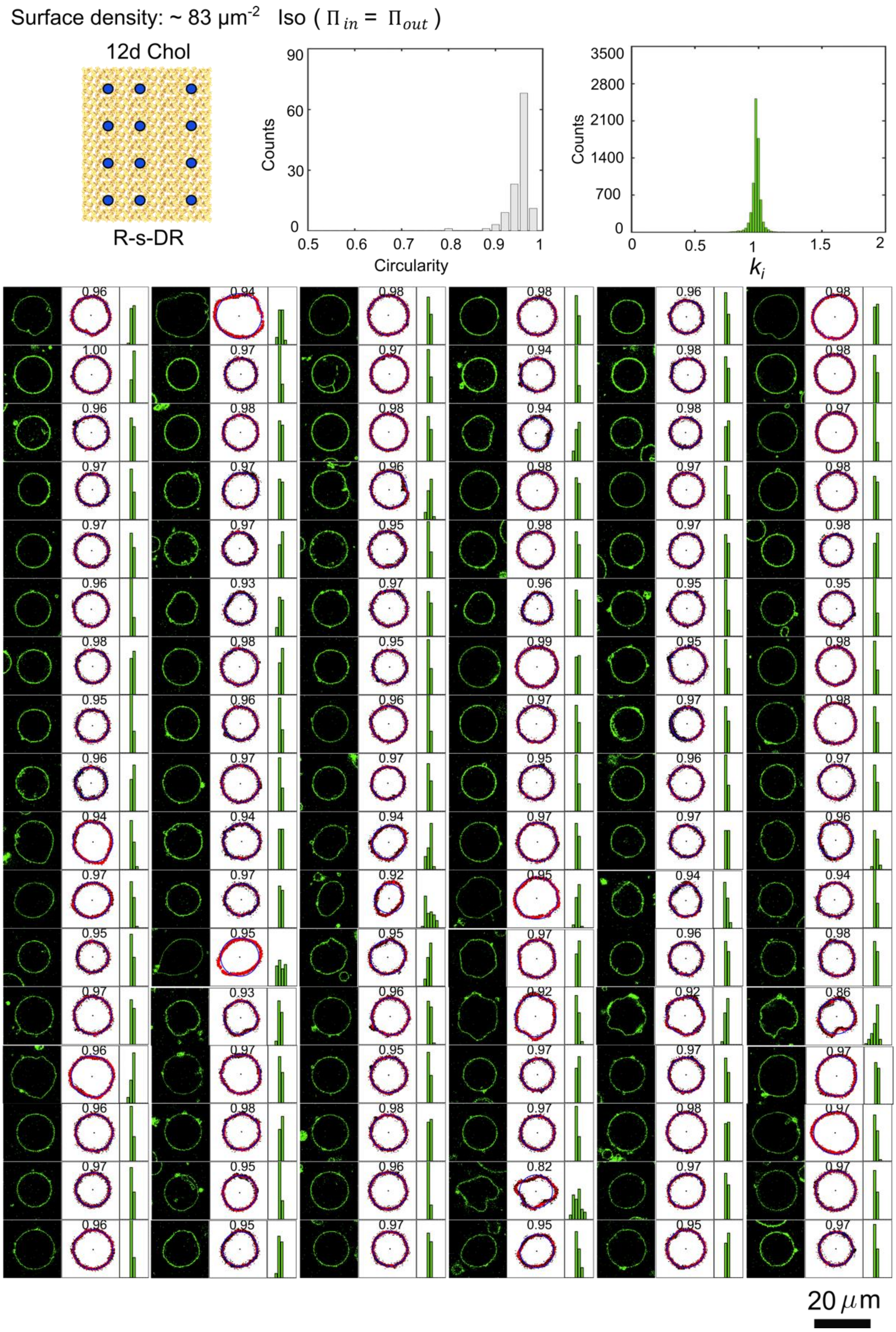

Data S21. 12d Chol R-s-DR-bound GUVs in isoosmotic buffer. The deformation efficiency is ~26.4%.

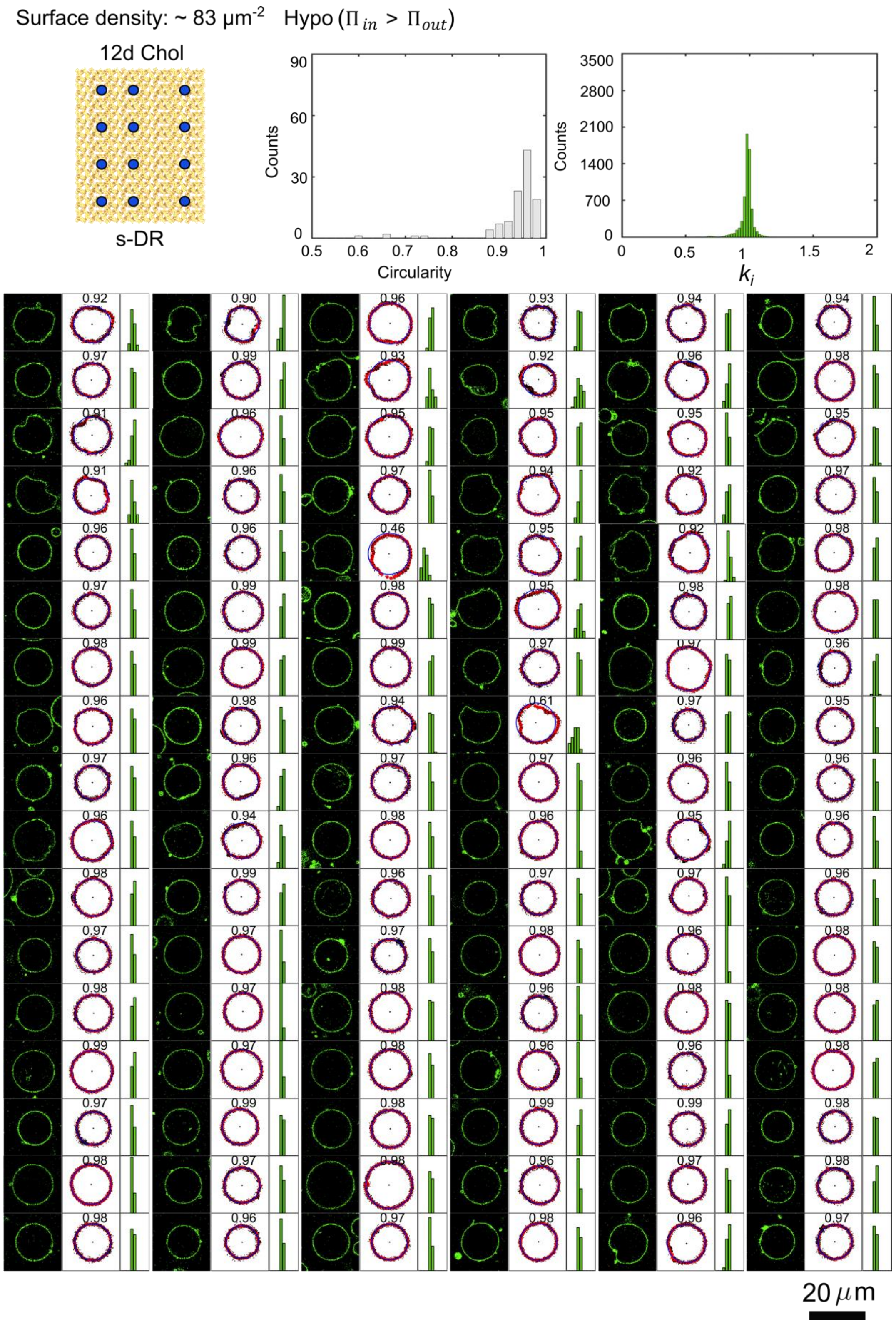

Data S22. 12d Chol s-DR-bound GUVs after hypoosmotic shocks. The deformation efficiency is ~30.4%.

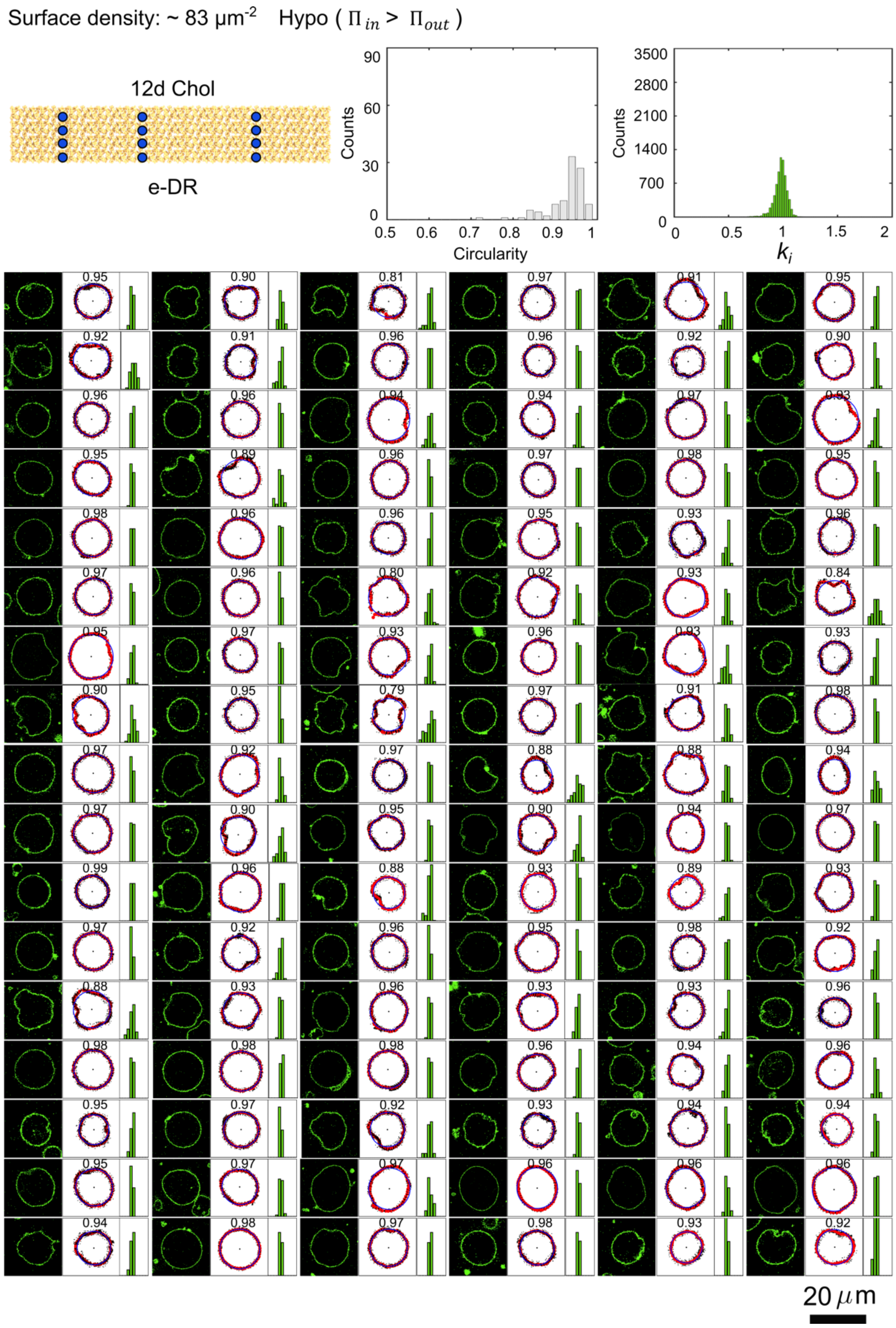

Data S23. 12d Chol e-DR-bound GUVs after hypoosmotic shocks. The deformation efficiency is ~73.1%.

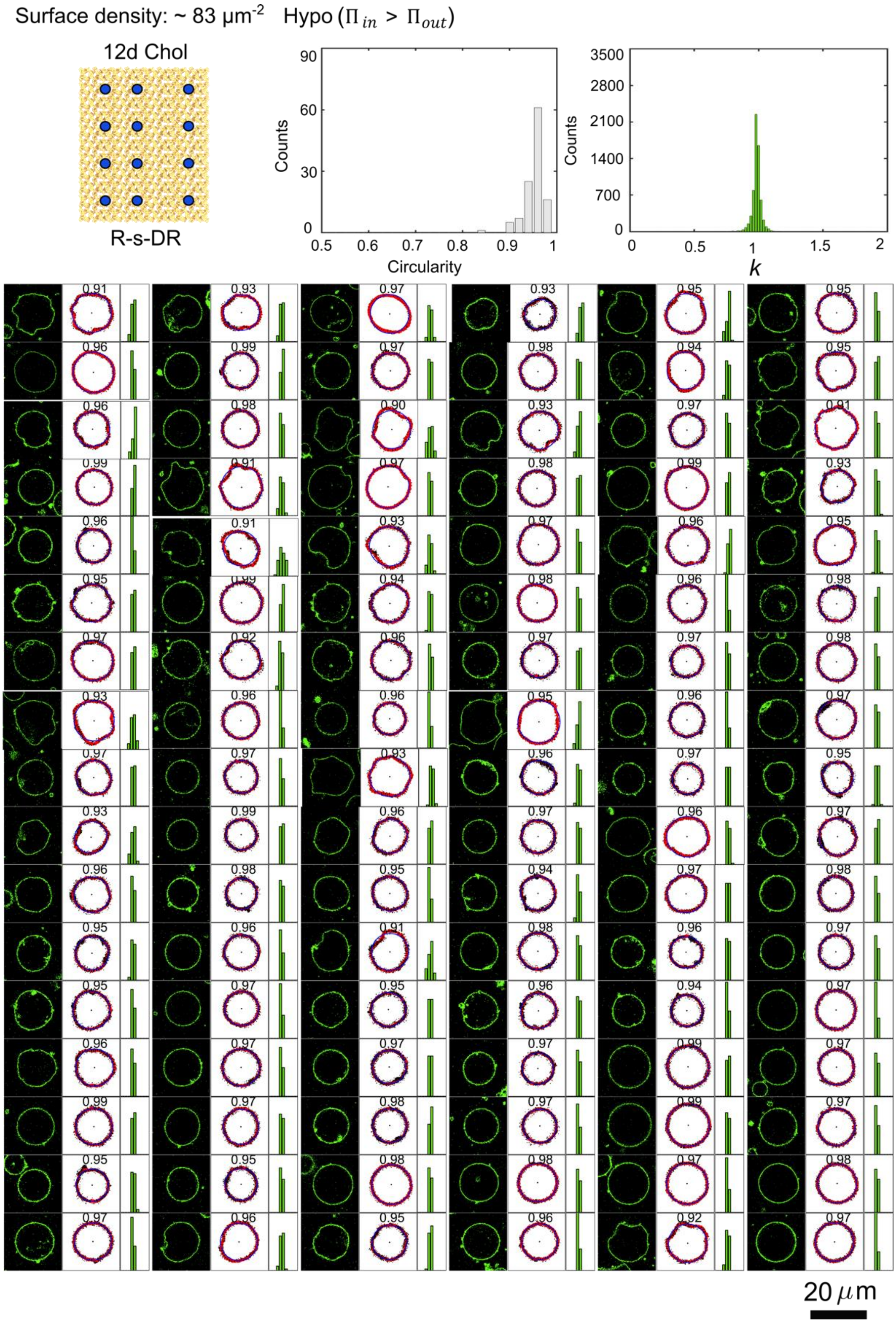

Data S24. 12d Chol R-s-DR-bound GUVs after hypoosmotic shocks. The deformation efficiency is ~38.7%.

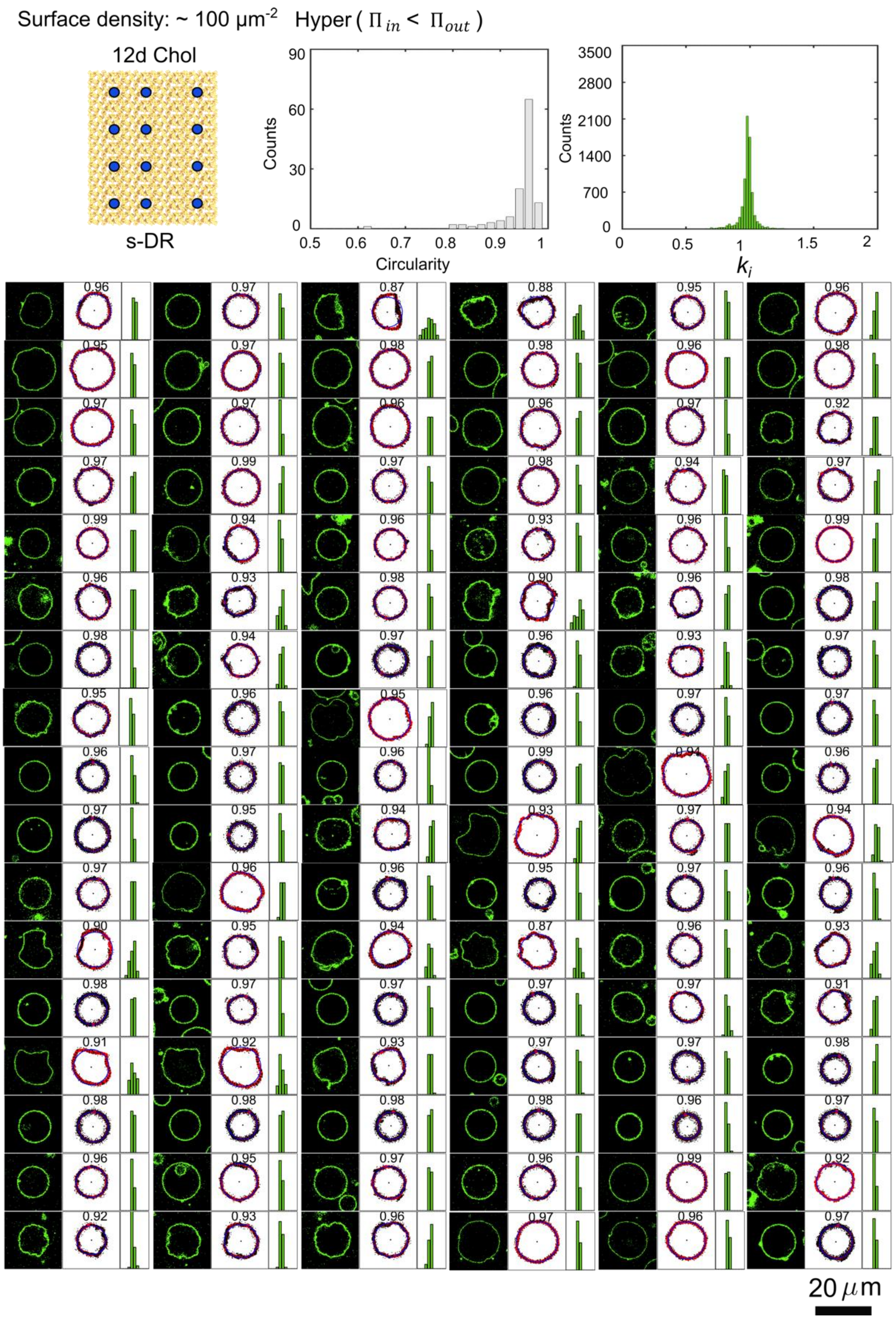

Data S25. 12d Chol s-DR-bound GUVs after hyperosmotic shocks. The deformation efficiency is ~40.3%.

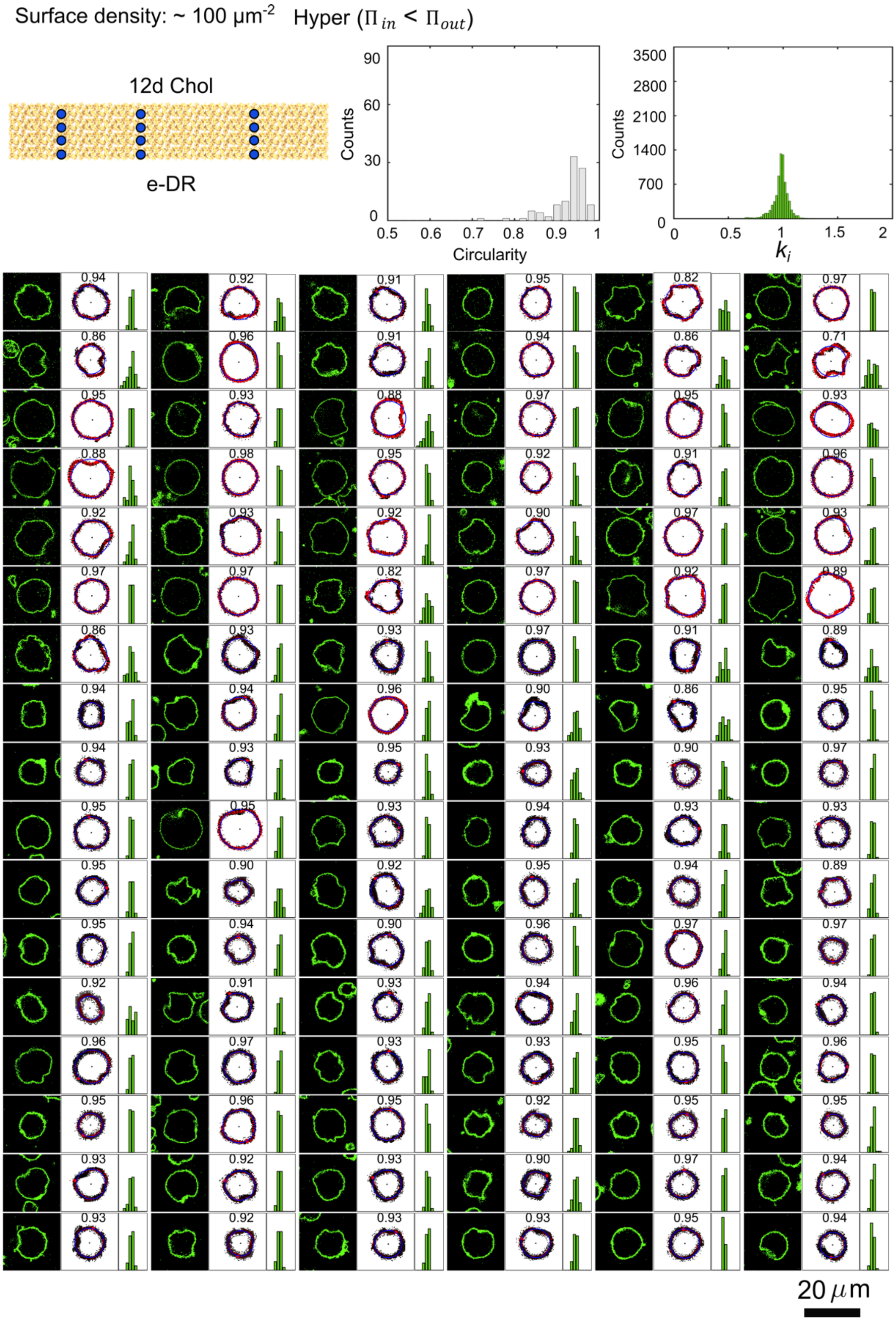

Data S26. 12d Chol e-DR-bound GUVs after hyperosmotic shocks. The deformation efficiency is ~92.6%.

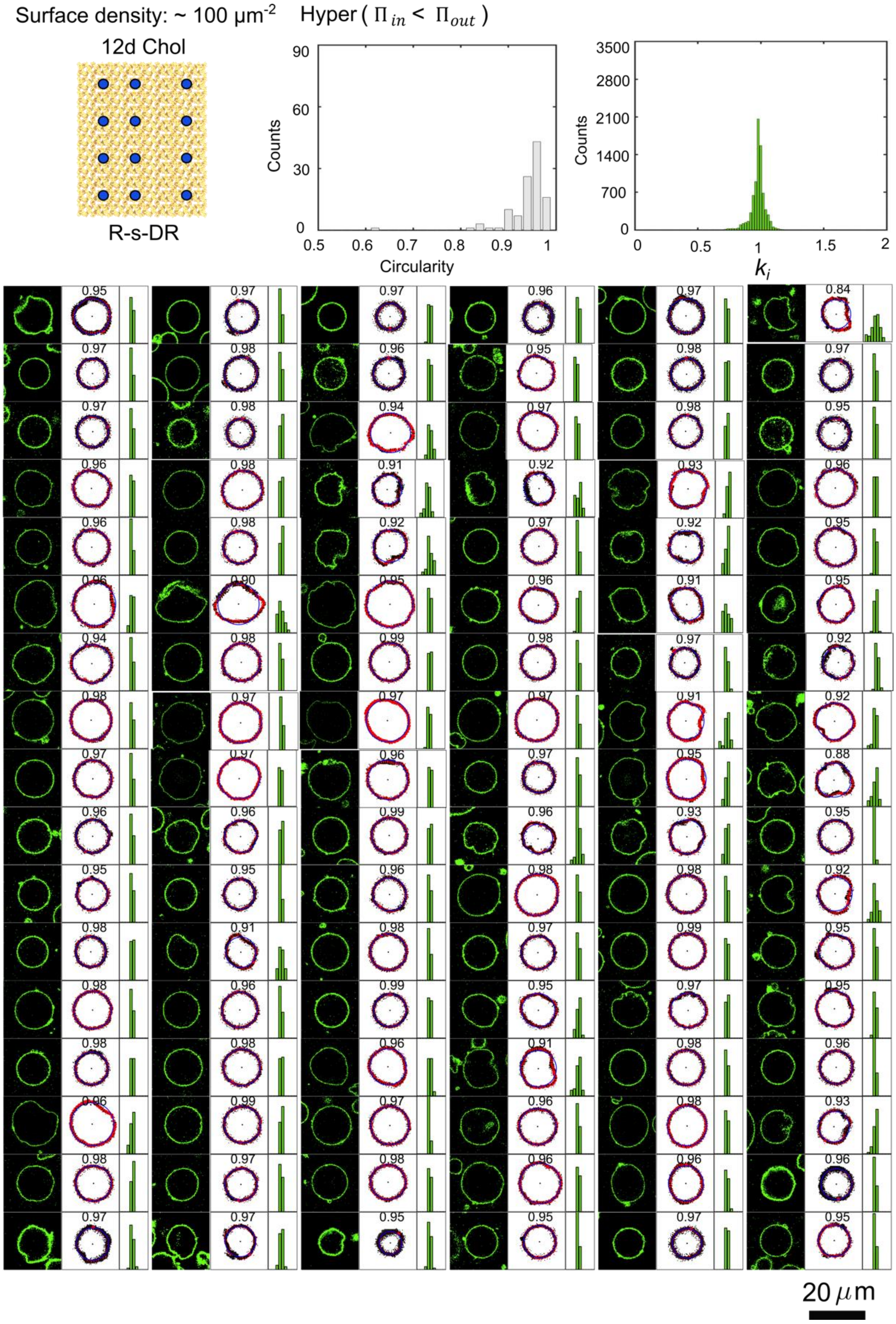

Data S27. 12d Chol R-s-DR-bound GUVs after hyperosmotic shocks. The deformation efficiency is ~47.3%.

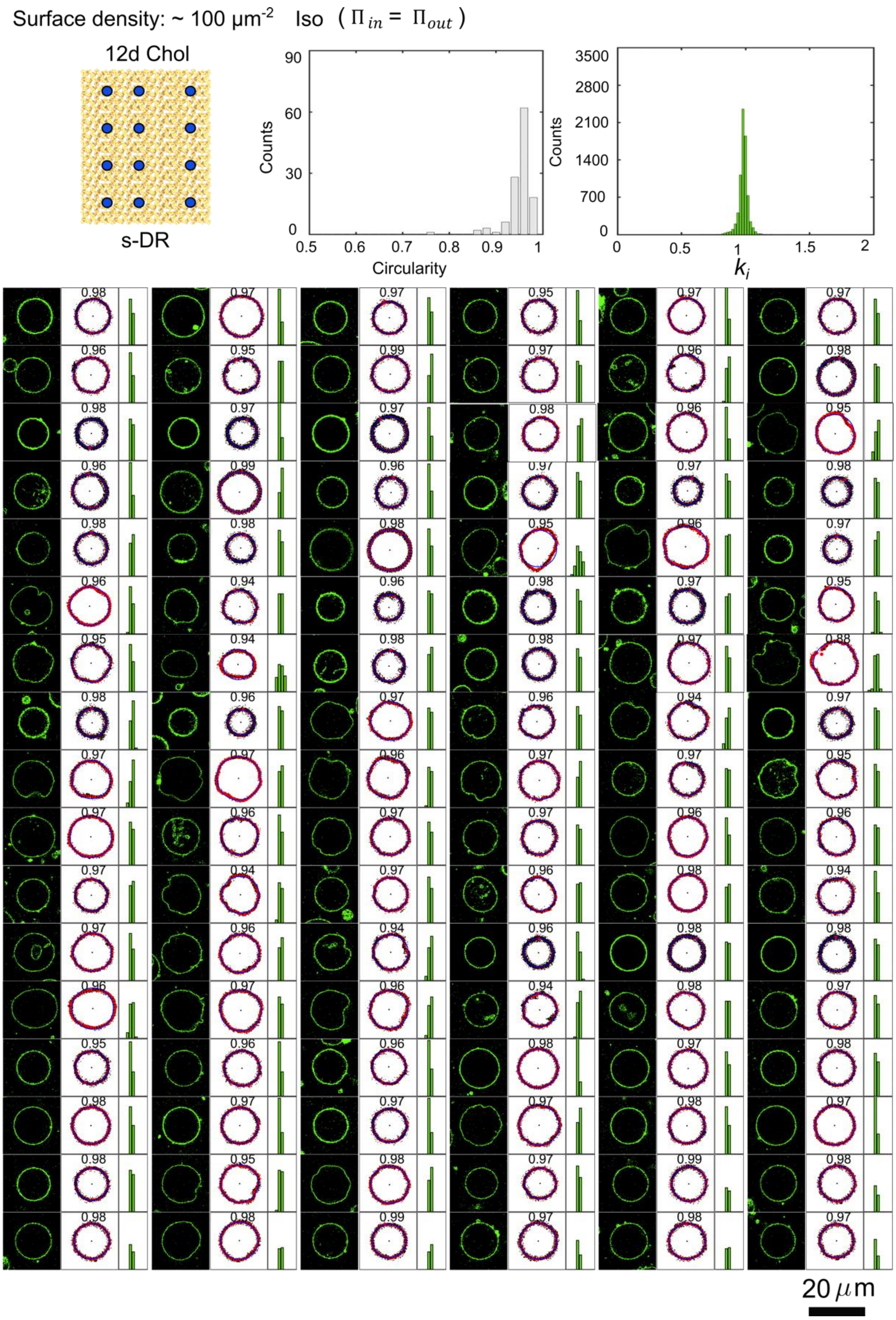

Data S28. 12d Chol s-DR-bound GUVs in isoosmotic buffer. The deformation efficiency is ~22.3%.

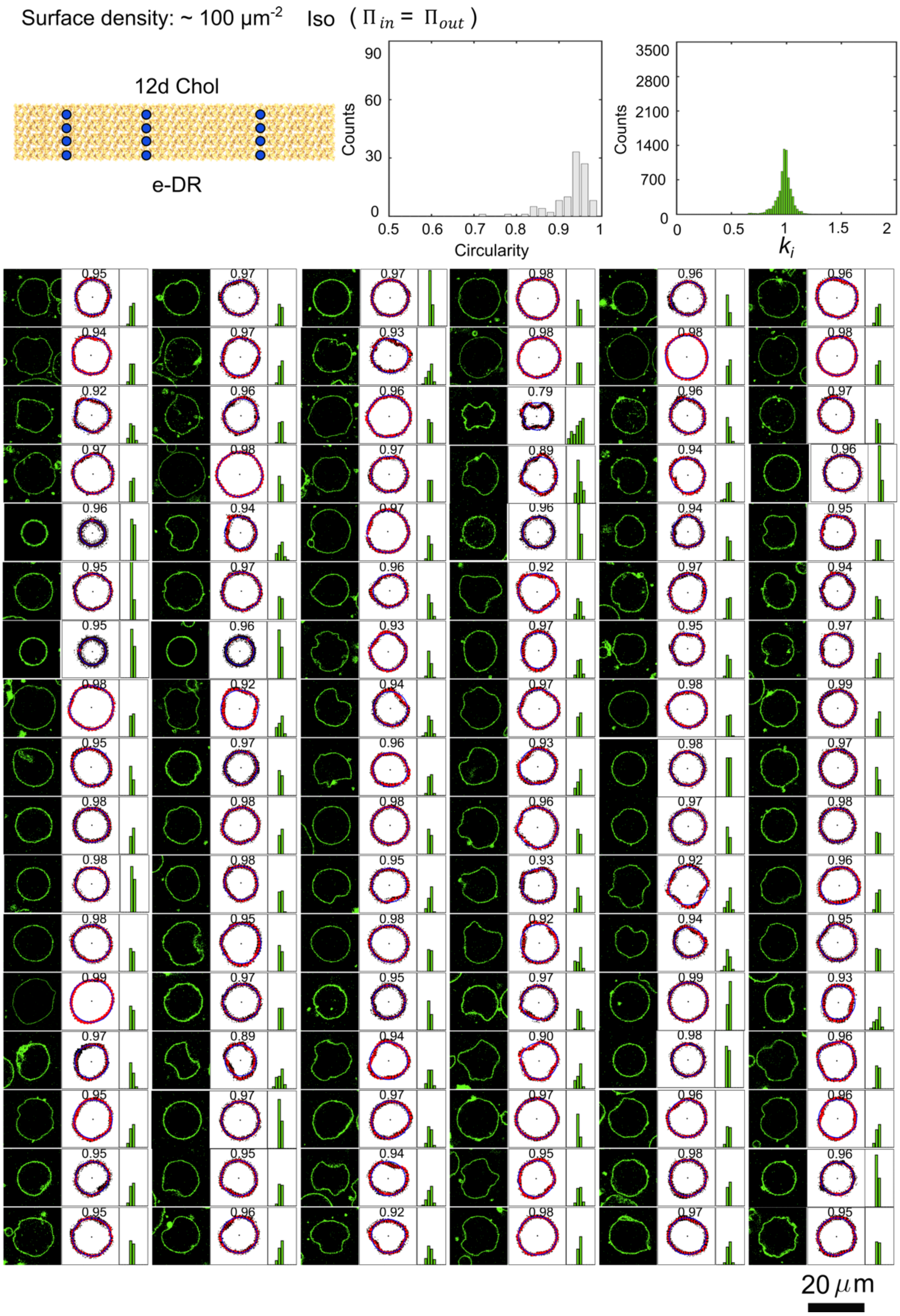

Data S29. 12d Chol e-DR-bound GUVs in isoosmotic buffer. The deformation efficiency is ~75.9%.

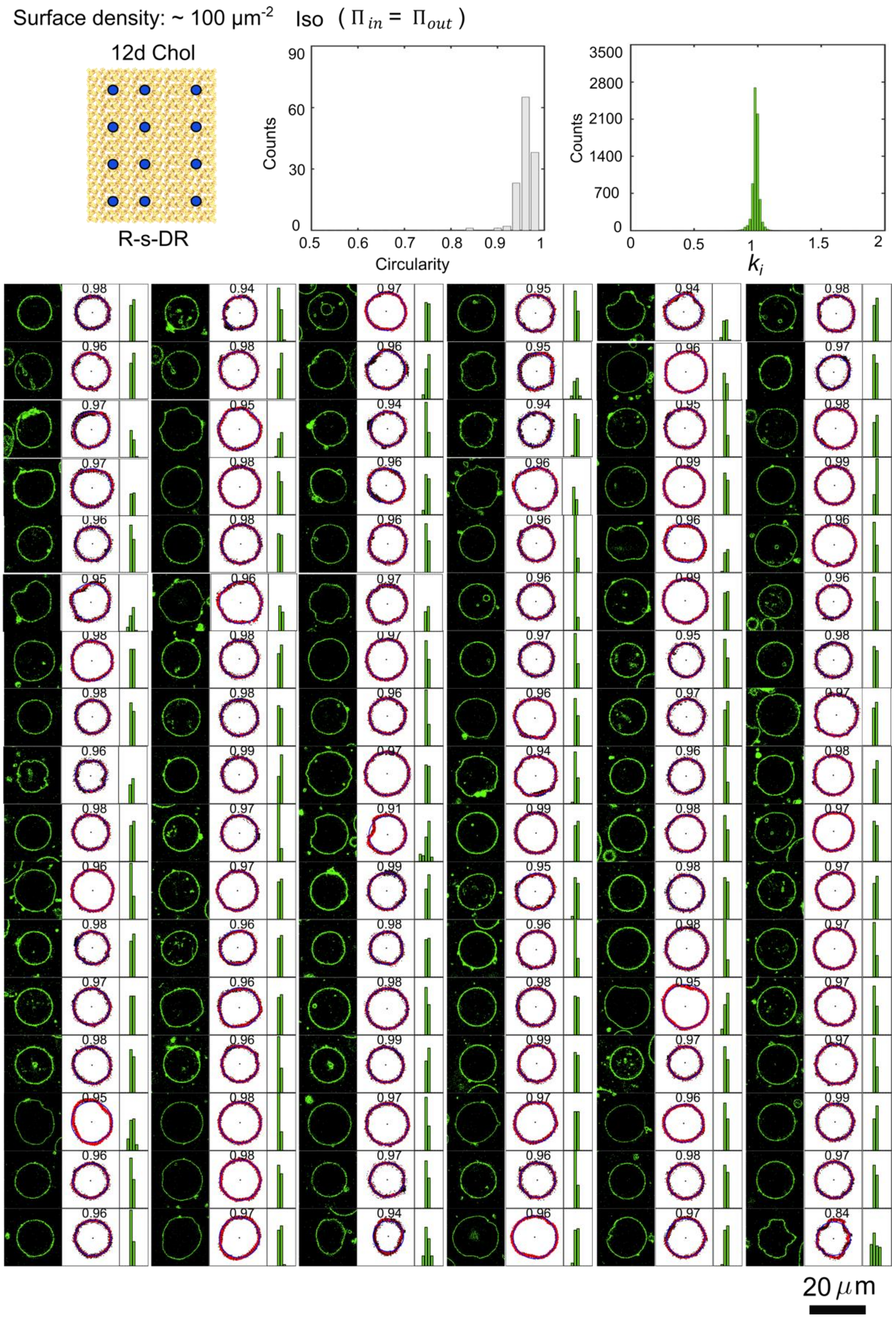

Data S30. 12d Chol R-s-DR-bound GUVs in isoosmotic buffer. The deformation efficiency is ~31.1%.

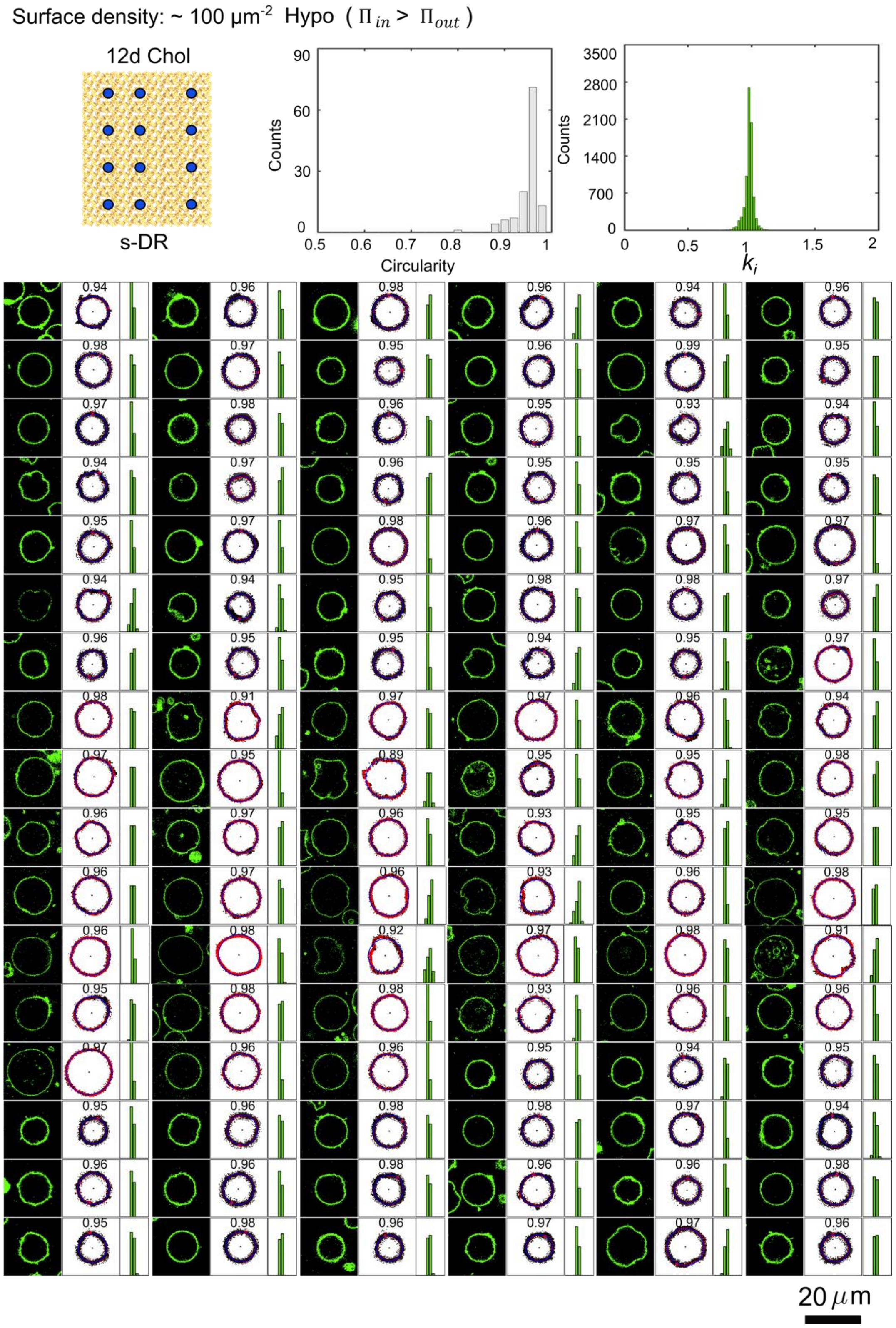

Data S31. 12d Chol s-DR-bound GUVs after hypoosmotic shocks. The deformation efficiency is ~31.7%.

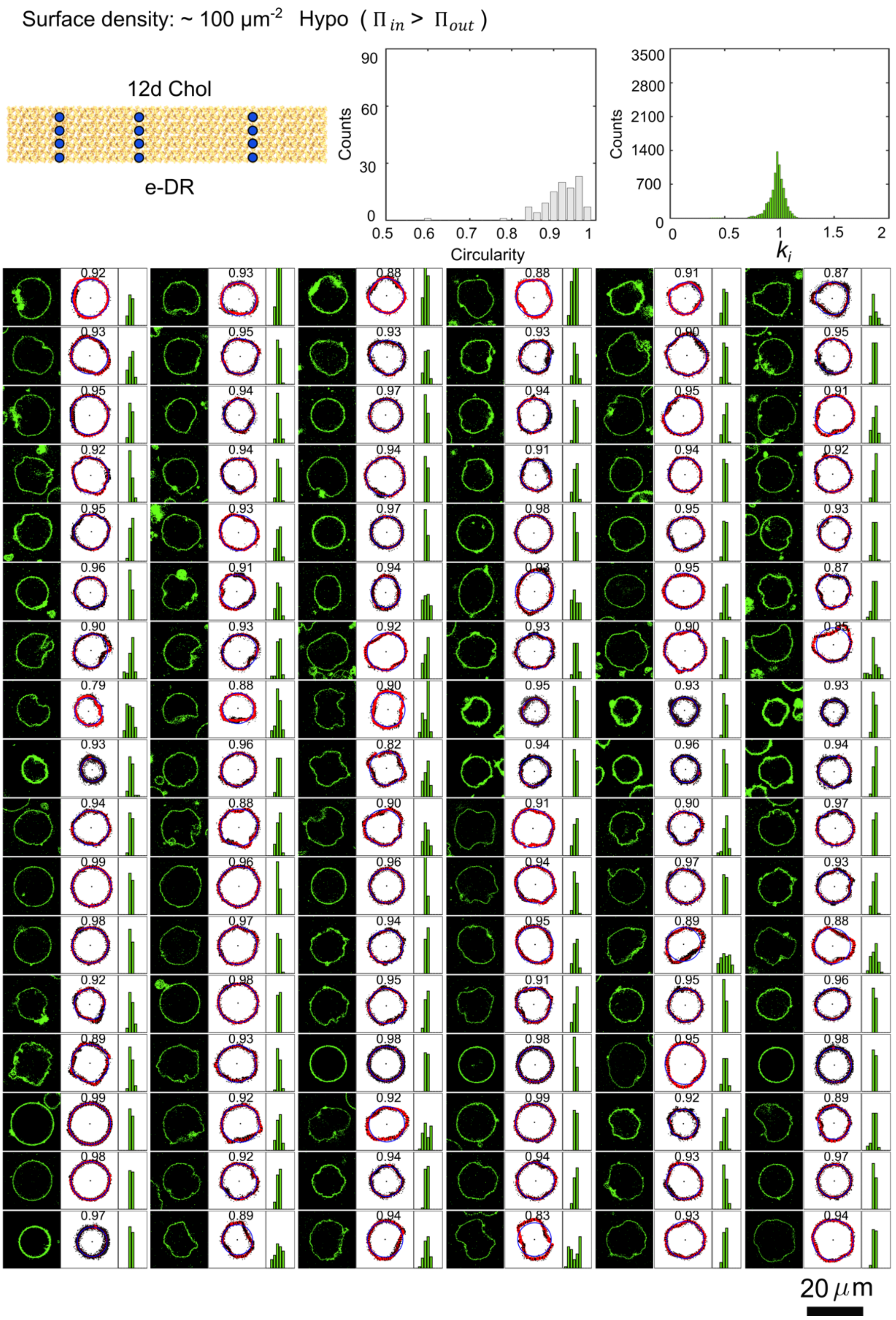

Data S32. 12d Chol e-DR-bound GUVs after hypoosmotic shocks. The deformation efficiency is ~74.4%.

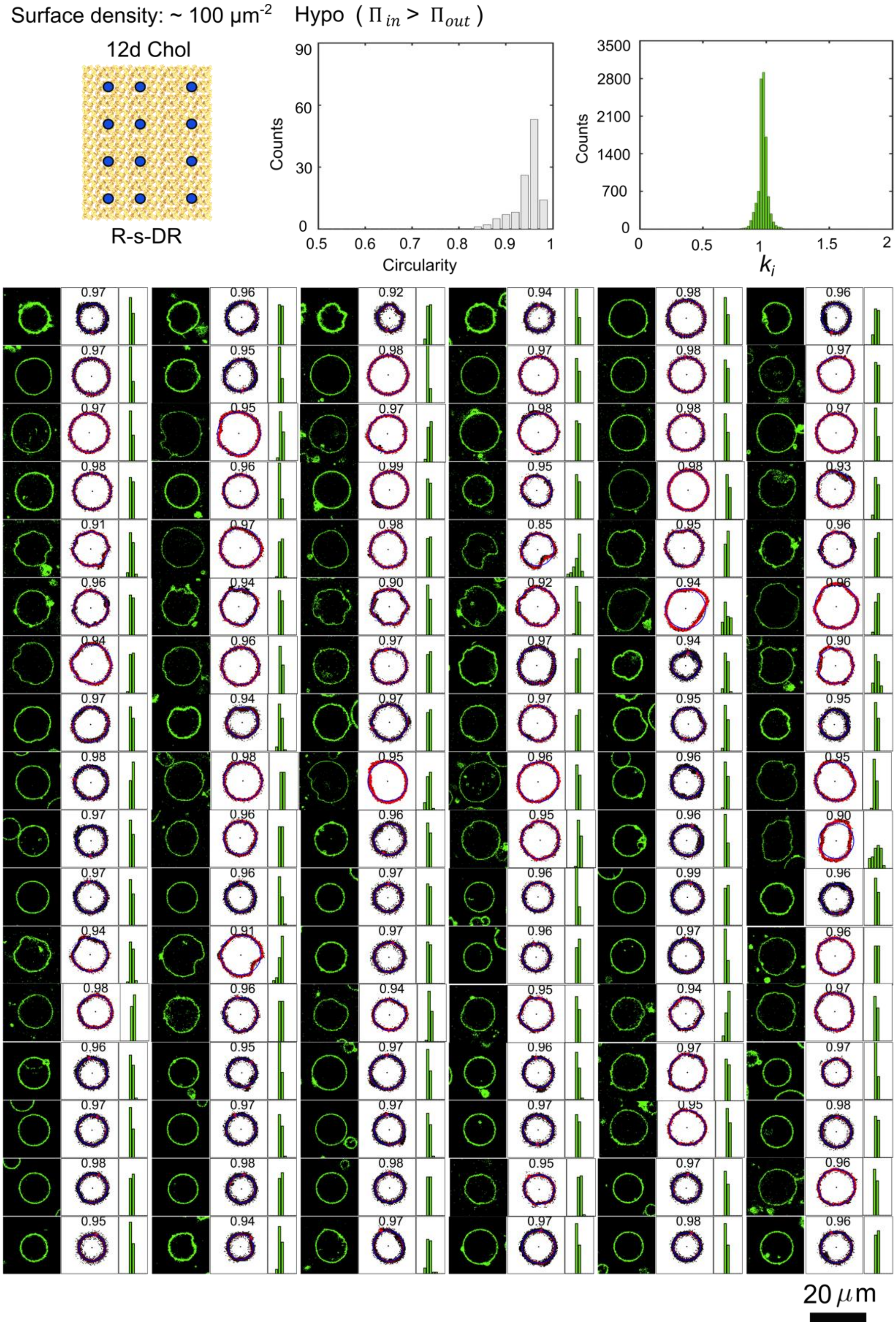

Data S33. 12d Chol R-s-DR-bound GUVs after hypoosmotic shocks. The deformation efficiency is ~44%.

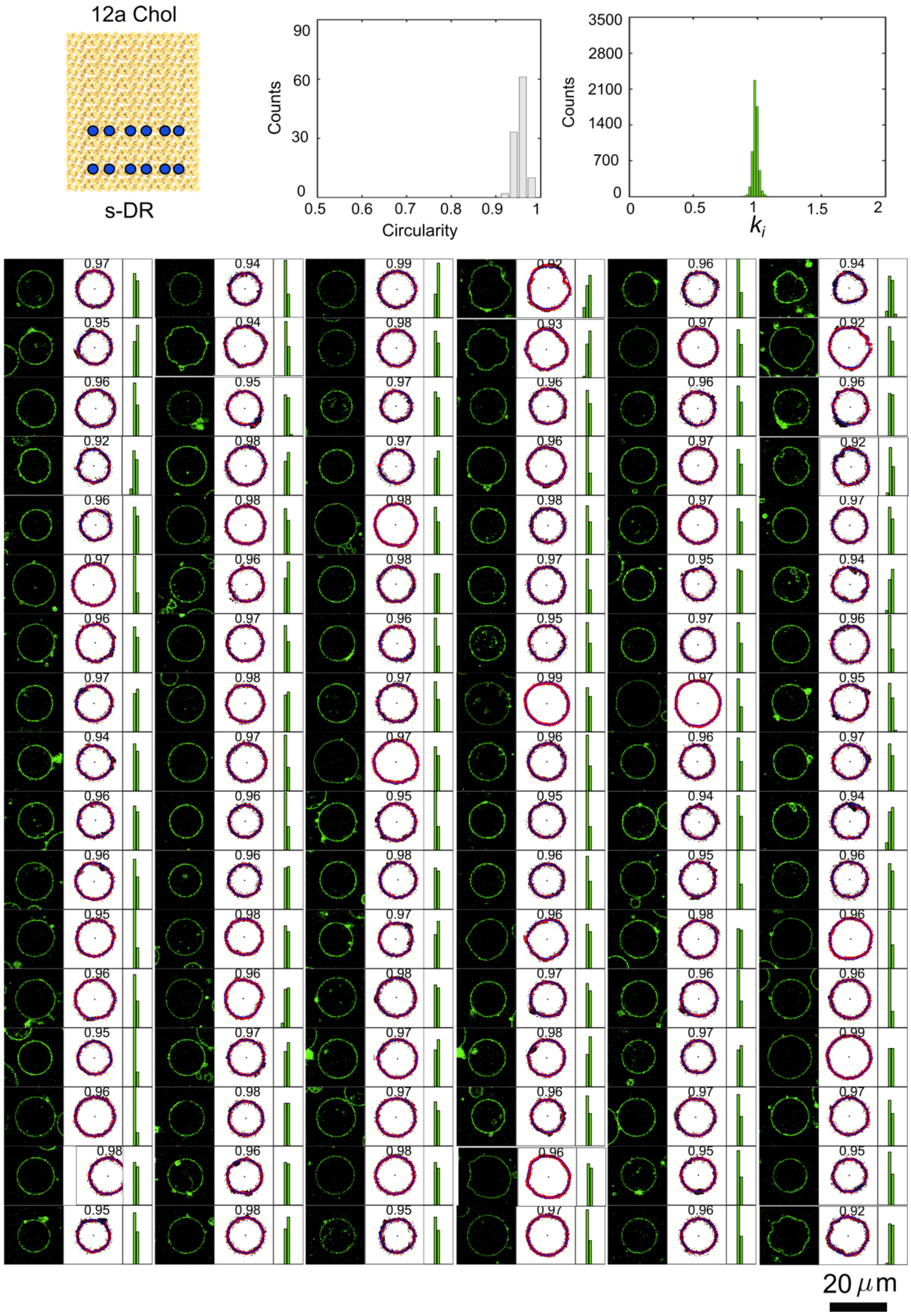

Data S34. 12a Chol s-DR-bound GUVs in isoosmotic buffer. The deformation efficiency is ~13.2%.

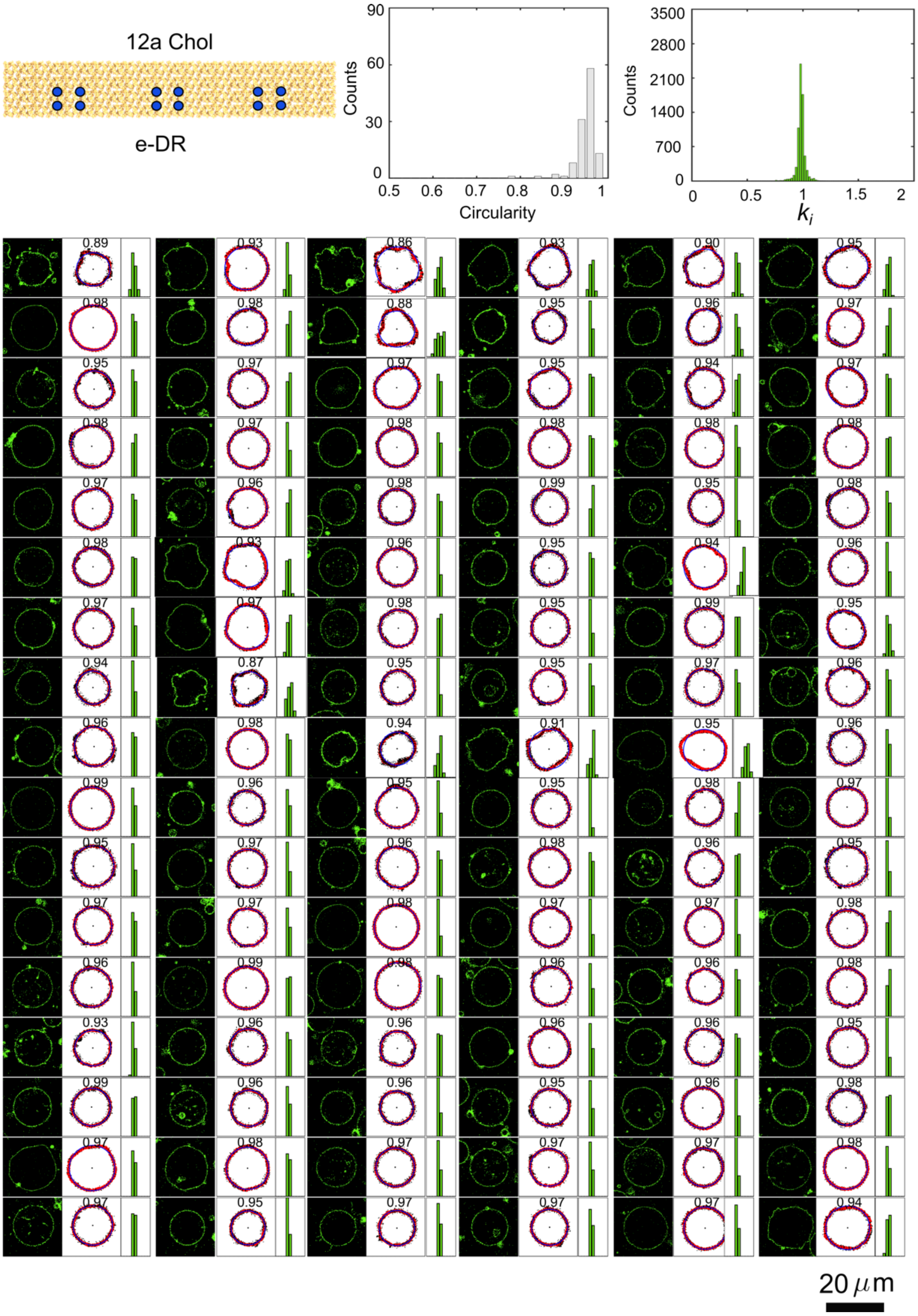

Data S35. 12a Chol e-DR-bound GUVs in isoosmotic buffer. The deformation efficiency is ~26.1%.

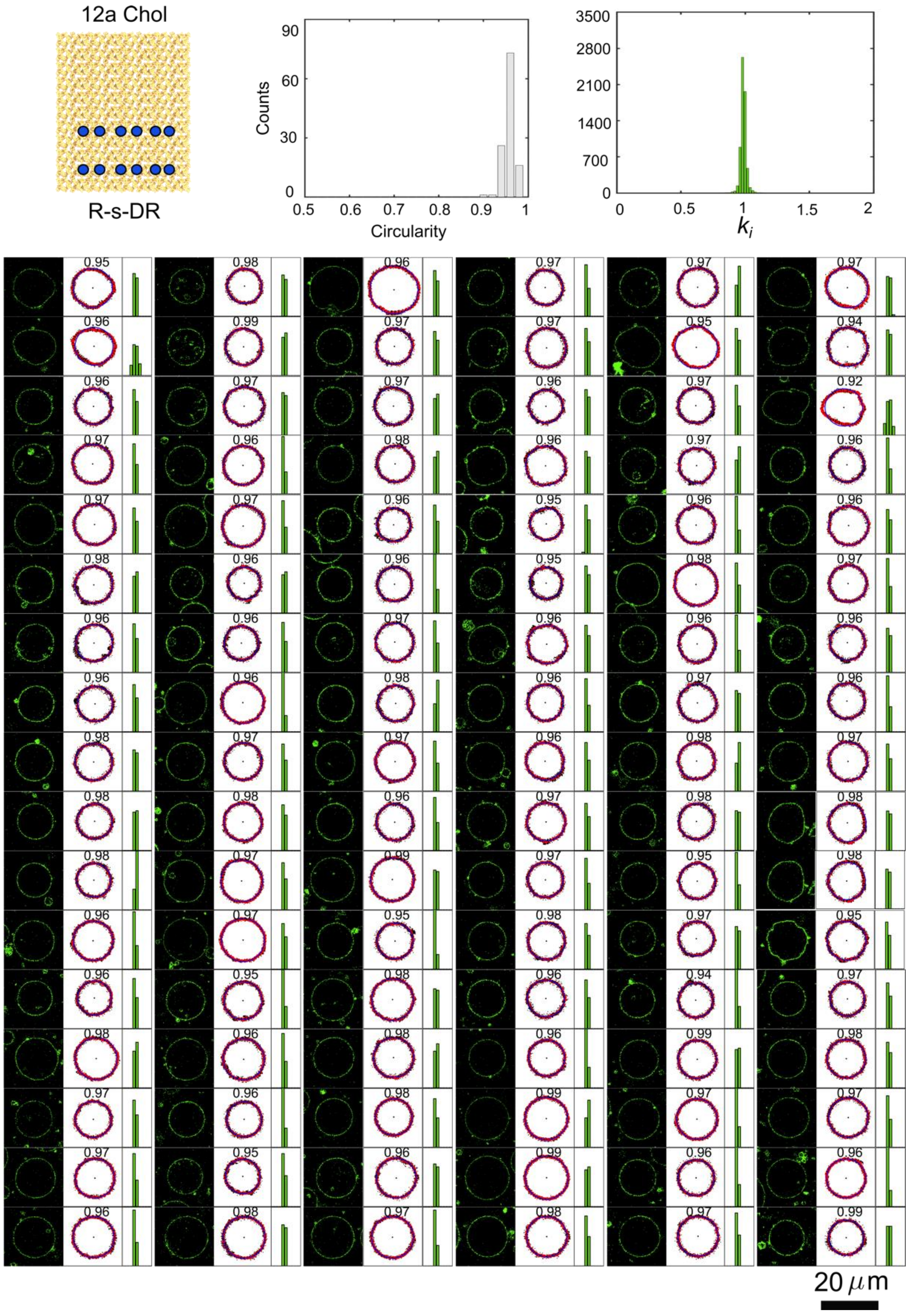

Data S36. 12a Chol R-s-DR-bound GUVs in isoosmotic buffer. The deformation efficiency is ~9.4%.

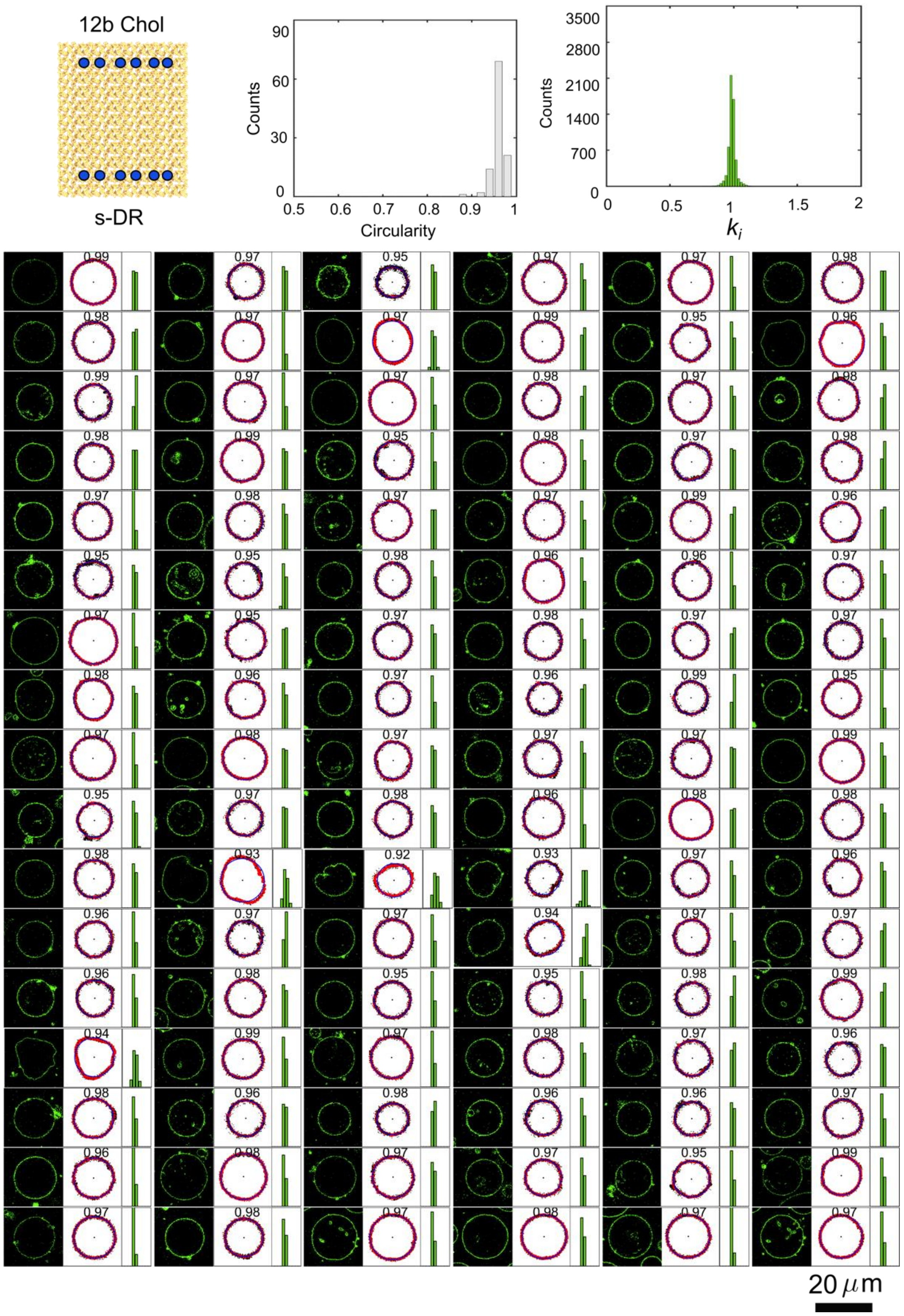

Data S37. 12b Chol s-DR-bound GUVs in isoosmotic buffer. The deformation efficiency is ~15.7%.

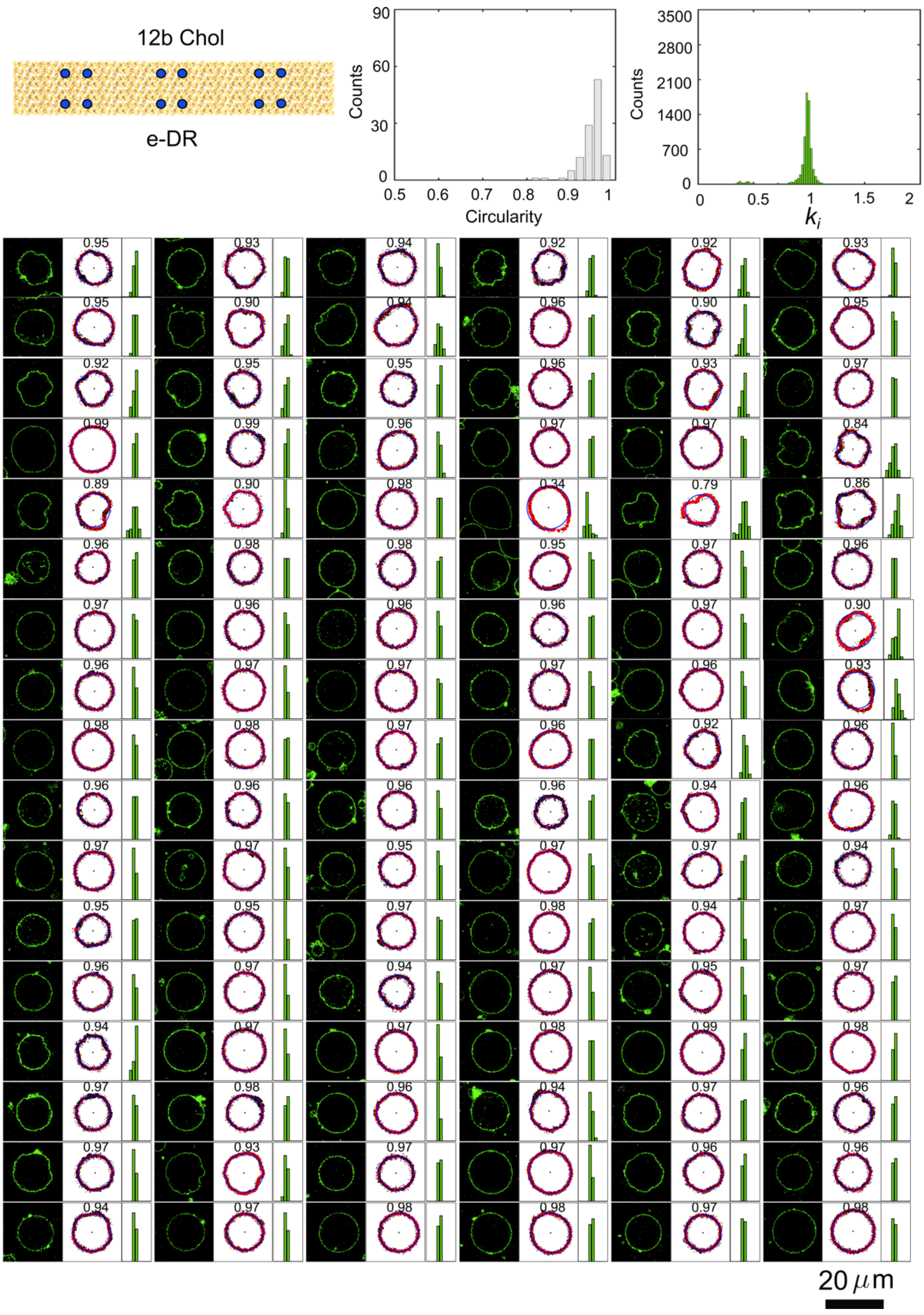

Data S38. 12b Chol e-DR-bound GUVs in isoosmotic buffer. The deformation efficiency of the GUVs is ~48.3%.

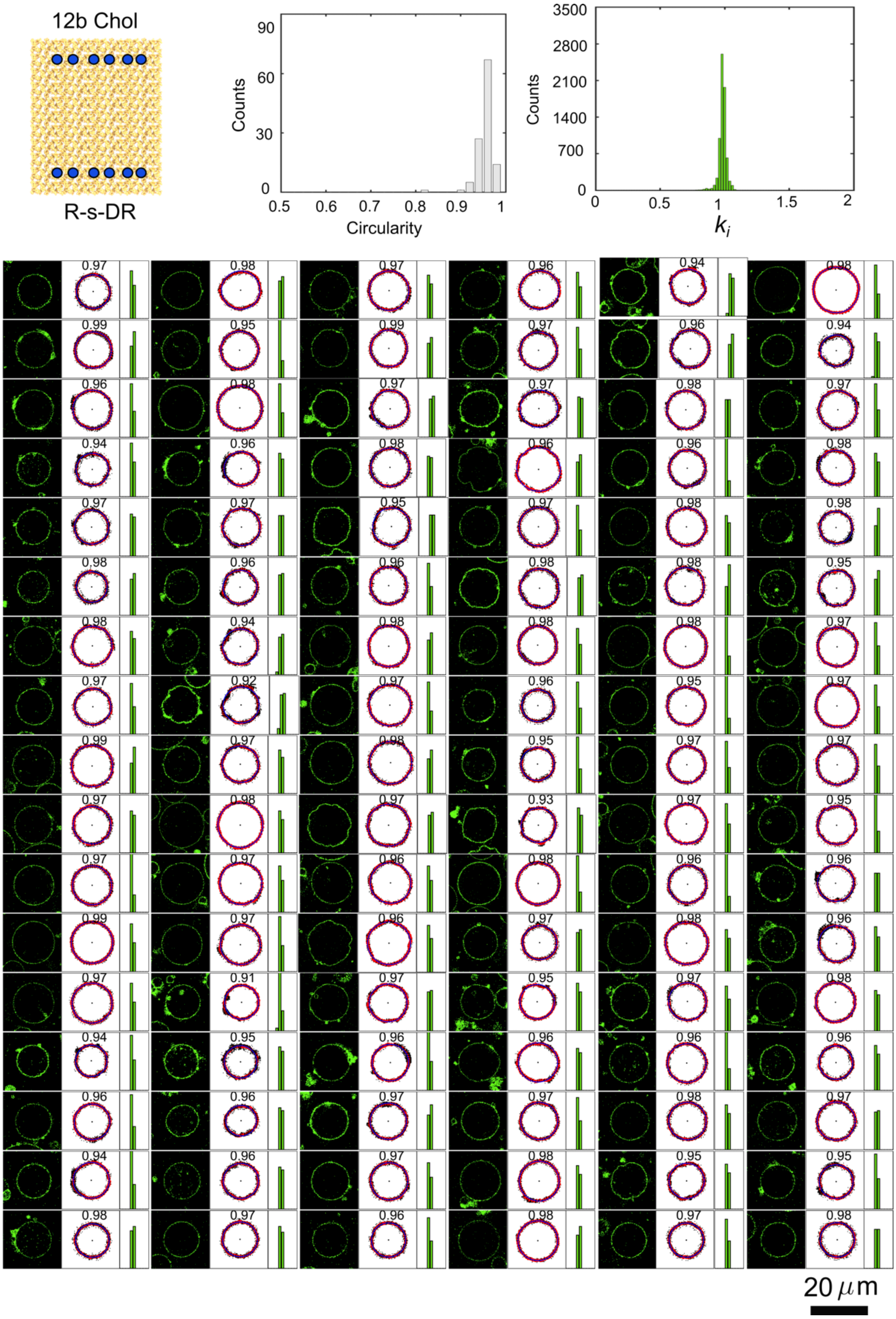

Data S39. 12b Chol R-s-DR-bound GUVs in isoosmotic buffer. The deformation efficiency is ~18.7%.

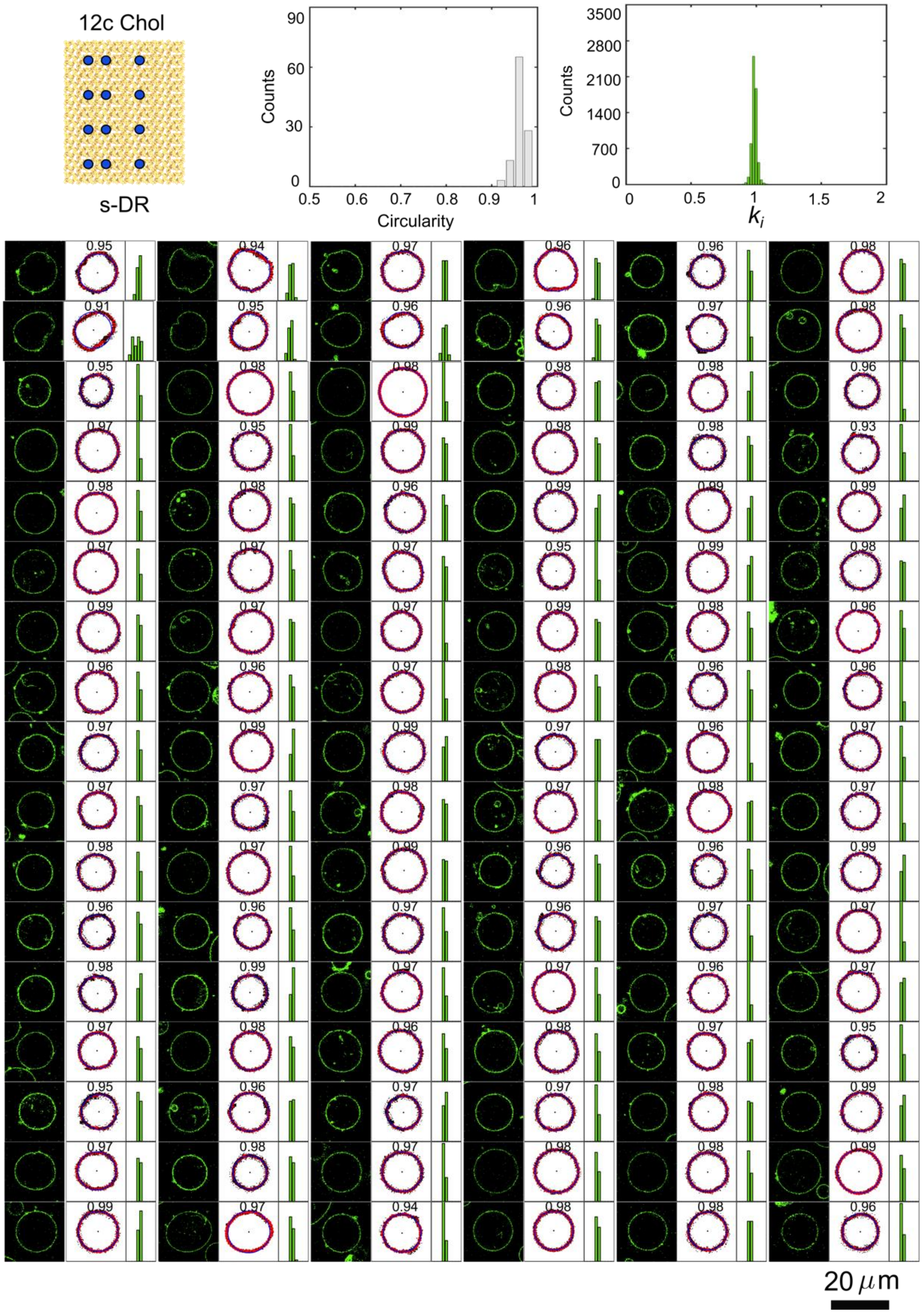

Data S40. 12c Chol s-DR-bound GUVs in isoosmotic buffer. The deformation efficiency is ~9.9%.

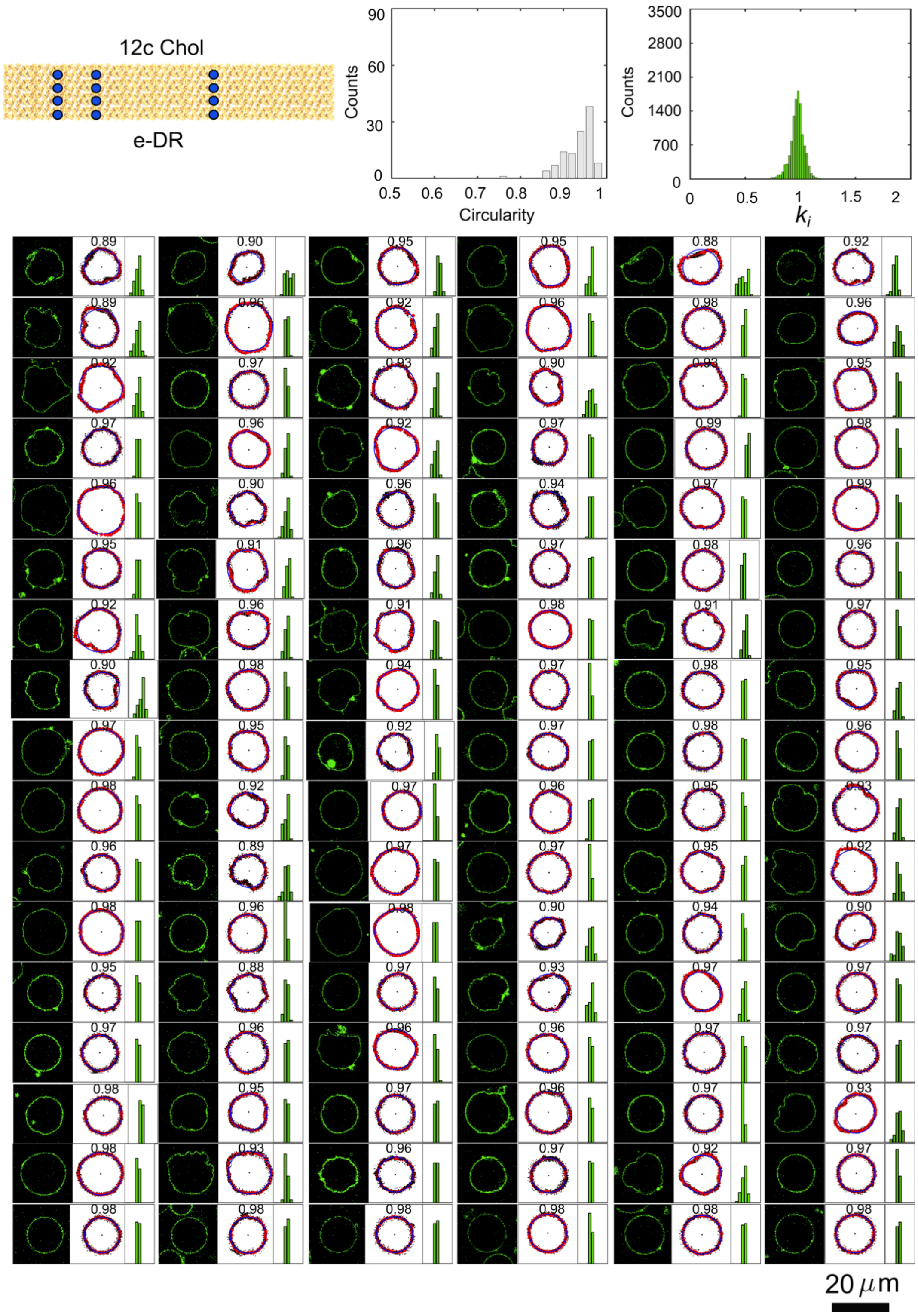

Data S41. 12c Chol e-DR-bound GUVs in isoosmotic buffer. The deformation efficiency is ~64.5%.

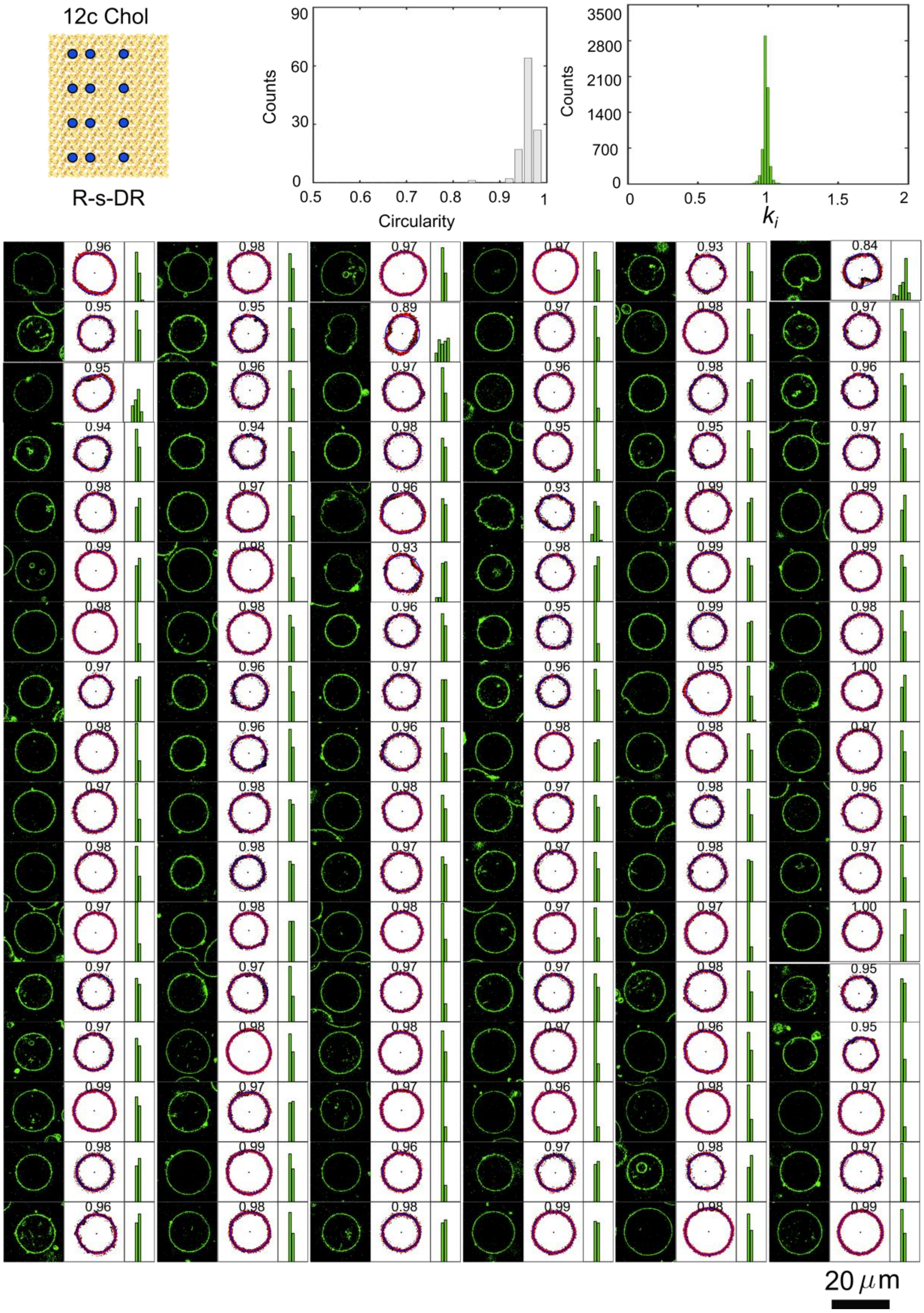

Data S42. 12c Chol R-s-DR-bound GUVs in isoosmotic buffer. The deformation efficiency is ~11%.

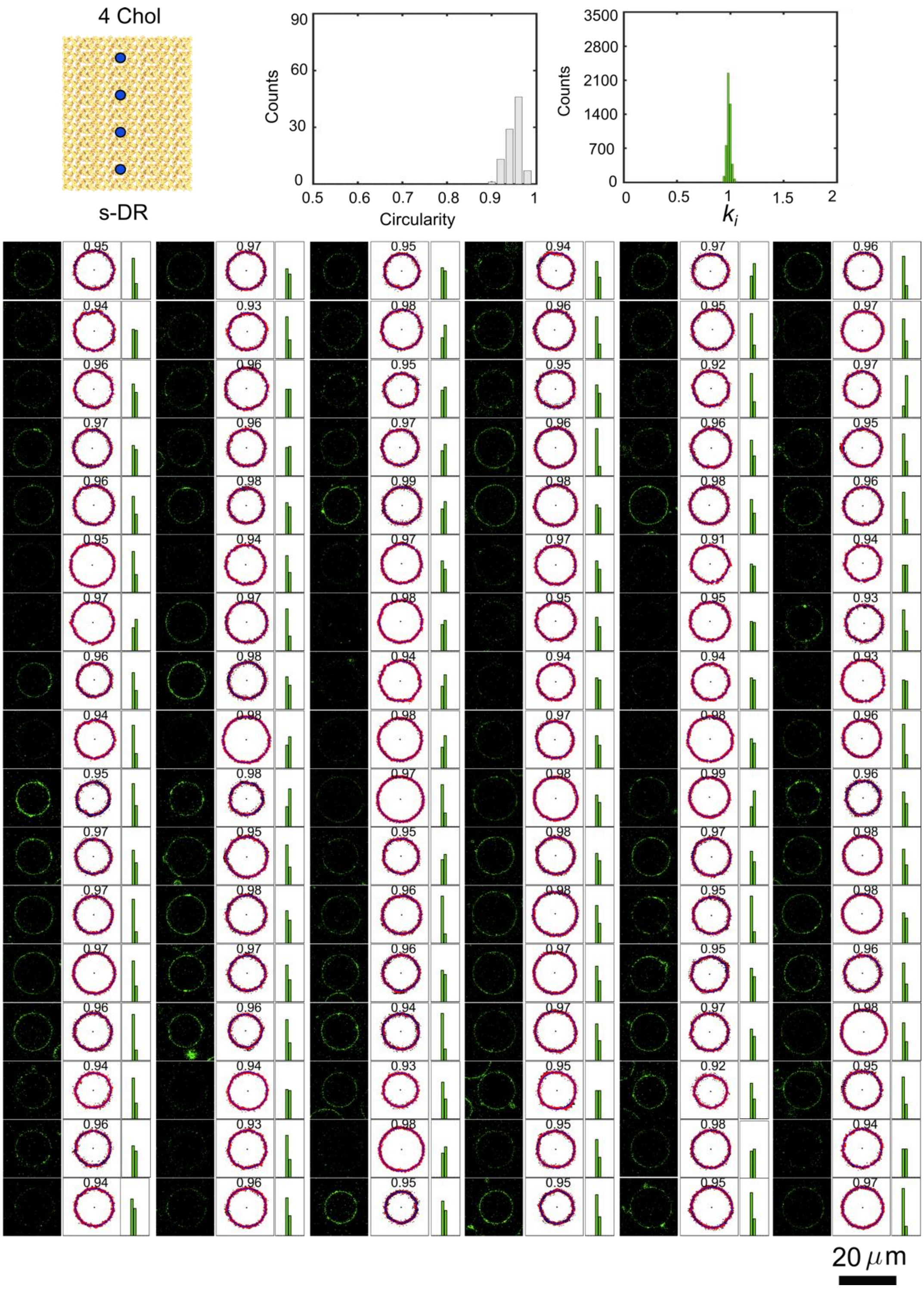

Data S43. 4 Chol s-DR-bound GUVs in isoosmotic buffer. The deformation efficiency is ~1%.

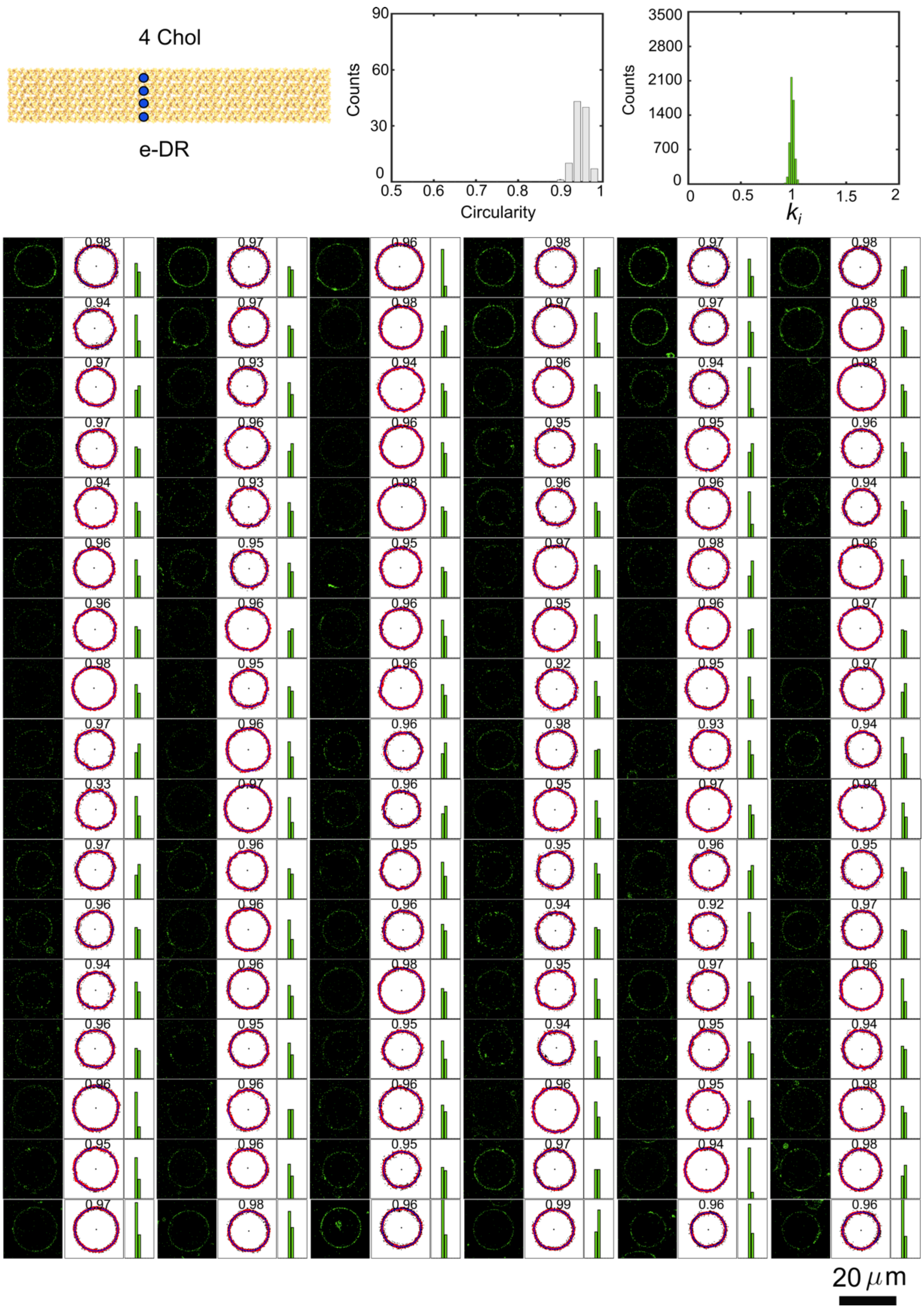

Data S44. 4 Chol e-DR-bound GUVs in isoosmotic buffer. The deformation efficiency is ~2.9%.

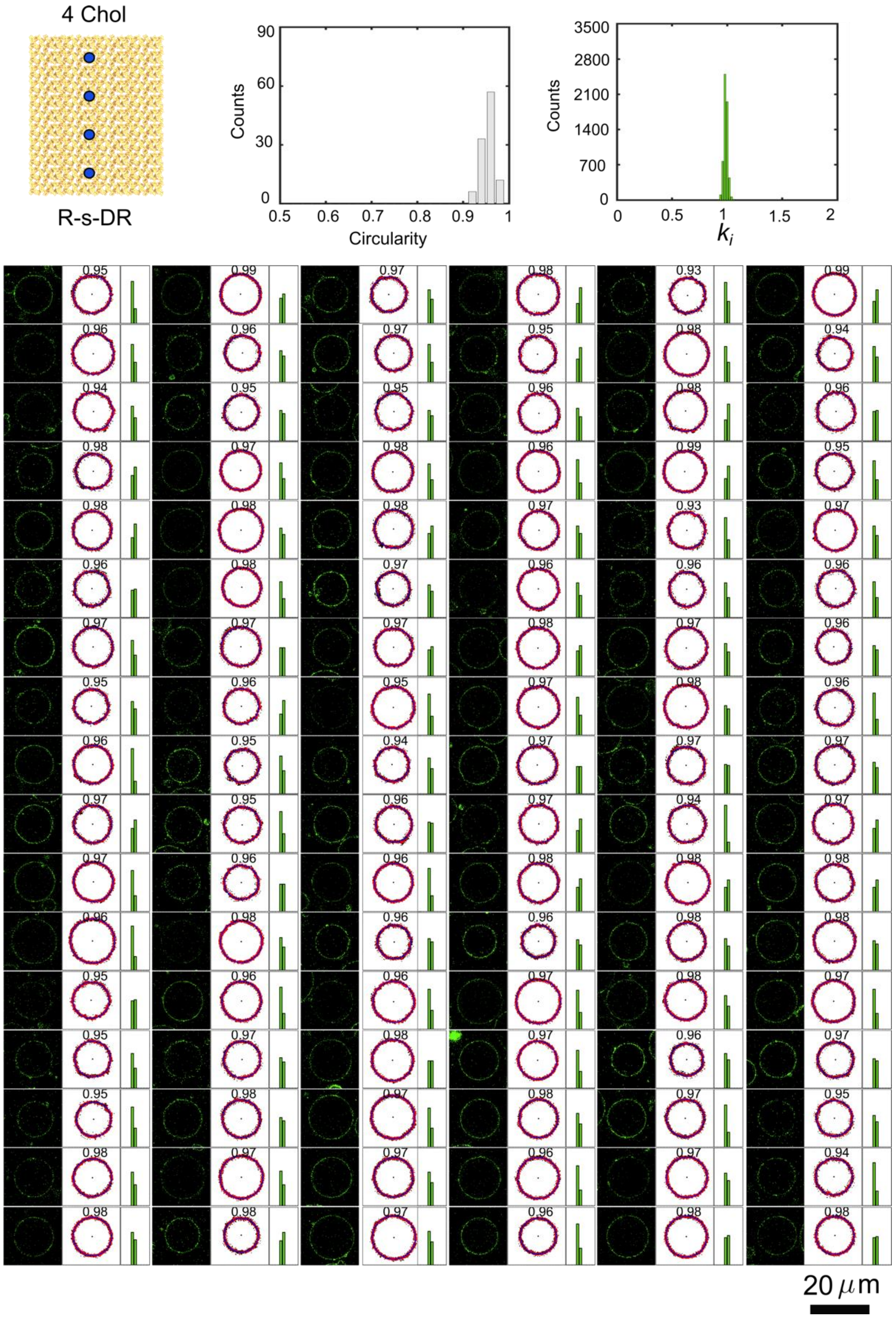

Data S45. 4 Chol R-s-DR-bound GUVs in isoosmotic buffer. The deformation efficiency is ~0%.

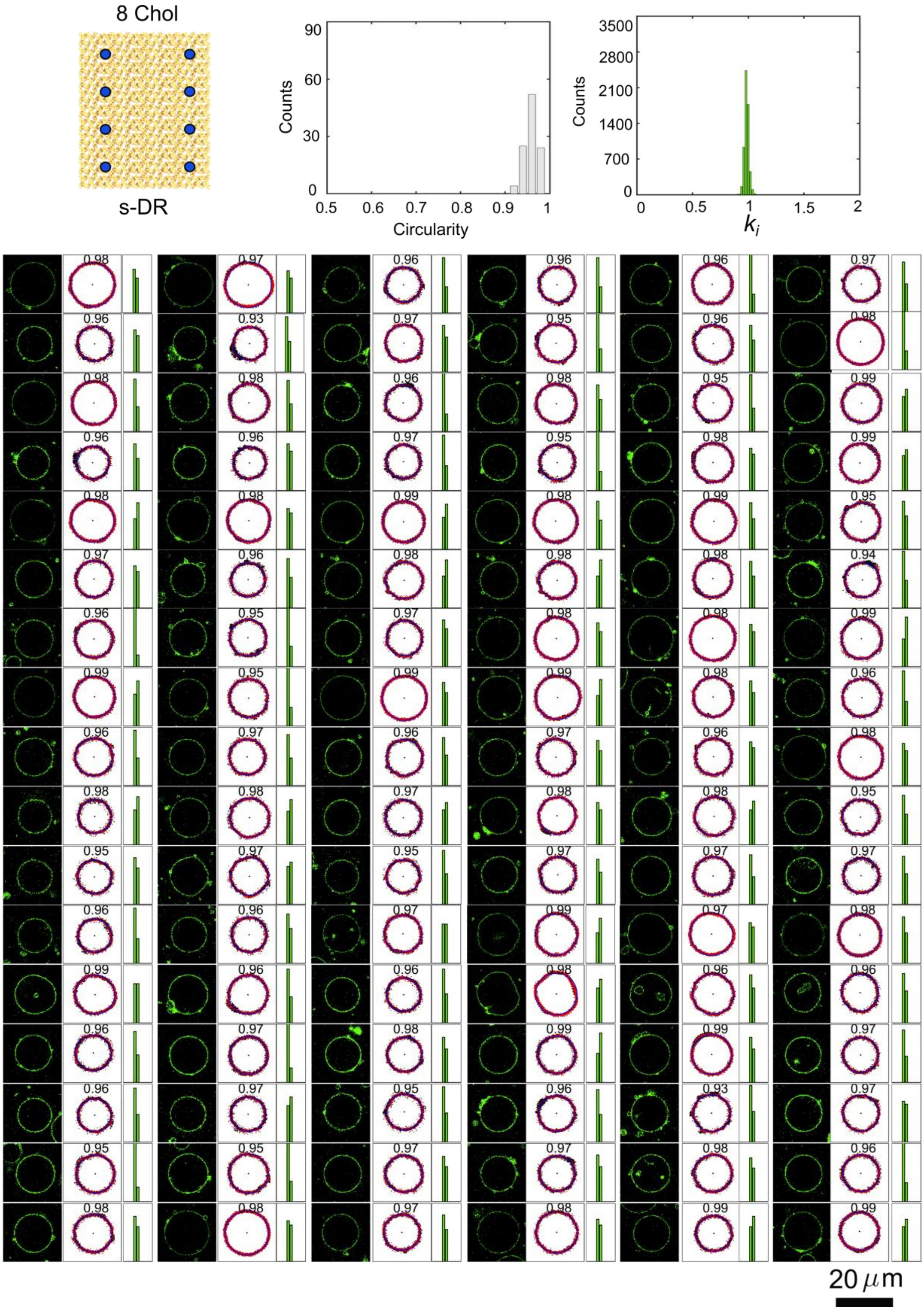

Data S46. 8 Chol s-DR-bound GUVs in isoosmotic buffer. The deformation efficiency is ~5.6%.

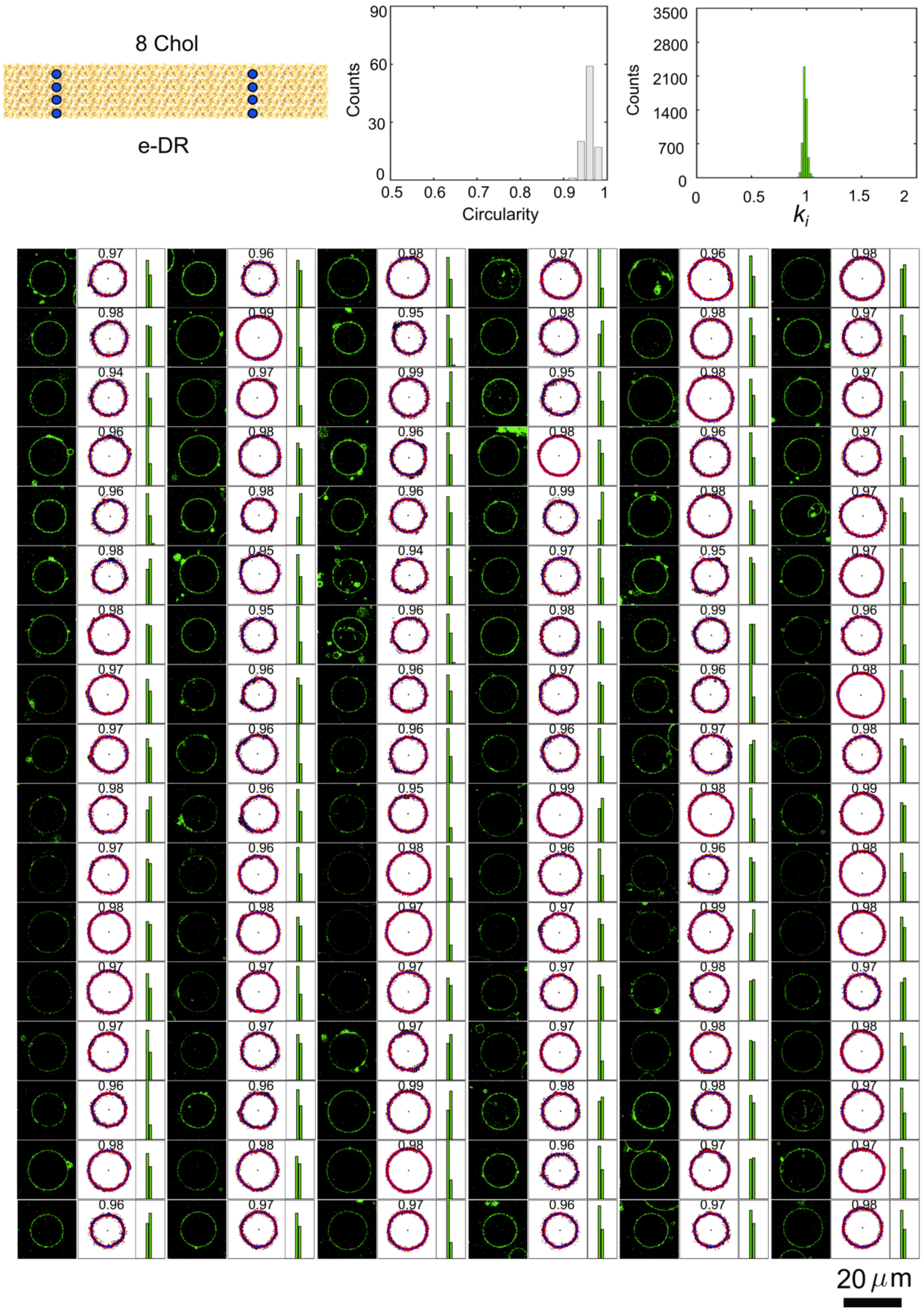

Data S47. 8 Chol e-DR-bound GUVs in isoosmotic buffer. The deformation efficiency is ~8.4%.

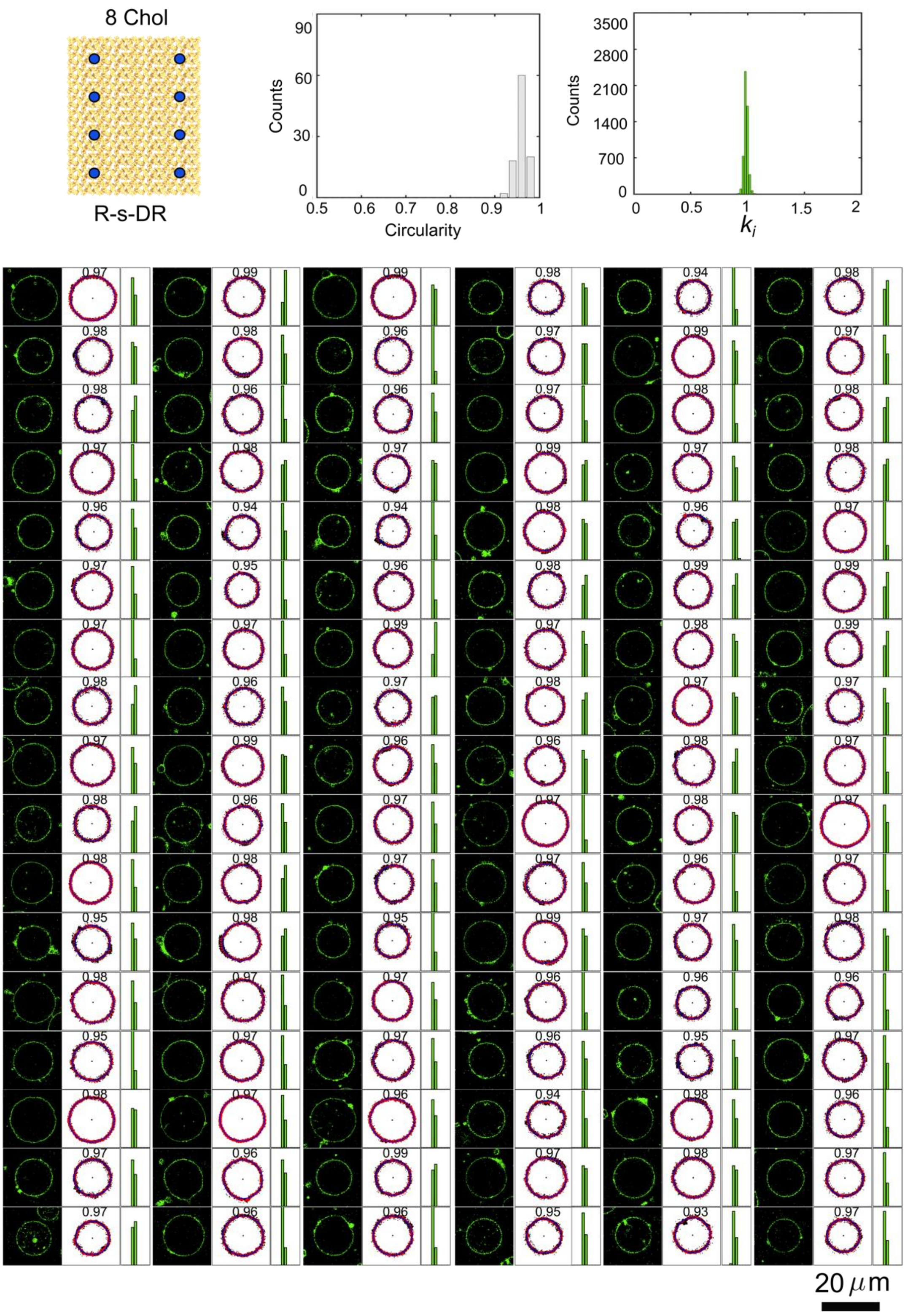

Data S48. 8 Chol R-s-DR-bound GUVs in isoosmotic buffer. The deformation efficiency is ~5.6%.

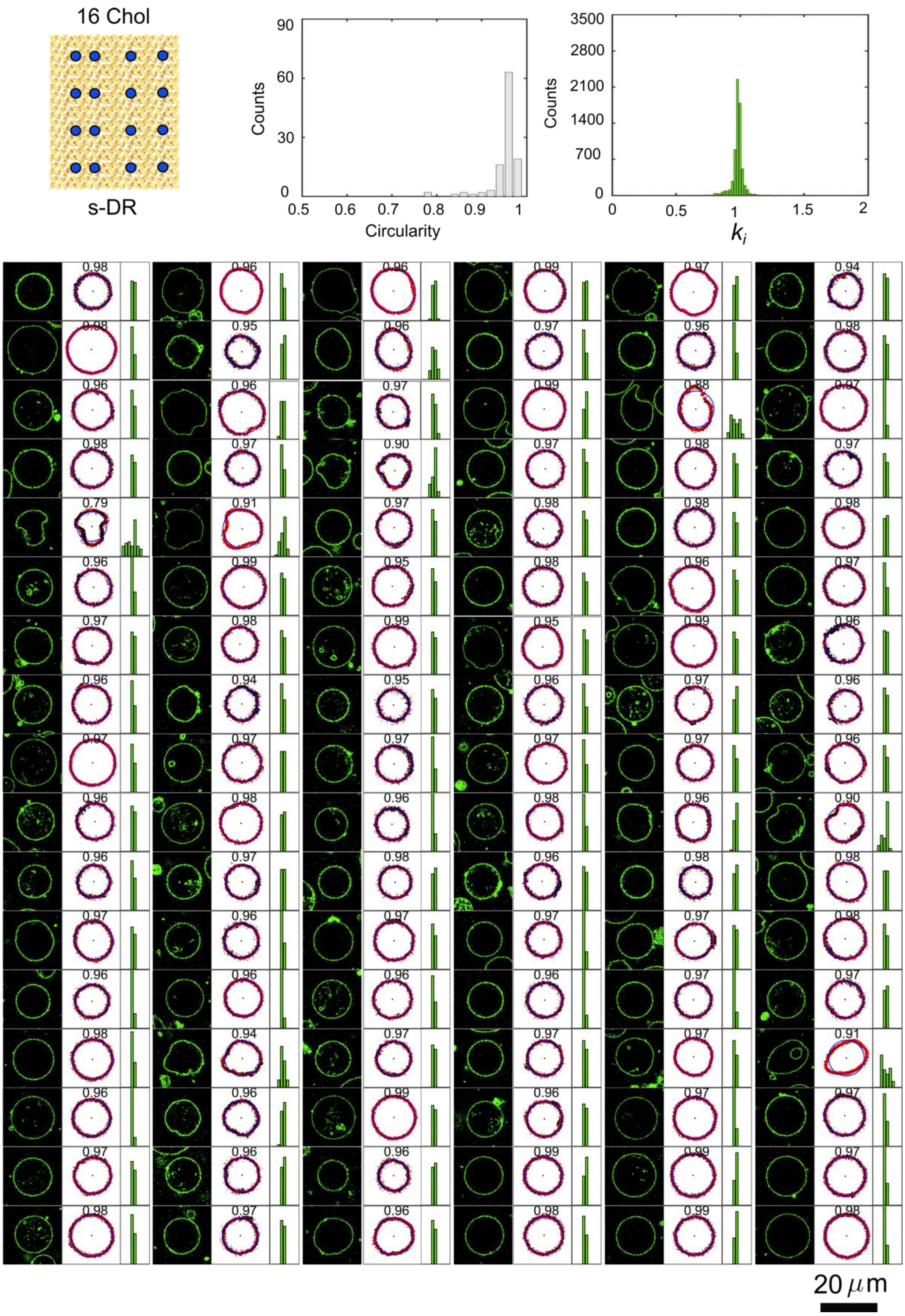

Data S49. 16 Chol s-DR-bound GUVs in isoosmotic buffer. The deformation efficiency is ~25.7%.

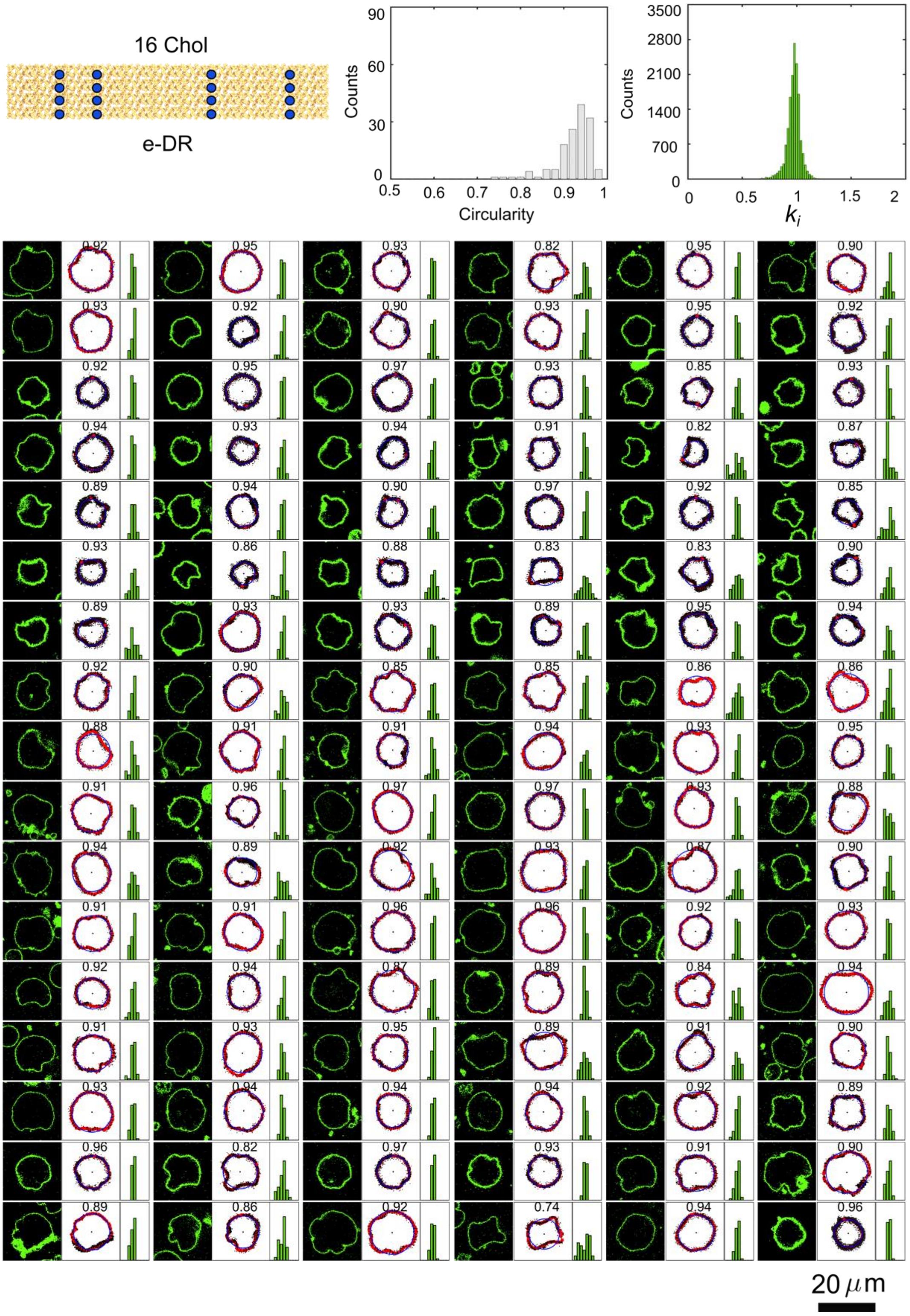

Data S50. 16 Chol e-DR-bound GUVs in isoosmotic buffer. The deformation efficiency is ~87.9%.

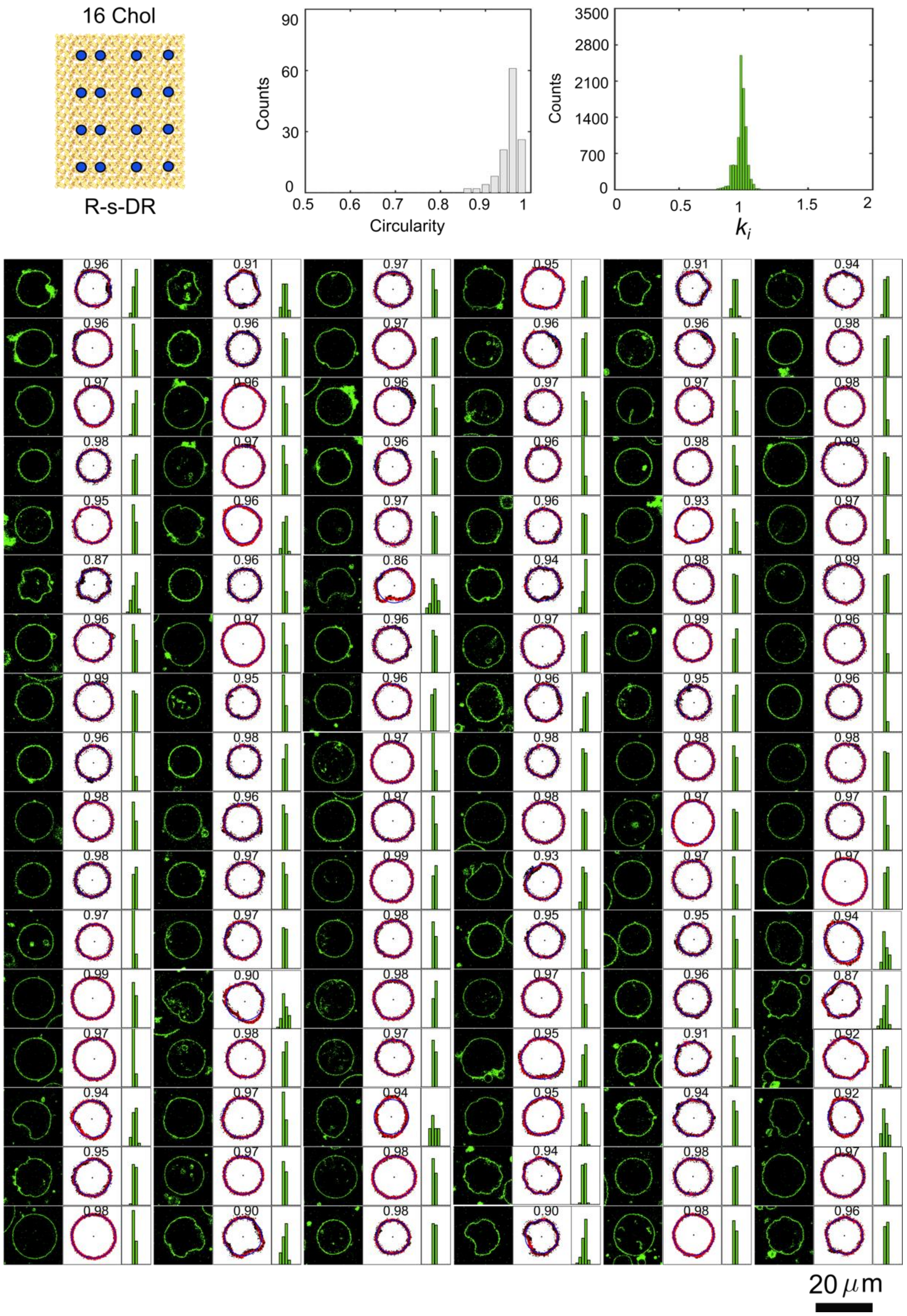

Data S51. 16 Chol R-s-DR-bound GUVs in isoosmotic buffer. The deformation efficiency is ~34.1%.
